# Reshaping the tumor microenvironment of cold soft-tissue sarcomas with anti-angiogenics: a phase 2 trial of regorafenib combined with avelumab

**DOI:** 10.1038/s41392-025-02278-9

**Published:** 2025-06-27

**Authors:** Maud Toulmonde, Jean-Philippe Guégan, Mariella Spalato-Ceruso, Thibaud Valentin, Rastilav Bahleda, Florent Peyraud, Christophe Rey, Michèle Kind, Coralie Cantarel, Carine Bellera, Lucile Vanhersecke, Alban Bessede, Antoine Italiano

**Affiliations:** 1 Department of Medical Oncology, Institut Bergonié, Bordeaux, France; 2 Explicyte, Bordeaux, France; 3 Department of Medicine, Oncopole, Toulouse, France; 4 DITEP, Gustave Roussy, Paris, France; 5 Department of Medical Imaging, Institut Bergonié, Bordeaux, France; 6 Inserm U1219, Bordeaux Population Health Research Center, EPICENE team, University of Bordeaux, Bordeaux, France; 7 Inserm CIC1401, Clinical and Epidemiological Research Unit, Institut Bergonié, Comprehensive Cancer Center, Bordeaux, France; 8 Department of Pathology, Institut Bergonié, Bordeaux, France; 9 Faculty of Medicine, University of Bordeaux, Bordeaux, France

**Keywords:** Cancer microenvironment, Sarcoma, Tumour immunology

## Abstract

The majority of sarcomas are under the influence of a tumor microenvironment that dampens immune activity, resulting in resistance to monoclonal antibodies targeting immune checkpoints and reduced clinical effectiveness. Preclinical studies indicate that targeting abnormal neoangiogenesis by inhibiting vascular endothelial growth factor receptor (VEGFR) can alter the TME, thereby promoting T cell infiltration and increasing tumor immunogenicity. The REGOMUNE study, a phase II clinical trial, assessed the therapeutic combination of regorafenib, a multityrosine kinase inhibitor that targets VEGFR2 and the PD-L1 blocker avelumab, in individuals with advanced “cold” STS characterized by a lack of mature tertiary lymphoid structures (mTLS). Forty-nine mTLS-negative STS patients were enrolled, including leiomyosarcoma (45%), synovial sarcoma (18%), and other subtypes. The objective response rate was 11.0% (95% CI: 4.0% - 22.0%), with median progression-free survival and overall survival of 1.8 months (95% CI, 1.7–3.5 months) and 15.1 months, respectively. Frequent adverse events included grade 1 or 2 palmar-plantar erythrodysesthesia, fatigue, and diarrhea. On-treatment multiplex immunofluorescence analysis revealed significant increases in CD8 + T cell and B cell infiltration and PD1 expression on immune cells. Plasma analysis indicated significant upregulation of soluble PD-L1 (sPD-L1) levels and tryptophan consumption. Overall, these results indicate that anti-angiogenic therapy modulates the tumor microenvironment in patients with cold STS and highlight the need for complementary strategies to enhance the functional activity of immune cells in this particular setting. Clinical trial registration number: NCT03475953

## Introduction

The current standard of care for patients with advanced or metastatic soft-tissue sarcomas (STS) remains largely dependent on conventional chemotherapy. However, its effectiveness is often limited, emphasizing the urgent need for novel therapeutic strategies. Immunotherapy has emerged as a promising alternative, with significant success in various malignancies. Advances in understanding the molecular mechanisms underlying STS immunity have identified key targets for immune-based interventions, further supporting the rationale for personalized treatments tailored to the tumor’s immune landscape.^1^ Among these approaches, immune checkpoint inhibitors (ICIs) have gained increasing attention for their potential role in sarcoma treatment, with growing clinical and translational significance.^2^

A pivotal study by Petitprez et al.^3^ characterized the STS immune contexture, establishing an immune categorization derived from transcriptomic analysis. This approach distinguishes five specific sarcoma immune classes, each associated with different extents of immune infiltration. Notably, their findings highlighted the existence of TLS in tumors classified as immune-high, linking them to better clinical outcomes and an increased likelihood of response to immunotherapy. TLS consists of lymphoid formations that develop ectopically, featuring components like B cell follicles, germinal centers, and organized T-cell areas, akin to lymph nodes.^4^ By promoting immune cell communication and boosting local immune defense, these formations are pivotal in antitumor immunity. The first immunotherapy trial in STS, informed by biomarker analysis, indicated that patients with TLS had a 30% response rate to ICIs.^5^ These findings underscore the potential of immunotherapy as a more effective alternative to conventional cytotoxic chemotherapy in select STS patients. However, a major challenge remains in broadening these advantages to the ~80% of patients whose tumors lack TLS.^3^ Addressing this limitation necessitates strategies aimed at transforming “cold” tumors into “hot,” immune-responsive microenvironments.

Preclinical studies have suggested that inhibiting vascular endothelial growth factor (VEGF) signaling may help reshape the tumor vasculature, thereby facilitating immune cell infiltration. However, these mechanisms remain complex and require further validation in sarcomas. Some studies indicate that low-dose VEGF inhibitors may enhance immune cell recruitment by normalizing the vasculature, rather than impairing blood flow and inducing hypoxia. Nonetheless, these effects have not been extensively explored in STS.^6,7^ This highlights the need for a deeper understanding of how VEGF inhibitors may optimize immunotherapy outcomes, particularly when combined with ICIs. Regorafenib, a multikinase inhibitor approved for advanced colorectal cancer, hepatocellular carcinoma, and gastrointestinal stromal tumors,^8^ has demonstrated clinical activity against placebo in advanced STS patients.^9–11^ Preclinical research suggests that regorafenib exerts immunomodulatory effects by altering immune cell function, regulating PD-L1 and MHC-I expression on tumor cells, and promoting normalization of aberrant tumor vasculature.^12^

Based on these findings, we hypothesize that regorafenib could enhance tumor sensitivity to PD-L1 inhibition in patients with advanced, cold STS that lack mature TLS (mTLS). Patient selection in this study was based on the absence of mTLS, given our previous findings that TLS-negative sarcomas derive minimal benefit from immunotherapy, regardless of histological subtype. This study evaluates the clinical efficacy of regorafenib in combination with avelumab in mTLS-negative STS patients while also providing insights into the biological changes induced by this treatment.

## Results

### Trial design and patients’ characteristics

**Fig. 1:**
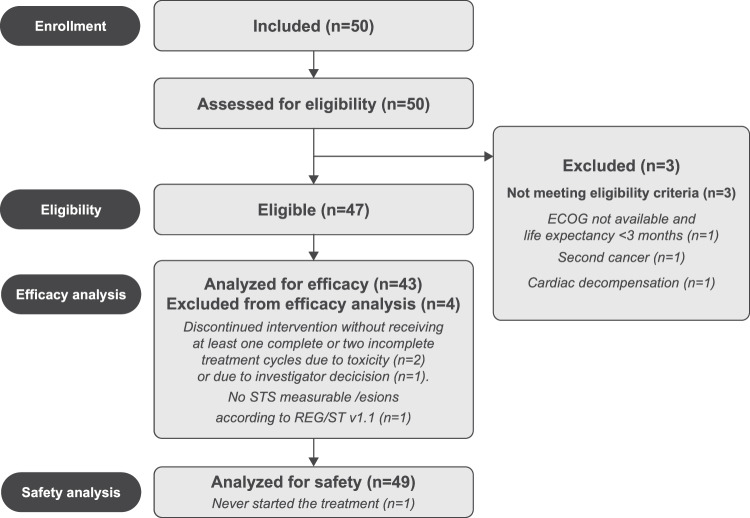
Flow chart of the REGOMUNE

Between May 14, 2019, and August 4, 2021, a total of 50 patients were enrolled in the study. However, three individuals did not satisfy the eligibility criteria and were therefore excluded from the analysis. Additionally, three more patients did not qualify based on efficacy criteria, resulting in 43 patients being deemed evaluable for the primary endpoint. (Fig. 1). Baseline patient characteristics are provided in Supplementary Table 1. The cohort was heavily pre-treated, with 19 patients (38.8%) having undergone more than two prior treatment lines. The median number of previous treatments was two, ranging from one to eight (Supplementary Table 1).

After a median follow-up of 7.1 months (95% CI 4.9–8), two patients (15.2%) were still receiving treatment, and 45 patients (84.8%) discontinued treatment. Discontinuation was related to disease progression in 40 cases (63.8%), investigator decision for 3 patients (4.2%), adverse event for 3 patients (10.6%), and death not related to treatment toxicity for 1 patient (4.2%) (Fig. 1).

### Efficacy

**Fig. 2:**
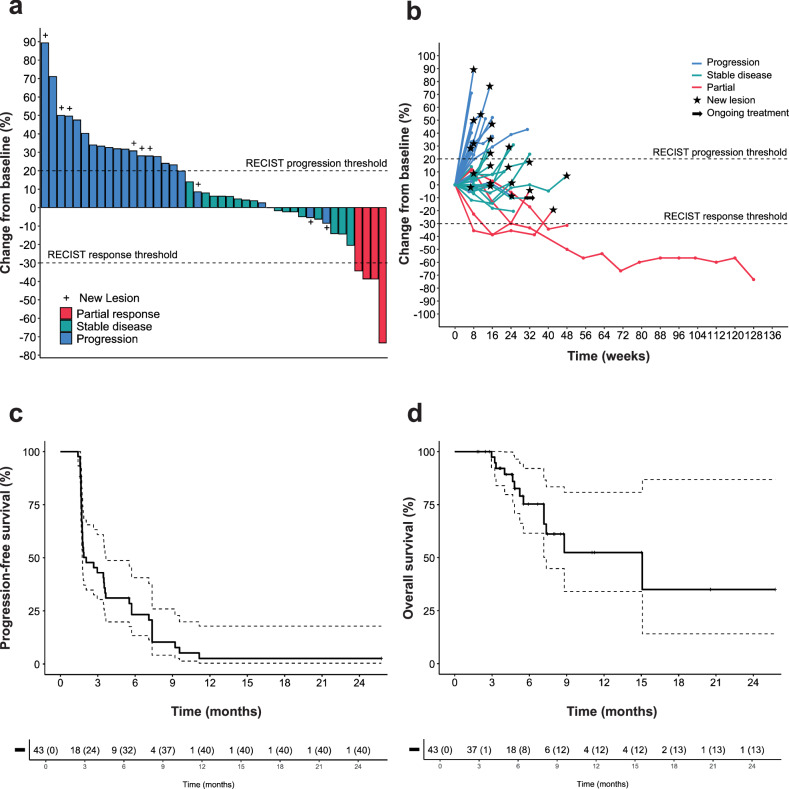
Efficacy of the combination Regorafenib-Avelumab in cold soft-tissue sarcoma patients. **a** Waterfall plot and **b** Spider plot of the maximum change in tumor size in patients with sarcoma treated with regorafenib plus avelumab and eligible for efficacy (*n* = 43). Kaplan–Meier curves of the progression-free survival (**c**) and overall survival (**d**). RECIST Response Evaluation Criteria in Solid Tumors

At each stage of the Bayesian efficacy analysis, the predefined futility criteria were never reached, enabling patient enrollment to proceed uninterrupted until the protocol-specified maximum number of participants was attained (*n* = 50). The Bayesian mean posterior of the objective response rate (ORR) under treatment was 11.0% (95% CI: 4.0%–22.0%). Of the 43 patients evaluated for efficacy (Figs. 1), 4 (11.4%) experienced a partial response (angiosarcoma, undifferentiated pleomorphic sarcoma, malignant peripheral nerve sheat tumor, synovial sarcoma), 17 (38.6%) had stable disease (1 dedifferentiated liposarcoma, 1 desmoplastic round cell tumor, endometrial stromal sarcoma, 8 leiomyosarcomas, rhabdomyosarcoma, 4 synovial sarcoma, 1 undifferentiated pleiomorphic sarcoma) and 22 (50.0%) showed progressive disease (1 dedifferentiated liposarcoma, 10 leiomyosarcoma, 2 myxoid liposarcoma, 3 undifferentiated pleiomorphic sarcoma, 2 solitary fibrous tumor, 4 synovial sarcoma), according to RECIST 1.1 criteria (Fig. 2a). Seven patients (16.3%) had objective response or stable disease lasting more than 6 months (angiosarcoma, undifferentiated pleomorphic sarcoma, malignant peripheral nerve sheat tumor, 2 synovial sarcoma, 2 leiomyosarcomas). Responses were durable, with a median duration of response of 7.8 months (95% CI: [3.8–NR]) (Fig. 2b). Median progression-free survival (PFS) was 1.8 months (95% CI, 1.7–3.5 months) (Fig. 2c). The 6-month and 1-year PFS rates were 22.1% (95% CI, 11.0–35.7%) and 4.1% (95% CI, 0.4–15.9%), respectively. The median overall survival (OS) was 15.1 months (95% CI: [7.2–NR]) reached, with the 6-month and 1-year OS rates at 75.3% (95% CI, 56.1–87.0) and 52.4% (95% CI, 28.3–71.9), respectively (Fig. 2d). Median growth-modulation index (GMI) was 0.54 with 6 patients (14.0%) presenting with a GMI ≥ 1.33 (Supplementary Fig. 1).

### Safety

A total of 49 patients were included in the adverse events assessment. Overall, the tolerability was good. Treatment-related adverse events and laboratory abnormalities observed in more than 5% of patients for grades 1 and 2, as well as all occurrences of grades 3 and 4, are summarized in Supplementary Table 2. The most commonly reported clinical toxicities associated with the treatment included fatigue, diarrhea, anorexia, and mucositis. Aligning with expectations, the prevailing treatment-related laboratory abnormalities were transaminitis and an increase in thyroid-stimulating hormone. Serious adverse events were observed in 25 patients, representing 52.1% of the study population. Treatment modifications due to drug-related adverse events were required for 40 patients (93.9%) receiving regorafenib, which included 29 temporary interruptions, 18 dose reductions, and 3 permanent discontinuations. Similarly, 22 patients (70%) on avelumab required adjustments, with 2 cases leading to permanent discontinuation. Notably, no treatment-related deaths were reported.

### Exploratory analysis of biomarkers

**Fig. 3:**
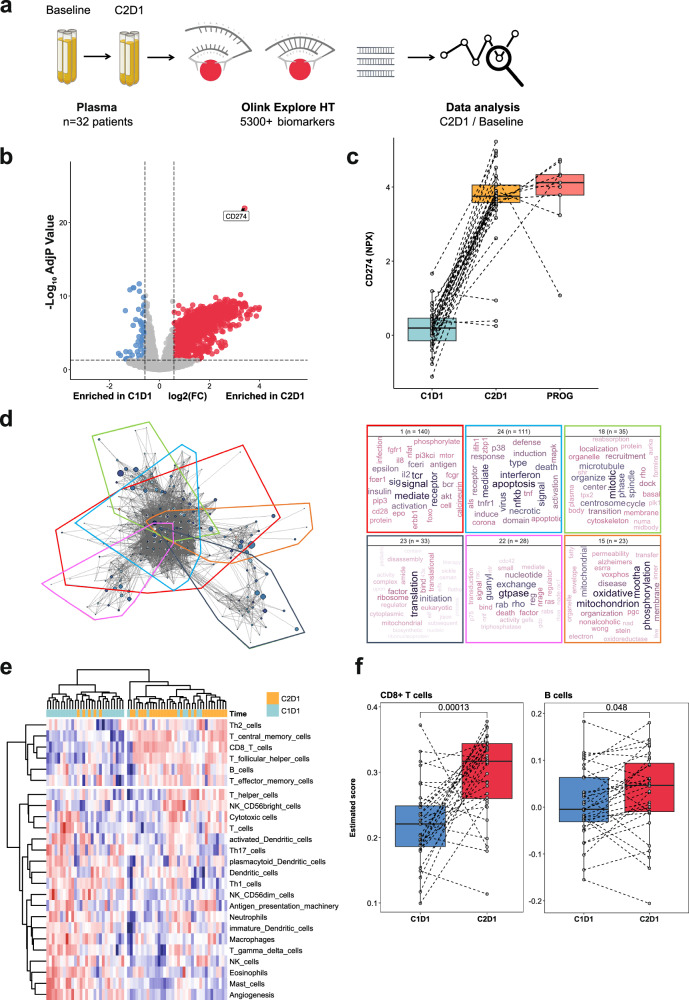
Regorafenib-Avelumab treatment triggers an immune response in cold soft-tissue sarcoma patients. **a** Flow chart illustrates the strategy used to identify biomarkers linked with Regorafenib-Avelumab treatment. Plasma samples collected at baseline and Cycle2 Day1 (C2D1) were analyzed using the Olink Explore HT panel. **b** Volcano plot showcases the differential protein expression in sarcoma patients upon treatment. **c** Boxplot representation of soluble CD274 (PD-L1) protein secretion. **d** Network visualization of GO term enrichment in patients treated with Regorafenib-Avelumab (left) and characterization of each identified cluster by text-mining (right). **e** Heatmap of immune cell estimation calculated by gsva in sarcoma patients upon treatment with Regorafenib and avelumab. **f** Boxplot representation of CD8+ T cells and B cells estimated proportions. *P* values were calculated using the Wilcoxon tests

**Fig. 4:**
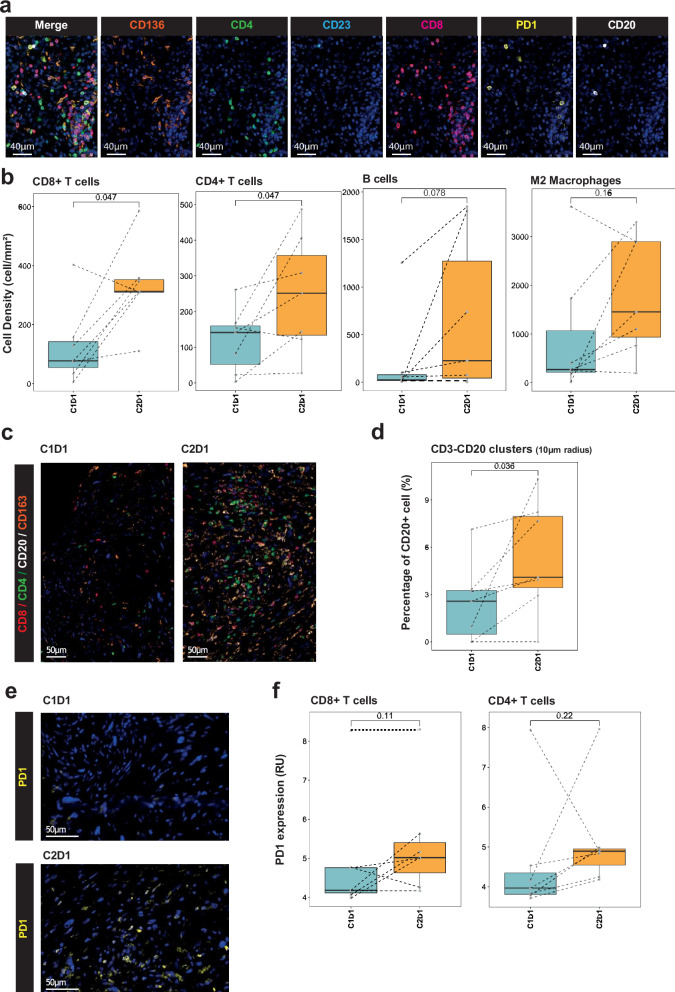
Regorafenib and avelumab treatment induce B and T cells infiltration in sarcoma patients. **a** Illustration of 7-plex immunohistofluorescence panel. **b** Boxplot representations of CD8+, CD4+, CD20+ (B cells) and CD163+ (M2 macrophages) cell densities. **c** Illustration of treatment-induced immune infiltration in one sarcoma patient with progressive disease. **d** Quantification of the percentage of CD20+ cells colocalized with CD3+ cells (CD4+ or CD8+) in a radius of 10 µm. **e** Illustration of PD-1 expression upon treatment. **f** Boxplot representations of PD-1 expression in CD8+ and CD4+ T cells. All the *p* values were calculated using Wilcoxon tests

Proteomic profiling of plasma samples was carried out to examine blood-derived immune biomarkers. The concentrations of ≈5300 proteins were measured at baseline and at Cycle 2 day 1 using the Olink Explore HT panel (Fig. 3a). Comparison of proteins differentially expressed upon treatment highlighted that soluble PD-L1 was the most significantly upregulated protein (Fig. 3b, c). Gene Ontology (GO) term analysis of proteins upregulated post-treatment revealed the enrichment of immune processes, and notably the T cell receptor signaling pathway (Fig. 3d). We then used these proteomic data to infer the evolution of the relative abundance of different immune cell types in the tumor microenvironment (TME). We observed a significant increase in CD8 T cells and B cells, thereby reflecting immune induction through treatment (Fig. 3e, f). Additionally, the metabolomic analysis of plasma showed that the treatment decreased the levels of the essential amino acid, L-Tryptophan, which is crucial for supporting an effective immune response (Supplementary Fig. 2).^13^ Preclinical research has demonstrated that the blockade of the VEGF/VEGFR2 signaling may act synergistically with immune checkpoint inhibition, enhancing the density of CD8+ T cells within the TME. To investigate this, seven patients consented to undergo sequential biopsies taken both at baseline and on Day 1 of Cycle 2 (Fig. 4a). When compared to biopsies collected at baseline, an increase in CD8+ T cell and B cell infiltration was observed in six and four patients, respectively, in the on-treatment samples. Additionally, an increase in CD4+ T cells and M2 macrophage infiltration was noted in five patients (Fig. 4b, c). Analysis of cell neighboring revealed that B cells were also closer to CD3+ T cells after treatment (Fig. 4d). Finally, increased PD-1 expression was observed on T cells (Fig. 4e, f). However, analysis of the spatial distribution of these immune cells showed no induction of TLS. It’s worth noting that the increase in immune cell infiltration did not correlate with clinical outcomes.

## Discussion

This report outlines the phase II trial outcomes evaluating the influence of anti-angiogenic therapy on the TME in patients with TLS-negative STS. The study marks an important advancement in the field, providing new insights into the potential of anti-angiogenic agents to modify the TME and enhance tumor responsiveness to immune-mediated destruction.

In the burgeoning field of oncology, the strategic integration of tyrosine kinase inhibitors (TKIs) with ICIs has opened new avenues for the treatment of various cancers. While studies such as Wilky et al. have demonstrated the efficacy of combining TKIs with ICIs in sarcoma, these studies, including those focused on alveolar soft part sarcoma (ASPS),^14–17^ did not stratify patients based on the immunogenic characteristics of their TME. ASPS, for instance, is an ultra-rare subtype known for its high sensitivity to ICIs, making it a notable exception rather than the rule in sarcoma therapy.

In contrast, our study specifically targets TLS-negative sarcomas, which represent the majority of STS cases and are characterized by a “cold” TME that is resistant to immunotherapy. Our goal was to investigate whether combining regorafenib, a TKI, with avelumab, a PD-L1 inhibitor, could induce an immune response and potentially convert these cold tumors into a more “inflamed” state. Unlike previous studies, which did not account for the immunological diversity of sarcoma subtypes, our approach focuses on the TME, recognizing its pivotal role in dictating response to immunotherapy. This distinction highlights the novelty of our study.

The ORR observed in our study is lower compared to the aforementioned studies and highlights the profound impact of TME characteristics on treatment success. Our findings underscore the challenges of treating “cold” sarcomas and suggest that the immunological quiescence of these tumors may inherently limit the effectiveness of current immunotherapeutic strategies. This is also highlighted by the results from the study by Cho et al., who investigated the efficacy of pazopanib and durvalumab in 47 patients with advanced STS.^16^ By using transcriptomic and multiplex IHC analyses, the authors found that the B cell lineage, a key feature of TLS, was the most significant predictor of PFS and response.^16^

None of the previous studies investigating anti-angiogenics combined with immune checkpoint inhibition in sarcomas have examined the combination’s impact on the TME. Our analysis of paired tissue and plasma samples demonstrated the dynamic immunomodulation induced by combining an anti-angiogenic agent with a PD-1 inhibitor, showing the potential of this therapeutic approach to enhance immune cell infiltration in “cold” sarcomas.

Our findings contrast with those of the SARC028 study, where PD-1 monotherapy with pembrolizumab led to minimal changes in immune cell infiltration in advanced sarcoma patients. Specifically, the analysis of paired pre- and on-treatment biopsies from 68 patients showed no increase in T cell infiltration at 8 weeks (mean T cell density decreased from 180.5 cells/mm² at baseline to 123.1 cells/mm² on-treatment, *p* > 0.05; mean cytotoxic T cell density declined from 60.4 cells/mm² at baseline to 40.7 cells/mm² on-treatment, *p* > 0.05).^18^

In contrast, in our study, the combination of PD-L1 inhibition with regorafenib significantly boosted immune infiltration, with marked increases in CD8+ T cells and B cells, alongside elevated PD-1 expression. This suggests that regorafenib may promote a more immune-permissive environment by normalizing vasculature and enhancing immune cell trafficking to the tumor, potentially transforming “cold” sarcomas, typically lacking tertiary lymphoid structures, into more “hot” or immune-responsive states. Such immunomodulatory effects underscore regorafenib’s potential as an adjuvant to PD-1 inhibitors in sarcomas, especially where single-agent checkpoint inhibitors do not sufficiently activate the immune response.

These observations align with preclinical findings suggesting that inhibiting angiogenesis could enhance the immunogenic potential of initially immune-silent tumors.^19^ However, this increase in immune cell density did not translate into substantial clinical activity, as progressive disease occurred even in patients with the highest increases in CD8+ T and B cell density. This indicates that merely boosting immune cell numbers is insufficient for an effective response. The functional state of these cells—such as their exhaustion status, organization into structured formations, and capacity to engage and eliminate tumor cells—is essential.^20^ Notably, our study observed increased PD-1 expression on T cells with treatment. While severely exhausted CD8+ T cells with high PD-1 expression do not respond to PD-1 blockade, those with intermediate PD-1 levels are more likely to benefit from anti-PD-1 therapy.

By analyzing the proteomic profile of the patients’ plasma, we also observed that the most significantly upregulated protein on treatment was the soluble form of PD-L1 (sPD-L1, CD274). Several studies have already shown the negative predictive value of soluble PD-L1 for response to immune checkpoint inhibition.^21^ The exact sources and mechanisms by which increased levels of sPD-L1 negatively impact immunotherapy are not entirely clear. One hypothesis is that sPD-L1 binds to PD-1 on CD8 T cells, delivering an inhibitory signal that suppresses their cytotoxic function, thereby aiding tumor immune evasion. In the context of the increased expression of PD-1 by T cells that we observed, we can speculate that the overactivation of the PD-1/PD-L1 signaling axis is a major contributor to the resistance to treatment observed in our study. The abundance of M2 macrophages within the tumor may contribute to immunosuppression and a lack of treatment response.^22^ Additionally, low levels of the essential amino acid L-Tryptophan in plasma might induce an anergic state in immune cells. It is well-known that Tryptophan metabolism through the Kynurenine pathway and activation of the Aryl hydrocarbon receptor promote immune evasion.^23^ A concurrent study on the same treatment regimen in patients with advanced gastroenteropancreatic neuroendocrine tumors has revealed a significant association between heightened Kynurenine pathway activation and poor clinical outcomes.^24^ This suggests that Kynurenine pathway activation might also explain the reduced sensitivity observed in sarcoma patients.

Many patients in our study required dose reductions or temporary discontinuations of regorafenib due to adverse reactions, a finding that aligns with previous clinical trials using regorafenib as monotherapy. However, our safety data did not indicate any increased toxicity when regorafenib was combined with avelumab, compared to their use as individual agents.

The optimal dose of anti-angiogenic agents in combination with ICIs remains an area of active research. It is possible that VEGF-mediated immunosuppression and angiogenesis could be inhibited effectively through different dosing strategies. Some evidence suggests that fine-tuning the level of VEGF inhibition may be key to balancing angiogenesis suppression with minimizing adverse effects such as excessive vascular pruning and hypoxia.^25^ For instance, preclinical models have shown that lower doses of anti-VEGFR2 antibodies or TNFα can lead to a more favorable TME, characterized by an increase in effector T cells and a shift towards a pro-immune macrophage profile (M1), as opposed to the effects seen with higher doses.^26,27^

Additionally, recent results from an open-label trial involving metastatic colorectal cancer patients explored a dose-escalation strategy for regorafenib, starting at 80 mg/day and increasing weekly by 40 mg up to 160 mg/day, based on tolerance. This approach appeared to maintain efficacy while reducing adverse events compared to the standard 160 mg/day dosing regimen for 21 days out of a 28-day cycle.^28^ While this strategy was not evaluated in our study, it underscores the ongoing need to refine dosing strategies to optimize the balance between efficacy and tolerability.

One of the primary limitations of our study is the absence of a control arm, which restricts our ability to directly compare the efficacy of the combination therapy against monotherapy or standard of care treatments. Additionally, the relatively small sample size and the heterogeneous nature of the sarcoma subtypes enrolled may limit the generalizability of our findings across the broader STS population. However, our study, which focused on a specific group of sarcomas characterized from a microenvironmental perspective (absence of mTLS), offers crucial insights into enhancing immunotherapy efficacy in this indication. Despite the constraints imposed by the sample size, which restricted a detailed subgroup analysis based on STS histological subtypes, these findings highlight the need for further research. Upcoming research should be adequately designed to comprehensively assess the effects of combining anti-angiogenic agents with ICIs across various STS subtypes. Recognizing this limitation, the potential role of strategies that induce TLS formation, such as LTBR or CD40 agonists, emerges as particularly promising.^29,30^ These strategies have the potential to profoundly reshape the TME, creating new opportunities for more effective and personalized treatments tailored to patients with advanced “cold” STS.

## Patients and methods

### Study design and participants

The REGOMUNE trial is a multi-cohort study comprising 17 parallel, single-arm phase 2 trials, with the purpose of evaluating the safety and efficacy of combining regorafenib with avelumab across various tumor types. For the STS cohort, eligible patients had to be at least 18 years old with histologically confirmed advanced or metastatic STS, characterized by the absence of TLS, as determined by central review. The histological diagnosis was performed by an expert pathologist, based on central review and following the WHO classification of soft-tissue tumors.^31^ Additional eligibility criteria included an ECOG performance status of 0–1, disease suitable for measurement as outlined in RECIST 1.1,^32,33^ a minimum of one previous systemic treatment, and adequate hematologic, renal, metabolic, and hepatic function.

A comprehensive list of eligibility criteria is outlined in the study protocol. Prior to enrollment, participants underwent an extensive blood analysis, which included assessments of blood cell counts, liver enzymes (alanine aminotransferase, aspartate aminotransferase, alkaline phosphatase), albumin, bilirubin, lipase, creatine phosphokinase, coagulation markers, creatinine, and urea nitrogen levels. Key exclusion criteria included prior treatment with avelumab, regorafenib, or other ICIs such as anti-PD-1, anti-PD-L1, anti-PD-L2, anti-CD137, or anti-CTLA-4 antibodies.

The REGOMUNE study was conducted in compliance with the ethical guidelines set forth by the Declaration of Helsinki. Approval for the trial protocol, which included an evaluation of potential risks and benefits for participants, was obtained from a Central Institutional Review Board (Comité de Protection des Personnes Sud-Est II, Lyon, France, reference number 2016-005175-27), in accordance with French regulatory requirements. All patients provided written informed consent before participation.

Once deemed eligible, patients commenced treatment with regorafenib at a daily dose of 160 mg, following a 3 weeks on, 1 week off cycle within each 28-day treatment cycle. Avelumab was introduced on day 15 of the first cycle, administered biweekly via intravenous infusion at a dose of 10 mg/kg. Treatment continued until disease progression, unacceptable toxicity, investigator decision, or patient withdrawal of consent. Routine follow-up visits and laboratory tests were scheduled to monitor adverse events, which were classified according to the NCI Common Terminology Criteria for Adverse Events (CTCAE) version 5.0.

Dose adjustments for regorafenib were allowed to address safety issues. The starting dosage of 160 mg could be decreased initially to 120 mg and subsequently to 80 mg based on patient tolerance. If treatment interruptions lasted more than 4 weeks, regorafenib had to be permanently stopped; however, patients could remain on avelumab if clinically appropriate. In contrast, reducing the dosage of avelumab was not allowed, although temporary treatment pauses were permitted in response to immune-related adverse reactions. Patients experiencing ≥2 consecutive interruptions of avelumab were required to permanently discontinue it, although they could continue regorafenib.

Treatment efficacy was assessed based on RECIST version 1.1 criteria every 8 weeks. All assessments were independently reviewed by a blinded central committee. Pre- and on-treatment (day 1 of cycle 2) tumor tissue and blood samples were obtained from consenting patients. These samples underwent analysis to investigate how the treatment affected the TME and to detect changes in the plasma proteomic and metabolomic profiles.

### Clinical outcomes

The primary objective of this clinical trial was the 6-month ORR, which was defined as the proportion of patients achieving an objective tumor response according to a modified version of RECIST 1.1 guidelines,^32^ confirmed through centralized radiological evaluation. Secondary endpoints included the best overall response achieved at any point during treatment, the 6-month ORR, the 6-month progression-free rate, PFS at 1 year, OS at 1 year, the GMI, safety profiles, and exploratory biomarker analyses. The best overall response was determined by identifying the most favorable response observed during the treatment period. PFS was defined as the interval from the start of study treatment until documented disease progression or death from any cause. OS was calculated from the start of treatment until death from any cause. GMI represented the ratio between the PFS obtained with the current investigational treatment and the PFS recorded from the patient’s previous line of therapy, as previously described.^31^ Safety assessments were conducted and categorized according to the National Cancer Institute’s Common Toxicity Criteria (CTCAE), version 5.0.

### Multiplex immuno-fluorescence

Multiplexed immunohistofluorescence was conducted with a validated multiplex panel combining the following markers CD8 (C8/144B, Dako), CD163 (10D6, Novocastra), PD-1 (NAT105, Cell Marque), CD4 (SP35, Ventana), CD23 (SP23, Ventana) and CD20 (L26, Ventana). Bound primary antibodies were detected using OmniMap HRP-conjugated anti-Rabbit IgG (760-4311, Ventana) or OmniMap HRP-conjugated anti-mouse IgG (760-4310, Ventana) detection kits, followed by tyramide signal amplification using opal fluorophores (Opal 480, Opal 520, Opal 570, Opal 620, Opal 690 and/or Opal 780; Akoya Biosciences).

After staining, slides underwent counterstaining with spectral DAPI (Akoya Bioscience), followed by the placement of coverslips. The prepared slides were then scanned and digitized with the PhenoImager HT System (Akoya). Multispectral image data were analyzed using Qupath software (version 0.5.0) after tumor regions had been delineated by a qualified pathologist. Tissue segmentation was performed, and individual cells were identified based on DAPI staining. Mean intensity values for each marker were then calculated for each identified cell. The extracted signal intensities were normalized using the GaussNorm function available in the flowstat package for R. Finally, cell phenotyping was performed by applying a threshold-based approach utilizing FlowJo software (version 10.8.1).

### PD-L1 staining

Tumor tissue was immunostained for PD-L1 with the QR-1 antibody clone (Diagomics). Briefly, staining was conducted on the Ventana Discovery system (Roche, Ventana) according to the manufacturer’s recommended RUO Discovery Universal protocol. Detection was performed using the OmniMap anti-Rabbit HRP detection kit (760-4311, Ventana). Following staining, digital images were obtained utilizing the PhenoImager HT imaging platform (Akoya).

### Plasma proteomics

Plasma proteomic analysis was performed utilizing the Olink Proximity Extension Assay (Olink Proteomics AB, Uppsala, Sweden). Briefly, pairs of oligonucleotide-labeled antibody probes binding to their target proteins initiated the hybridization of oligonucleotides in a pair-wise manner. This process generated a unique DNA reporter sequence that is quantified by next-generation sequencing on a Novaseq 6000 system. Analysis of samples has been carried out using the Olink® Explore HT panel (Olink Proteomics AB, Uppsala, Sweden). Data underwent quality control and normalization using the plate controls. The final assay read-out is presented as NPX values, an arbitrary unit on a log2-scale where higher values correspond to elevated protein expression. Detailed assay validation data, including detection limits and intra- and inter-assay precision data, are accessible on the manufacturer’s website (www.olink.com).

Differential protein expression and gene sets enrichment analyses were performed using the “Limma” R package (v3.60.3). The packages igraph (v2.0.3) and vissE (v1.12.0) were used to perform clustering on the enriched gene sets and data visualization. Immune cell estimation was analyzed using the consensusTME R package (v0.0.1.9) with the Bindea and colleagues^34^ gene sets.

### Plasma metabolomics

The metabolomic analysis of plasma was performed using liquid chromatography–mass spectrometry (LC-MS). Metabolites were extracted from 50 µL of plasma by adding 200 µL of methanol, vortexing the mixture, sonicating it for 5 min, and then placing it on ice for 90 min to allow protein precipitation. The samples were centrifuged for 15 min at 20,000 × *g*, and the supernatants were collected and dried under nitrogen. The dried extracts were dissolved in either H2O/ACN (95/5%) for C18 analysis or ammonium carbonate (10 mM, pH 10.5)/ACN (40/60%) for HILIC analysis. Ultra-high-performance liquid chromatography (UHPLC) was performed on a Hypersil GOLD C8 column at 30 °C, and HPLC was performed on a Sequant ZICpHILIC column at 15 °C. The mobile phases for the RP column were 100% water (phase A) and 100% ACN (phase B), both containing 0.1% formic acid. For HILIC, phase A was 10 mM ammonium carbonate in water, and phase B was pure ACN. Chromatographic elution was done under gradient conditions with flow rates of 500 µL/min for the RP system and 200 µL/min for the HILIC system. LC-MS analyses were conducted using a U3000 liquid chromatography system coupled to an Exactive mass spectrometer with an electrospray source operating in both positive and negative ion modes. Data acquisition was managed with Xcalibur software (version 2.1). Automatic peak detection and integration were carried out using the XCMS software package, producing a data matrix with *m*/*z* and retention time values, and concentrations expressed as chromatographic peak areas. XCMS features were filtered based on correlation, repeatability, and quality criteria, and were annotated by matching measured masses with theoretical masses in biochemical and metabolomic databases using an R language tool.

### Statistical analysis

Our study adopted a Bayesian adaptive trial design, enabling a more efficient and dynamic approach to clinical research by allowing for continual learning and decision-making adjustments. This methodology contrasts with traditional trial designs, which often operate under fixed sample sizes and offer limited flexibility for modifications. Our analyses followed a sequential pattern, initiating with interim analyses after a 16-week observation of the initial 10 participants, followed by further assessments for every subsequent group of 5 patients. Success probability, defined as the ORR under treatment, was derived using a beta-binomial model as detailed by Zohar et al.^35^

At each interim analysis, a specifically defined stopping rule applied predictive probabilities to guide the continuation of the trial: if the predictive probability that the ORR fell at or below 10% reached 80% or more, indicating minimal response, we considered halting the study for inefficacy. Conversely, the trial would also be recommended for cessation for efficacy if the predictive probability that the ORR met or exceeded 25% reached 80% or higher. The primary endpoint, ORR under treatment, was expressed through the Bayesian mean posterior distribution along with a 95% credible interval.

Survival outcomes were scrutinized using the Kaplan–Meier method, reporting median survival rates and their 95% confidence intervals.

Exploratory analyses for biomarkers were conducted in R (v4.4.1).

## Supplementary information

Supplementary Tables and Figures

Study Protocol

## Data Availability

De-identified individual participant data supporting the findings described in this article will be accessible following publication for a period of up to 6 years. Data will be provided to researchers submitting scientifically robust proposals. Interested researchers should contact the corresponding author directly to request access.

## References

[1] Spalato-Ceruso, M., Ghazzi, N. E. & Italiano, A. New strategies in soft tissue sarcoma treatment. *J. Hematol. Oncol.* **17**, 761 (2024). 10.1186/s13045-024-01580-3 PMC11368005 39218932

[2] Tazzari, M. et al. Molecular determinants of soft tissue sarcoma immunity: targets for immune intervention. *Int. J. Mol. Sci.* **22**, 7518 (2021). 34299136 10.3390/ijms22147518 PMC8303572

[3] Petitprez, F. et al. B cells are associated with survival and immunotherapy response in sarcoma. *Nature* **577**, 556–560 (2020). 31942077 10.1038/s41586-019-1906-8

[4] Fridman, W. H. et al. B cells and tertiary lymphoid structures as determinants of tumour immune contexture and clinical outcome. *Nat. Rev. Clin. Oncol.* **19**, 441–457 (2022). 35365796 10.1038/s41571-022-00619-z

[5] Italiano, A. et al. Pembrolizumab in soft-tissue sarcomas with tertiary lymphoid structures: a phase 2 PEMBROSARC trial cohort. *Nat. Med.* **28**, 1199–1206 (2022). 35618839 10.1038/s41591-022-01821-3

[6] Georganaki, M., van Hooren, L. & Dimberg, A. Vascular targeting to increase the efficiency of immune checkpoint blockade in cancer. *Front. Immunol.* **9**, 3081 (2018). 30627131 10.3389/fimmu.2018.03081 PMC6309238

[7] Huang, Y. et al. Improving immune-vascular crosstalk for cancer immunotherapy. *Nat. Rev. Immunol.* **18**, 195–203 (2018). 29332937 10.1038/nri.2017.145 PMC5922422

[8] Ettrich, T. J. & Seufferlein, T. Regorafenib. *Recent Results Cancer Res.* **211**, 45–56 (2018). 30069758 10.1007/978-3-319-91442-8_3

[9] Brodowicz, T. et al. Efficacy and safety of regorafenib compared to placebo and to post-cross-over regorafenib in advanced non-adipocytic soft tissue sarcoma. *Eur. J. Cancer* **99**, 28–36 (2018). 29902612 10.1016/j.ejca.2018.05.008

[10] Blay, J. Y., Duffaud, F., George, S., Maki, R. G. & Penel, N. Regorafenib for the treatment of sarcoma. *Curr. Treat. Options Oncol.* **23**, 1477–1502 (2022). 36178573 10.1007/s11864-022-00990-0

[11] Mir, O. et al. Safety and efficacy of regorafenib in patients with advanced soft tissue sarcoma (REGOSARC): a randomised, double-blind, placebo-controlled, phase 2 trial. *Lancet Oncol.* **17**, 1732–1742 (2016). 27751846 10.1016/S1470-2045(16)30507-1

[12] Liu, J. et al. Immunomodulatory effects of regorafenib: enhancing the efficacy of anti-PD-1/PD-L1 therapy. *Front. Immunol.* **13**, 992611 (2022). 36119072 10.3389/fimmu.2022.992611 PMC9479218

[13] Bessede, A. et al. Aryl hydrocarbon receptor control of a disease tolerance defence pathway. *Nature* **511**, 184–190 (2014). 24930766 10.1038/nature13323 PMC4098076

[14] Wilky, B. A. et al. Axitinib plus pembrolizumab in patients with advanced sarcomas including alveolar soft-part sarcoma: a single-centre, single-arm, phase 2 trial. *Lancet Oncol.* **20**, 837–848 (2019). 31078463 10.1016/S1470-2045(19)30153-6

[15] Martin-Broto, J. et al. Nivolumab and sunitinib combination in advanced soft tissue sarcomas: a multicenter, single-arm, phase Ib/II trial. *J. Immunother. Cancer* **8**, e001561 (2020). 33203665 10.1136/jitc-2020-001561 PMC7674086

[16] Cho, H. J. et al. Durvalumab plus pazopanib combination in patients with advanced soft tissue sarcomas: a phase II trial. *Nat. Commun.* **15**, 685 (2024). 38263321 10.1038/s41467-024-44875-2 PMC10806253

[17] Liu, J. et al. Phase II study of TQB2450, a novel PD-L1 antibody, in combination with anlotinib in patients with locally advanced or metastatic soft tissue sarcoma. *Clin. Cancer Res.* **28**, 3473–3479 (2022). 35675031 10.1158/1078-0432.CCR-22-0871 PMC9662895

[18] Keung, E. Z. et al. Correlative analyses of the SARC028 trial reveal an association between sarcoma-associated immune infiltrate and response to pembrolizumab. *Clin. Cancer Res.* **26**, 1258–1266 (2020). 31900276 10.1158/1078-0432.CCR-19-1824 PMC7731262

[19] Tu, J. et al. The application and research progress of anti-angiogenesis therapy in tumor immunotherapy. *Front. Immunol.* **14**, 1198972 (2023). 37334350 10.3389/fimmu.2023.1198972 PMC10272381

[20] Chakravarti, M. et al. Terminally exhausted CD8+ T cells resistant to PD-1 blockade promote generation and maintenance of aggressive cancer stem cells. *Cancer Res.* **83**, 1815–1833 (2023). 36971604 10.1158/0008-5472.CAN-22-3864

[21] Oh, S. Y. et al. Soluble PD-L1 is a predictive and prognostic biomarker in advanced cancer patients who receive immune checkpoint blockade treatment. *Sci. Rep.* **11**, 19712 (2021). 34611279 10.1038/s41598-021-99311-y PMC8492653

[22] Toulmonde, M. et al. Use of PD-1 targeting, macrophage infiltration, and IDO pathway activation in sarcomas: a phase 2 clinical trial. *JAMA Oncol.* **4**, 93–97 (2018). 28662235 10.1001/jamaoncol.2017.1617 PMC5833654

[23] Badawy, A. A. Kynurenine pathway of tryptophan metabolism: regulatory and functional aspects. *Int. J. Tryptophan Res.* **10**, 1178646917691938 (2017). 28469468 10.1177/1178646917691938 PMC5398323

[24] Cousin, S. et al. Regorafenib plus avelumab in advanced gastroenteropancreatic neuroendocrine neoplasms: a phase 2 trial and correlative analysis. *Nat. Cancer* **6**, 584–594 (2025). 10.1038/s43018-025-00916-3 40204996

[25] Hato, T., Zhu, A. X. & Duda, D. G. Rationally combining anti-VEGF therapy with checkpoint inhibitors in hepatocellular carcinoma. *Immunotherapy* **8**, 299–313 (2016). 26865127 10.2217/imt.15.126 PMC5619018

[26] Huang, Y. et al. Vascular normalizing doses of antiangiogenic treatment reprogram the immunosuppressive tumor microenvironment and enhance immunotherapy. *Proc. Natl. Acad. Sci. USA* **109**, 17561–17566 (2012). 23045683 10.1073/pnas.1215397109 PMC3491458

[27] Johansson, A., Hamzah, J., Payne, C. J. & Ganss, R. Tumor-targeted TNFα stabilizes tumor vessels and enhances active immunotherapy. *Proc. Natl. Acad. Sci. USA* **109**, 7841–7846 (2012). 22547817 10.1073/pnas.1118296109 PMC3356673

[28] Bekaii-Saab, T. S. et al. Regorafenib dose-optimisation in patients with refractory metastatic colorectal cancer (ReDOS): a randomised, multicentre, open-label, phase 2 study. *Lancet Oncol.* **20**, 1070–1082 (2019). 31262657 10.1016/S1470-2045(19)30272-4 PMC9187307

[29] Van Hooren, L. et al. Agonistic CD40 therapy induces tertiary lymphoid structures but impairs responses to checkpoint blockade in glioma. *Nat. Commun.* **12**, 4127 (2021). 34226552 10.1038/s41467-021-24347-7 PMC8257767

[30] Byrne, K. T. et al. Neoadjuvant selicrelumab, an agonist CD40 antibody, induces changes in the tumor microenvironment in patients with resectable pancreatic cancer. *Clin. Cancer Res.* **27**, 4574–4586 (2021). 34112709 10.1158/1078-0432.CCR-21-1047 PMC8667686

[31] WHO Classification of Tumours Editorial Board. *Soft Tissue and Bone Tumours* WHO Classification of Tumours Series, 5th edn, Vol. 3 (International Agency for Research on Cancer, 2020).

[32] Eisenhauer, E. A. et al. New response evaluation criteria in solid tumours: revised RECIST guideline (version 1.1). *Eur. J. Cancer* **45**, 228–247 (2009). 19097774 10.1016/j.ejca.2008.10.026

[33] Von Hoff, D. D. There are no bad anticancer agents, only bad clinical trial designs—twenty-first Richard and Hinda Rosenthal Foundation Award Lecture. *Clin. Cancer Res.* **4**, 1079–1086 (1998). 9607564

[34] Bindea, G. et al. Spatiotemporal dynamics of intratumoral immune cells reveal the immune landscape in human cancer. *Immunity* **39**, 782–795 (2013). 24138885 10.1016/j.immuni.2013.10.003

[35] Zohar, S., Teramukai, S. & Zhou, Y. Bayesian design and conduct of phase II single-arm clinical trials with binary outcomes: a tutorial. *Contemp. Clin. Trials* **29**, 608–616 (2008). 18201945 10.1016/j.cct.2007.11.005

