# Supplementary material for: Reshaping the tumor microenvironment of cold soft-tissue sarcomas with anti-angiogenics: a phase 2 trial of regorafenib combined with avelumab
Source: Signal Transduct Target Ther. 2025 Jun 27;10:202. doi: 10.1038/s41392-025-02278-9 (PMC12205094; doi:10.1038/s41392-025-02278-9)
Supplement: Supplementary file 1 — Supplementary Tables and Figures [file 41392_2025_2278_MOESM1_ESM.docx]

Supplementary Materials for

**Reshaping the tumor microenvironment of cold** **soft-tissue sarcomas with anti-angiogenics: a Phase 2 Trial of regorafenib combined with avelumab**

Maud Toulmonde^1^, MD, PhD*,  Jean-Philippe Guegan^2^*, PhD; Mariella Spalato-Ceruso, MD^1^ ; Thibaud Valentin^3^, MD; Rastilav Bahleda^4^, MD; Florent Peyraud^1^, MD;  Christophe Rey^2^, PhD ; Michèle Kind^5^, MD ; Coralie Cantarel^6,7^, MSc ; Carine Bellera^6,7^, PhD ; Lucile Vanhersecke^8^, MD ; Alban Bessede^2^, PhD ; Antoine Italiano^1,9^,MD, PhD.

Correspondence to: a.italiano@bordeaux.unicancer.fr

**This PDF file includes:**

Figures. S1 to S2

Tables S1 to S2


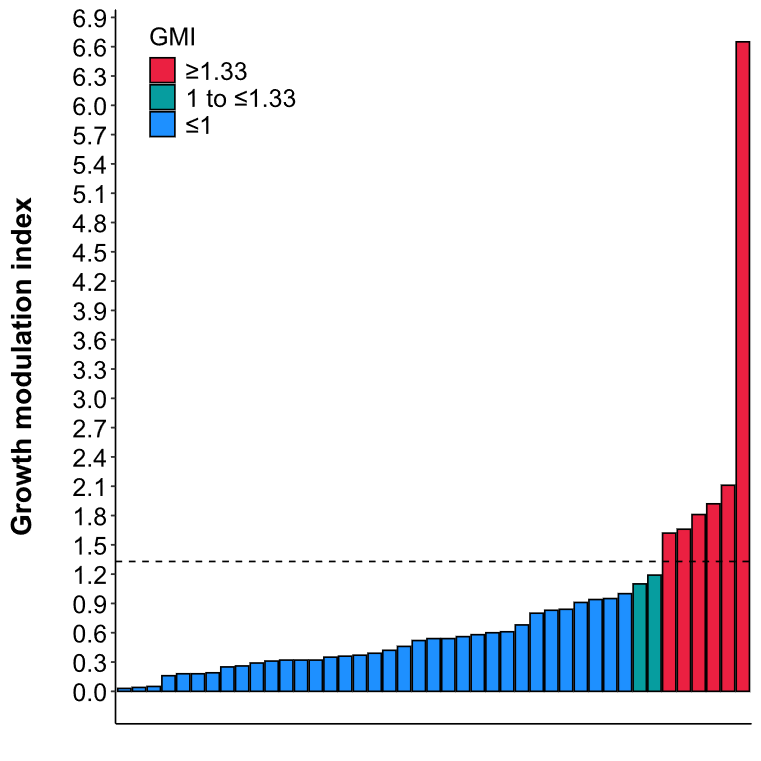


Figure. S1.

Waterfall plot depicting the growth modulation index (GMI) in patients with soft-tissue sarcomas receiving regorafenib and avelumab.


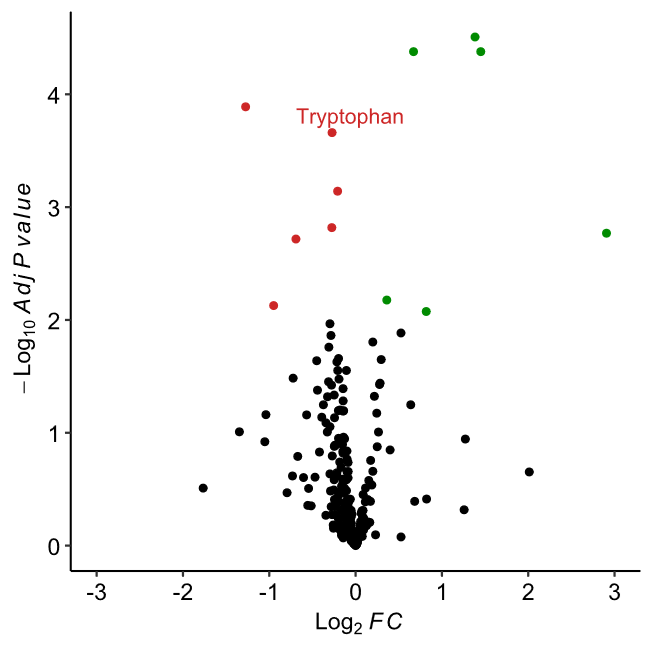


Figure. S2.

**Regorafenib-Avelumab treatment triggers Tryptophan consumption in cold soft-tissue sarcoma patients.** Volcano plot showcases the differential metabolites abundance in sarcoma patients upon

| **Patients characteristics** | **Safety population**  **(n=49)** |
| --- | --- |
| **Median age** (years, range) | 57 (21 – 81) |
| **Gender** |  |
| Female | 24 (49%) |
| Male | 25 (51%) |
| **Performance status ECOG** |  |
| 0 | 27 (55.1%) |
| 1 | 22 (44.9%) |
|  |  |
| **Tumor grade** |  |
| G1 | 8 (16.3%) |
| G2 | 11 (22.4%) |
| G3 | 11 (22.4%) |
| Not available | 19 (38.8%) |
| **Surgery of primary tumor** |  |
| Yes | 39 (79.6%) |
| No | 10 (20.4%) |
| **Neo-adjuvant chemotherapy** |  |
| Yes | 12 (24.5%) |
| No | 37 (75.5%) |
| **Adjuvant chemotherapy** |  |
| Yes | 16 (32.7%) |
| No | 33 (67.3%) |
| **Peri-operative radiotherapy** |  |
| Yes | 25 (51.0%) |
| No | 24 (49.0%) |
| **Histological subtypes** |  |
| Leiomyosarcoma (non uterine)  Leiomyosarcoma (uterine) | 11 (22.5%)  11 (22.5%) |
| Synovialosarcoma | 9 (18.4%) |
| Dedifferentiated Liposarcoma | 2 (4.1%) |
| Myxoid/round cell liposarcoma | 2 (4.1%) |
| UPS | 5 (10.2%) |
| Others* | 9 (18.3%) |
| **Metastatic sites** |  |
| Lung | 34 (72.3%) |
| Liver | 14 (29.8%) |
| Bone | 16 (34%) |
| Lymph node | 19 (40.4%) |
| **Previous chemotherapy treatment** |  |
| Anthracycline | 46 (93.9%) |
| Alkalating agent | 31 (63.3%) |
| Anti metabolite | 14 (28.6%) |
| Other chemotherapy | 26 (53.1%) |
| **Previous lines of treatment for advanced disease** |  |
| 1 | 18 (36.7%) |
| 2 | 12 (24.5%) |
| >2 | 19 (38.8%) |

Table S1. Baseline characteristics of the patients

|  | **Grade 1-2** | | **Grade 3-4** | |
| --- | --- | --- | --- | --- |
|  | **N** | **%** | **N** | **%** |
| Anemia | 6 | 12.2 | 1 | 2.0 |
| Hyperthyroidism | 3 | 6.1 | . | . |
| Hypothyroidism | 3 | 6.1 | . | . |
| Abdominal pain | 6 | 12.2 | . | . |
| Constipation | 6 | 12.2 | . | . |
| Diarrhea | 22 | 44.9 | . | . |
| Mucositis oral | 14 | 28.6 | . | . |
| Nausea | 11 | 22.4 | . | . |
| Vomiting | 5 | 10.2 | . | . |
| Fatigue | 36 | 73.5 | 5 | 10.2 |
| Fever | 13 | 26.5 | . | . |
| Infusion related reaction | 12 | 24.5 | 3 | 6.1 |
| Alanine aminotransferase increased | 6 | 12.2 | 1 | 2.2 |
| Alkaline phosphatase increase | 6 | 12.2 |  |  |
| Aspartate aminotransferase increase | 9 | 18.4 | 1 | 2.2 |
| Bilirubin increase | 12 | 24.5 | 2 | 4.1 |
| GGT increase | 1 | 2.2 | 1 | 2.0 |
| Thrombocytopenia | 8 | 16.3 | 2 | 4.0 |
| Weight loss | 7 | 14.3 | . | . |
| Anorexia | 19 | 38.8 | 2 | 4.1 |
| Hypophosphatemia | 9 | 18.4 | 1 | 2.0 |
| Arthralgia | 4 | 8.2 | 1 | 2.0 |
| Muscle cramp | 7 | 14.3 | 1 | 2.0 |
| Headache | 5 | 10.2 | . | . |
| Paresthesia | 1 | 2.0 | . | . |
| Dyspnea | 2 | 4.1 | . | . |
| Dysphonia | 12 | 24.5 | . | . |
| Dry skin | 5 | 10.2 | . | . |
| Palmar-plantar erythrodysesthesia syndrom | 25 | 51.0 | 6 | 12.2 |
| Pruritus | 2 | 4.1 | 1 | 2 |
| Skin and subcutaneous tissue disorders - Other, specify | 4 | 8.7 | . | . |
| Hypertension | 5 | 10.2 | 4 | 8.2 |

**Table S2. Treatment Related Adverse events (n=49)**
