# Supplementary material for: Reshaping the tumor microenvironment of cold soft-tissue sarcomas with anti-angiogenics: a phase 2 trial of regorafenib combined with avelumab
Source: Signal Transduct Target Ther. 2025 Jun 27;10:202. doi: 10.1038/s41392-025-02278-9 (PMC12205094; doi:10.1038/s41392-025-02278-9)
Supplement: Supplementary file 2 — Study Protocol [file 41392_2025_2278_MOESM2_ESM.pdf]

A PHASE I/II STUDY OF REGORAFENIB PLUS AVELUMAB IN SOLID TUMORS

Protocol *REGOMUNE*

VERSION N°8.0 DU |21|06|2023|- N° : IB 2017-01

Intégrant la modification substantielle n°1 du 28/02/2019
Intégrant la modification substantielle n°2 du 19/04/2019
Intégrant la modification substantielle n°3 du 24/09/2019
Intégrant la modification substantielle (Information-COVID-19)n°4 du 16/04/2020
Intégrant la modification substantielle (Information-COVID-19) n°5 du 04/06/2020
Intégrant la modification substantielle n°6 du 17/08/2020
Intégrant la modification substantielle (Information-Suspension inclusions) n°7 du 01/03/2021
Intégrant la modification substantielle (Information-Reprise partielle inclusions) n°8 du 27/04/2021
Intégrant la modification substantielle n°9 du 28/05/2021
Intégrant la modification substantielle n°10 du 05/10/2022
Intégrant la modification substantielle n°11 du 21/06/2023

EudraCT n° 2016-005175-27

CONFIDENTIAL

Coordinating Investigator

Sophie COUSIN, MD
Oncology

Department of Medical Oncology
Institut Bergonié
229, cours de l'Argonne – 33076 BORDEAUX Cedex, France
Tél : 05.56.33.78.05 – Fax : 05.56.33.04.85 – E-mail : s.cousin@bordeaux.unicancer.fr

Clinical Research Unit

Simone MATHOULIN-PÉLISSIER, MD, PhD — Institut Bergonié, Bordeaux
Head of the Unit

Carine BELLERA, PhD — Institut Bergonié, Bordeaux
Biostatistician

Sabrina SELLAN-ALBERT, MsC, — Institut Bergonié, Bordeaux
Head of project

David JUZANX, — Institut Bergonié, Bordeaux
Clinical Research Manager

Pharmacovigilance

UNICANCER — Paris

SPONSOR — **INSTITUT BERGONIE**

TABLE OF CONTENT

TABLE OF CONTENT 2
APPROVAL AND SIGNATURES OF PROTOCOL 6
SYNOPSIS 8
LIST OF ABBREVIATIONS AND DEFINITION OF TERMS 23
1. RATIONALE OF THE TRIAL 25
1.1. PATHOLOGIES OF INTEREST 25
1.1.1. Management of colorectal cancer in advanced setting (cohorts A – A') 25
1.1.2. Management of gastrointestinal stromal tumors in advanced setting (cohort B) 25
1.1.3. Management of advanced oesophageal and gastric carcinoma (cohort C) 25
1.1.4. Management of biliary tract cancer and hepatocellular carcinoma (cohort D) 26
1.1.5. Management of Soft Tissue Sarcoma (STS) (cohorts E – I) 26
1.1.6. Management of Radioiodine-Refractory Differentiated Thyroid Cancer (RR-DTC) (cohort F) 27
1.1.7. Management of Neuroendocrine gastroenteropancreatic tumors (GEP-NETs) (cohort G) 27
1.1.8. Management of non-small cell lung cancer (NSCLC) (cohort H) 27
1.1.9. Management of urothelial cancer (cohort J) 28
1.1.10. Management of HPV-associated cancer (cohort K) with molecular confirmation of p16 positive status 28
1.1.11. Management of triple negative breast cancer (cohort L) 28
1.1.12. Management of TMB-high solid tumors (cohort M) with TMB-high status already known 29
1.1.13. Management of MSI-high solid tumors (cohort N) with MSI-high status already known 29
1.1.14. Management of Non-clearcell renal carcinoma (cohort O) 29
1.1.15. Management of Malignant pleural mesothelioma (cohort P). 30
1.2. REGORAFENIB 30
1.2.1. Chemical structure 30
1.2.2. Preclinical data and mechanism of action 30
1.2.3. Clinical data 30
1.3. AVELUMAB 30
1.3.1. Chemical structure 30
1.3.2. Preclinical and mechanism of action 31
1.3.3. Clinical data 31
1.4. STUDY RATIONALE 31
1.5. BENEFIT/RISK AND ETHICAL ASSESSMENT 32
2. OBJECTIVES 32
2.1. PRIMARY OBJECTIVE 32
2.1.1. Phase I trial 32
2.1.2. Phase II trial 32
2.2. SECONDARY OBJECTIVES 33
2.2.1. Phase I trial 33
2.2.2. Phase II trials 33
3. STUDY DESIGN 33
3.1. OVERALL STUDY DESIGN 33
3.2. PHASE I – DOSE ESCALATION PART 34
3.2.1. Definitions 34
3.2.2. Treatment scheme 34
3.2.3. Dose levels 34
3.3. PHASE II TRIALS 35
3.4. PATIENT'S REPLACEMENT 36
4. SELECTION OF PATIENTS 36
4.1. INCLUSION CRITERIA 36
4.2. NON-INCLUSION CRITERIA 38
5. STUDY PLAN 39
5.1. DURATION OF STUDY (WHOLE POPULATION) 39
5.1.1. Phase I trial - Dose escalation part 39

CONFIDENTIAL

5.1.2. Phase II trials - Expansion cohorts ... 40
5.2. DEFINITIONS OF DURATION OF STUDY AND TREATMENT (PER PATIENT) ... 40
5.3. PROTOCOL DEVIATION ... 41
5.4. SCREENING EVALUATION ... 41
5.5. EVALUATIONS DURING TREATMENT ... 43
5.6. EVALUATION AT SAFETY FOLLOW-UP VISIT ... 44
5.7. FOLLOW-UP ... 44

6. REGISTRATION PROCEDURES ... 45

6.1. PHASE I TRIAL – ESCALATION PART ... 45
6.1.1. Screening ... 45
6.1.2. Inclusion ... 45
6.2. PHASE II TRIAL ... 45
6.2.1. Screening ... 45
6.2.2. Inclusion ... 46

7. STUDY TREATMENTS ... 46

7.1. DESCRIPTION OF TREATMENT ... 46
7.2. PHARMACEUTICAL INFORMATIONS ... 46
7.3. ADMINISTRATION OF TREATMENT ... 46
7.3.1. Regorafenib ... 47
7.3.2. Avelumab ... 47
7.4. RESTRICTION DURING THE STUDY ... 47
7.4.1. Contraception ... 47
7.4.2. Food intakes restriction ... 49
7.5. GENERAL CONCOMITANT MEDICATION ... 49
7.5.1. Acceptable concomitant medication ... 49
7.5.2. Prohibited concomitant medication ... 49
7.5.3. Potential drug interaction ... 50
7.6. DOSING DELAYS/DOSE MODIFICATIONS AND ADVERSE EVENT MANAGEMENT ... 51
7.6.1. Immune related adverse events (irAE) ... 51
7.6.2. Management of Adverse Events ... 51
7.7. PACKAGING AND LABELING ... 52
7.8. SUPPLIES AND DRUG ACCOUNTABILITY ... 52
7.9. TREATMENT COMPLIANCE ... 52

8. STUDY EVALUATIONS ... 52

8.1. CENTRAL REVIEW FOR DIAGNOSIS, IMMUNE SIGNATURE AND PROGRESSIVE DISEASE ... 53
8.1.1. Diagnosis ... 53
8.1.2. Immune signature - Central review before inclusion ... 53
8.1.3. Diagnosis of progressive disease – Central review before inclusion – cohorts E (STS), F (RR-DTC) and I (Solid tumors-TLS+) ... 54
8.2. EFFICACY – FOR PATIENTS INCLUDED IN PHASE II TRIALS ONLY ... 54
8.2.1. Assessing Objective Tumor Response (RECIST v1.1) ... 54
8.2.2. Centralized Radiological Review (Institut Bergonié) ... 54
8.3. SAFETY ... 55

9. STUDY ENDPOINTS ... 55

9.1. PHASE I TRIAL ... 55
9.1.1. Primary endpoint ... 55
9.1.2. Secondary endpoints ... 56
9.2. PHASE II TRIALS ... 56
9.2.1. Primary endpoint ... 56
9.2.2. Secondary endpoints ... 57

10. STATISTICAL CONSIDERATIONS ... 58

10.1. HYPOTHESES AND NUMBER OF SUBJECTS NEEDED ... 58
10.1.1. Phase I trial ... 58
10.1.2. Phase II trials: Cohorts A, B, C, D, E, F, G ... 58
10.1.3. Phase II trial: Cohort H (NSCLC) ... 59
10.1.4. Phase II trial: Cohort I (Solid tumors-TLS+) ... 59

CONFIDENTIAL

10.1.5. Phase II trial: Cohort A' (Colorectal @ 80 mg), with immune signature (based on low tumor-associated macrophages infiltrate level) *59*
10.1.6. Phase II trial : Cohorts J (Urothelial cancer) and L (Triple negative breast cancer) *60*
10.1.7. Phase II trials: Cohort K (HPV-associated cancer) *60*
10.1.8. Phase II trials: Cohorts M (TMB-high solid tumors) and O (Non clear-cell renal carcinoma) *60*
10.1.9. Phase II trials: Cohort N (MSI-high solid tumors) *60*
10.1.10. Phase II trials: Cohort P (Malignant pleural mesothelioma) *61*
10.2. Definition of study populations 61
10.2.1. Phase I (Dose escalation part) *61*
10.2.2. Phase II trials *61*
10.3. Statistical analysis 62
10.3.1. Patient characteristics at baseline *62*
10.3.2. Endpoint analyses *62*

11. ADVERSE EVENTS 64

11.1. Description of safety evaluation criteria 64
11.2. Definition 64
11.2.1. Adverse event *64*
11.2.2. Serious adverse event *64*
11.2.3. Non serious adverse event *65*
11.2.4. Adverse effect *65*
11.2.5. Expected/Unexpected character *65*
11.2.6. Intensity criterion *65*
11.2.7. New information *65*
11.2.8. Special considerations *65*
11.2.9. Causal relationship *66*
11.3. Serious adverse event and new information notification (responsibility of the investigator) 67
11.4. Events of special interest 68
11.5. Reporting pregnancy cases occurred within the clinical trial 68
11.6. Non serious adverse event 69
11.7. Responsibility of Vigilance Unit 70
11.8. Notification and registration of unexpected serious adverse events and new information (responsibility of the sponsor) 70

12. QUALITY ASSURANCE AND TRIAL MONITORING 70

12.1. Monitoring of the trial 70
12.1.1. Steering Committee *70*
12.1.2. Independent Data Monitoring Committee *70*
12.2. Quality assurance 71
12.2.1. Data collection *71*
12.2.2. Monitoring *71*
12.2.3. Handling of missing data *72*
12.2.4. Audits *72*
12.2.5. Data management *72*

13. REGULATORY ASPECTS AND ETHICAL CONSIDERATIONS 72

13.1. Clinical Trial authorization 73
13.2. Insurance policy 73
13.3. Informing and obtaining consent from patients 73
13.4. Sponsor's responsibilities 74
13.5. Investigators' responsibilities 74
13.6. Authority to execute the trial 75
13.7. Regulations governing the collection of human biological samples 75
13.8. Federation des Comites de Patients pour la Recherche Clinique en Cancerologie (FCPRCC) (Federation of Patient Committees for Clinical Research in Oncology) 75
13.9. Data processing 75

14. CONFIDENTIALITY AND OWNERSHIP OF DATA 76

15. PUBLICATION AND VALORISATION 76

15.1. Scientific communication 76

CONFIDENTIAL

15.2. INFORMATION TO PATIENTS 77
16. REFERENCES **78**
17. PHARMACOKINETIC, AND ANCILLARY STUDIES **82**
17.1. PHARMACOKINETIC STUDY 82
17.1.1. Collection of available specimens 82
17.1.2. PK analysis 82
17.1.3. Site performing PK study 82
17.1.4. Shipping of specimens 82
17.2. BIOMARKER STUDY 82
17.2.1. Collection of Specimen(s) 82
17.2.2. Rational 83
17.3. ANCILLARY STUDY 85
17.3.1. Collection of Specimen(s) 85
17.3.2. Handling and shipping of Specimen(s) 85
17.3.3. Ancillary analysis 85
APPENDIX 2: MDRD FORMULA **87**
APPENDIX 3: EVALUATION OF RESPONSE. THE RECIST **88**
APPENDIX 4: PATIENT MEDICATION DIARY **91**
APPENDIX 5: SERIOUS ADVERSE EVENT NOTIFICATION FORM **93**
APPENDIX 6: PREGNANCY NOTIFICATION FORM **96**
APPENDIX 7: DOSE MODIFICATION/DELAY FOR TOXICITIES RELATED TO REGORAFENIB (EXCEPT FOR HAND-FOOT SYNDROME REACTION, HYPERTENSION, AND ALT AND/OR AST AND/OR BILIRUBIN INCREASES) **98**
APPENDIX 8: DOSE DELAYS FOR TOXICITIES RELATED TO AVELUMAB AND REGORAFENIB INCLUDED IMMUNE-RELATED TOXICITIES **99**
APPENDIX 9: CONCOMITANT TREATMENTS **110**
APPENDIX 10: SUMMARY OF THE CHOI CRITERIA **111**

CONFIDENTIAL

APPROVAL AND SIGNATURES OF PROTOCOL

Title of protocol : A phase I/II study of Regorafenib plus Avelumab in digestive tumors

**Competent authority**	Name : ANSM	Initial authorization date :	06/03/2018
		Reference :	MEDAECNAT-2017-12-00010
		Authorization of MSA1	14/03/2019
		Authorization of MSI2	NA
		Authorization of MUS3	21/10/2019
		Authorization of MSI4	NA
		Authorization of MSI5	NA
		Authorization of MSA6	03/11/2020
		Authorization of MSI7	NA
		Authorization of MSI8	NA
		Authorization of MSA9	25/06/2021
		Authorization of MSA10	10/10/2022
		Authorization of MSA11	
**Ethic Committee**	Name : CPP Sud Est II	Initial approval date :	14/03/2018
		Référence :	2018-08-2
		Approval of MSA1	10/04/2019
		Approval of MSI2	22/05/2019
		Approval of MUS3	23/10/2019
		Approval of MSI4	NA
		Approval of MSI5	NA
		Approval of MSA6	18/11/2020
		Approval of MSI7	NA
		Approval of MSI8	NA
		Approval of MSA9	24/06/2021
		Approval of MSA10	05/10/2022
		Approval of MSA11	

Name and responsability	Address	Date	Signature
**Managing Director**	Pr François-Xavier MAHON<br>Institut Bergonié – Bordeaux<br>Tel:33(0)556333333 /<br>Fax:33(0)556333330<br>Mail : f.mahon@bordeaux.unicancer.fr	21/06/2023	
**Coordinating Investigator**	Dr Sophie COUSIN<br>Institut Bergonié – Bordeaux<br>Tel : 33(0)556337805 / Fax:<br>33(0)556330485<br>Mail : s.cousin@bordeaux.unicancer.fr	21/06/2023	
**Clinical Research Responsive**	Pr Simone MATHOULIN-PÉLISSIER<br>Institut Bergonié – Bordeaux<br>Tel : 33(0)556333398 / Fax :<br>33(0)56330466<br>Mail : s.mathoulin@bordeaux.unicancer.fr	21/06/2023	
**Biostatistician**	Carine BELLERA<br>Institut Bergonié – Bordeaux<br>Tel : 33(0)556330495 / Fax :<br>33(0)556330485<br>Mail : c.bellera@bordeaux.unicancer.fr	21/06/2023	
**Data Manager**	Sandrine Barbotin<br>Institut Bergonié – Bordeaux<br>Tel : 33(0)556334028/ Fax :<br>33(0)556330485<br>Mail : s.barbotin@bordeaux.unicancer.fr	21/06/2023	
**Head of project**	Sabrina SELLAN-ALBERT<br>Institut Bergonié – Bordeaux<br>Tél : 33(0)556337805 / Fax :<br>33(0)556330485	21/062023	

CONFIDENTIAL

	Mail : s.albert@bordeaux.unicancer.fr		
**Clinical Research Manager**	David JUZANX<br>Institut Bergonié – Bordeaux<br>Tel : 33(0)524071925 / Fax : 33(0)556330485<br>Mail : d.juzanx@bordeaux.unicancer.fr	21/06/2023	
**Pharmacist**	Ludivine POIGNIE<br>Institut Bergonié – Bordeaux<br>Tel : 33(0)556333394 / Fax : 33(0)556330485<br>Mail : l.poignie@bordeaux.unicancer.fr	21/06/2023	

I acknowledge having read the whole protocol, and I pledge to lead this protocol in accordance with the Good Clinical Practice (decision of 24 November 2006), the Public Health Law No. 2006-806 of August 09, 2004 and the implementing Decree n° 2006-477 of April 26, 2006 and as described in this document.

I assume my responsibilities as referent investigator including:
- Collection of informed consent, dated and signed by patients before any selection procedure in the protocol,
- Validation of case report forms, completed for each patient included in the study,
- Direct access to source documents for verification by the clinical research assistant (CRA) commissioned by the sponsor,
- Archiving of critical documents of the study for a 15 year-period.

Name and address of the investigating center:
Name of the Coordinating Investigator :
Date : |__|__| |__|__| |__|__|__|__| Signature :

CONFIDENTIAL

SYNOPSIS

**Title of the study**	**A phase I/II study of Regorafenib plus Avelumab in solid tumors**
**Abbreviation of the trial**	**REGOMUNE**
**Sponsor Identification**	**Institut Bergonié, Regional Comprehensive Cancer Center**
**Coordinating Investigator**	**Doctor Sophie Cousin**<br>**Department of Medical Oncology**
**Number of investigational sites planned**	Phase I, 1 center: Institut Bergonié, Bordeaux<br><br>Phase II, 7 centers:<br>- Institut Bergonié, Bordeaux<br>- IUCT, Toulouse<br>- ICM, Montpellier<br>- CLB, Lyon<br>- IGR, Villejuif<br>- CHU, Brest<br>- Institut Curie, Paris (sites de Curie et de Saint-Cloud)
**Number of patients**	Phase I (escalation part): 6-12 patients<br>Phase II (expansion cohorts): 735 patients
**Duration of the study**	Planned enrollment period: 72 months (+24 months)<br>Treatment duration: until progression<br>Follow-up: 12 months<br>Study period: 84 months (+24 months)
**Medical conditions**	Adult patients with advanced or metastatic solid tumors
**Objectives**	**PHASE I TRIAL/ DOSE ESCALATION**<br><br>**Primary objective**<br>Primary objective of the phase I trial is to establish the recommended phase II dose (RP2D), the maximum tolerated dose (MTD) evaluated on the first cycle (D1 to D28), the safety profile, and the dose limiting toxicities (DLT) of Regorafenib when prescribed in association with Avelumab (no dose escalation for Avelumab) in patients treated for advanced digestive solid tumors.<br><br>**Secondary objectives**<br>• To evaluate the preliminary signs of anti-tumor activity of Regorafenib when prescribed in association with Avelumab in terms of 6-month objective response, 6-month progression-free status, best overall response, objective response under treatment, growth modulation index (GMI), 1-year progression-free survival (PFS) and 1 year overall survival (OS).<br>• To describe the pharmacokinetics (PK) of Regorafenib when prescribed in association with Avelumab.<br>• Biomarker study: To perform pharmacodynamic (PD)/mechanism of action (MOA) biomarkers analysis as well as predictive biomarkers analysis (levels of angiogenic and immunologic biomarkers in blood/tissue at baseline and different study time points).<br><br>**PHASE 2 TRIALS**<br><br>**Primary objective**<br>To investigate the antitumor activity of Regorafenib when prescribed in association with Avelumab, independently for 17 cohorts of patients : Colorectal cancer not MSI-H or MMR-deficient (Cohorts A and A' with immune signature (based on low tumor-

CONFIDENTIAL

	associated macrophages infiltrate level), GIST (Cohort B), Oesophageal or gastric carcinoma (Cohort C), Biliary tract cancer, hepatocellular carcinoma (Cohort D), Soft Tissue Sarcoma (STS – Cohort E), Radioiodine-Refractory Differentiated Thyroid Cancer (RR-DTC – Cohort F), Neuroendocrine gastroenteropancreatic tumors (GEP-NETs – Cohort G), Non-small cell lung cancer (NSCLC - Cohort H), Solid tumors (including Soft Tissue Sarcoma) with immune signature (TLS+) (Cohort I), urothelial cancer (Cohort J), HPV-associated cancer (Cohort K), triple negative breast cancer (L), TMB-high solid tumors (Cohort M), MSI-high solid tumors (Cohort N), non clear-cell renal carcinoma (Cohort O) and malignant pleural mesothelioma (Cohort P).<br><br>For cohorts A (Colorectal cancer not MSI-H or MMR-deficient [standard dose]), C (Oesophageal or gastric carcinoma), D (Biliary tract cancer, hepatocellular carcinoma), E (Soft Tissue Sarcoma [STS]), F (Radioiodine-Refractory Differentiated Thyroid Cancer), G (Neuroendocrine gastroenteropancreatic tumors):: antitumoral activity will be assessed in terms of objective response under treatment based on adapted RECIST 1.1 criteria after in-stream centralized radiological review (see endpoints section).<br><br>For cohorts B (GIST), H (NSCLC), I (Solid tumors -TLS+), M (TMB-high solid tumors), N (MSI-high solid tumors), O (non clear-cell renal carcinoma) and P (malignant pleural mesothelioma), antitumor activity will be assessed in terms of 6-month progression-free rate (6-month PFR) based on RECIST 1.1 criteria after in-stream centralized radiological review (see endpoints, section 9).<br><br>For cohort A' with immune signature (based on low tumor-associated macrophages infiltrate level, antitumor activity will be assessed in terms of 4-month progression-free rate (4-month PFR) based on RECIST 1.1 criteria after in-stream centralized radiological review (see endpoints, section 9).<br><br>For cohorts J, K and L, antitumor activity will be assessed in terms of disease control rate at 6-month (6-month DCR) based on RECIST 1.1 criteria after in-stream centralized radiological review (see endpoints, section 9).<br><br>**<u>Secondary objectives (for each phase II trial)</u>**<br><br>• To evaluate the antitumor activity of Regorafenib when prescribed in association with Avelumab in terms of 6-month objective response, 6-month progression-free rate, best overall response, growth modulation index (GMI), 1-year progression-free survival (PFS) and 1-year overall survival (OS).<br>• To evaluate the Regorafenib safety profile when prescribed in association with Avelumab.<br>• To perform pharmacodynamic (PD)/mechanism of action (MOA) biomarkers analysis as well as predictive biomarkers analysis (levels of angiogenic and immunologic biomarkers in blood/tissue at baseline and different study time points).<br>• For the cohort B: To evaluate the efficacy based on Choi criteria by independent radiologic review
**Study design**	**<u>STUDY DESIGN</u>**<br>This is a multicenter, prospective open-labeled phase Ib trial based on a dose escalation study design (3+3 traditional design) assessing three dose levels of Regorafenib given in combination with Avelumab (no dose escalation for Avelumab) in patients with advanced digestive solid tumors, followed by independent phase II trials in patients with advanced or metastatic solid tumors to evaluate the association of Regorafenib at the RP2D with Avelumab in 17 cohorts:<br>• Cohort A: Colorectal cancer not MSI-H or MMR-deficient (standard dose)<br>• Cohort B: GIST<br>• Cohort C: Oesophageal or gastric carcinoma<br>• Cohort D: Biliary tract cancer, hepatocellular carcinoma<br>• Cohort E : Soft Tissue Sarcoma (STS)<br>• Cohort F : Radioiodine-Refractory Differentiated Thyroid Cancer (RR-DTC)<br>• Cohort G : Neuroendocrine gastroenteropancreatic tumors (GEP-NETs)

- Cohort H: Non-small cell lung cancer (NSCLC)
- Cohort I: Solid tumors (including Soft Tissue Sarcoma) with immune signature (TLS+)
- Cohort J: Urothelial cancer
- Cohort K: HPV-associated cancer
- Cohort L: Triple negative breast cancer
- Cohort M: TMB-high solid tumors
- Cohort N: MSI-high solid tumors
- Cohort O: Non clear-cell renal carcinoma
- Cohort P: Malignant pleural mesothelioma.

Moreover, we propose to evaluate in a phase II trial, the association of a low-dose of regorafenib (80 mg/day) with avelumab in patients with colorectal cancer not MSI-H or MMR-deficient (cohort A') with immune signature (based on low tumor-associated macrophages infiltrate level.

- Cohort A': Colorectal cancer not MSI-H or MMR-deficient (low dose) with immune signature (based on low tumor-associated macrophages infiltrate level.

<u>DEFINITIONS</u>

Dose-limiting toxicity (DLT) is defined as an adverse event or laboratory abnormality that fulfills all the criteria below:

- Begins on the first 28 days of treatment
- Is considered to be at least possibly related to the study treatment
- Meets one of the criteria below:
    - Hematotoxicity:
        - Persistent grade 4 neutropenia lasting ≥7 days;
        - When a neutrophil count of <1000/mm3 and fever of ≥38.0°C is observed for >2 days;
        - Grade 4 thrombopenia or thrombopenia associated with a hemorrhage requiring platelet transfusion.
        - Grade 3 thrombocytopenia with bleeding.
    - Non-hematotoxicity:
        - ≥Grade 3 non-hematotoxicities are considered as DLTs with the following specifications:
        - ≥Grade 3 diarrhea, nausea, vomiting, and loss of appetite for ≥5 consecutive days (despite supportive therapy);
        - ≥Grade 3 electrolyte imbalance for ≥7 consecutive days (despite supportive therapy);
        - Grade 3 dermatologic toxicity hand and foot syndrome and non-life threatening events) for ≥7 consecutive days;
        - Grade 4 dermatologic toxicity of any duration;
        - ALT/AST increases 5-8 X ULN with concomitant bilirubin increase < 2 X ULN not resolving to < 5 X ULN within 7 days
        - ALT/AST increases 5-8 X ULN with concomitant bilirubin increase > 2 X ULN
        - ALT/AST increases > 8 X ULN regardless of concomitant bilirubin increase
        - Bilirubin increase > 3 X ULN not resolving to baseline within 7 days
        - ≥Grade 3 immune-related adverse events lasting for ≥8 consecutive days despite appropriate therapy including steroids and/or immunosuppressive agents

In addition, the following will be considered as DLT:

- Any other study drug related AE considered significant enough to be qualified as DLT in the opinion of the investigators after discussion with the sponsor
- Any drug-related AE leading to Regorafenib or Avelumab relative dose intensity lower or equal to 75% over the first treatment cycle.

ONFIDENTIAL

Maximum tolerated dose (MTD): is defined as the highest dose at which no more than 1 in 6 of the patients in the cohort experienced a DLT in the first treatment cycle.

Recommended phase II dose (RP2D): will be identified by the steering committee based on the MTD, additional safety data (all cycles), PK data and PD data. Data from all cycles will be used to define the dose level to be recommended for further investigations in phase II

PHASE 1: DOSE ESCALATION

- 3 dose levels of Regorafenib will be investigated in combination with Avelumab:

Level	-1	1	2
Avelumab	*10mg/kg*	*10mg/kg*	*10mg/kg*
Regorafenib	80 mg	120 mg	160 mg

- The starting dose of Regorafenib is 120 mg (dose level 1).
- The maximum dose of Regorafenib administered (160 mg) will not be exceeded.
- No skipping of the dose will be allowed.
- For a given patient, dose will never be escalated.
- Patients will be allocated to the 3 dose levels following a 3 + 3 design.
- A minimum of 3 patients and a maximum of 6 patients will be entered on each dose level.
- All 3 patients within a dose level will be observed during 28 days (the period of observation of DLTs) before accrual to the next higher dose level may begin.
- Dose escalation will proceed according to the following scheme:

Number of patients with DLT at one dose level	Escalation Decision Rule
0 out of 3	Enter 3 patients at the next dose level.
≥2	Dose escalation will be stopped. This dose level will be declared as the maximum administered dose (MAD). Three additional patients will be entered at the next lowest dose level if only 3 patients were treated previously at that dose.
1 out of 3	Enter at least 3 more patients at this dose level.<br>• If 0 of these 3 patients experience DLT, proceed to the next dose level.<br>• If 1 or more of this group suffer DLT, dose escalation will be stopped, and this dose is declared as the MAD. Three additional patients will be entered at the next lowest dose if only 3 patients were treated previously at that dose.
≤1 out of 6	If this is the highest dose level, this will be the maximum tolerated dose (MTD).<br>Else, proceed to the next dose level.

- As described above, the maximum administered dose (MAD) for Regorafenib is the dose in which ≥ 2/3 or ≥ 2/6 patients experience DLT.
- If the MAD for Regorafenib is seen at the starting dose level, then dose level "-1" will be the recommended dose. (Note: upon dose reduction, MAD criteria could be fulfilled but no tolerable combination may be found to continue into Phase II).
- The MTD for Regorafenib is defined as the highest dose at which no more than 1 in 6 of the patients in the cohort experienced a DLT during the period of observation of DLTs.

CONFIDENTIAL

	• The steering committee will meet before proceeding or not to each dose escalation. In addition, the steering committee will be consulted to resolve any specific issue regarding the DLT status.<br>• The sponsor may decide to submit the conclusions of the steering committee for the definition of MTD and RP2D for approval to an independent data monitoring committee (IDMC) before opening the phase II trials.<br><br>**PHASE 2 TRIALS**<br>Following the dose escalation trial, the RP2D for Regorafenib will be identified by the steering committee based on all safety data from patients included in the dose escalation part, as well as PD data. Once the MTD and RP2D have been defined, the single-arm open-label phase II trials will be opened:<br>• Cohort A: Colorectal cancer not MSI-H or MMR-deficient (standard dose)<br>• Cohort B: GIST<br>• Cohort C: Oesophageal or gastric carcinoma<br>• Cohort D: Biliary tract cancer, hepatocellular carcinoma<br>• Cohort E : Soft Tissue Sarcoma (STS)<br>• Cohort F : Radioiodine-Refractory Differentiated Thyroid Cancer (RR-DTC)<br>• Cohort G : Neuroendocrine gastroenteropancreatic tumors (GEP-NETs)<br>The design of these single-arm phase II trials will follow an adaptative trial design (Bayesian approach).<br>All patients will be treated at the RP2D of Regorafenib (as defined in the dose escalation trial) given in association with Avelumab with the same schedule as in the dose escalation trial.<br><br>• Cohort H: Non-small cell lung cancer (NSCLC)<br>• Cohort I: Solid tumors (including Soft Tissue Sarcoma) with immune signature (TLS+)<br>The design of these single-arm phase II trials will follow an optimal two-stage Simon's design.<br>The administered dose of Regorafenib will be the RP2D defined in the dose escalation part of the trial.<br><br>• Cohort A': Colorectal cancer not MSI-H or MMR-deficient with immune signature (based on low tumor-associated macrophages infiltrate.<br>The design of this single-arm phase II trial will follow an optimal two-stage Simon's design.<br>The administered dose of Regorafenib will be at a fixed low-dose of 80 mg/day.<br><br>• Cohort J: Urothelial cancer<br>• Cohort K: HPV-associated cancer<br>• Cohort L: Triple negative breast cancer<br>• Cohort M: TMB-high solid tumors<br>• Cohort N: MSI-high solid tumors<br>• Cohort O: Non clear-cell renal carcinoma<br>• Cohort P: Malignant pleural mesothelioma.<br>The design of these single-arm phase II trials will follow an exact single-stage A'Hern design.<br>The administered dose of Regorafenib will be the RP2D defined in the dose escalation part of the trial.
**Translational research**	For consenting patients during the phase I, optional biopsy at baseline and after 4 weeks of treatment will be performed for mechanisms of action comprehensive documentation: tumor VEGFR, PDGFR, HIF1alpha expression as well as lymphocytes, tumor-associated macrophages (TAM) and myeloid-derived suppressor cells (MDSC) tumor infiltrates (by immunohistochemistry).<br><br>For consenting patients during the phase II :<br>- Optional biopsy at baseline and after 4 weeks of treatment will be performed for mechanisms of action comprehensive documentation: tumor VEGFR, PDGFR, HIF1alpha expression as well as lymphocytes, tumor-associated macrophages

CONFIDENTIAL

	(TAM) and myeloid-derived suppressor cells (MDSC) tumor infiltrates (by immunohistochemistry).<br>- Optional stool sample at C1D1(pre-dose) for the microbiota
**Inclusion criteria**	1. Histology:<br>- Dose escalation part: histologically confirmed non MSI-H or MMR-deficient colorectal cancer, or GIST, or oesophageal or gastric carcinoma or hepatobiliary cancers,<br>- Phase II trials : histologically confirmed :<br>o non MSI-H or MMR-deficient colorectal cancer (cohort A)<br>o non MSI-H or MMR-deficient colorectal cancer with immune signature (cohort A'), i.e. low tumor-associated macrophages infiltrate level as determined by central review.<br>Except if the low level of tumor-associated macrophages infiltrate level has been already confirmed by Biopathological platform at Bergonié Institute, the low level of tumor-associated macrophages infiltrate level should be confirmed by central review based on FFPE (Formalin-Fixed Paraffin-Embedded) tumor tissue sample (archived or newly obtained by biopsy for research purpose). Note that the level of tumor-associated macrophages infiltrate could be determined by central analysis if not available before.<br>o or GIST (cohort B) : as recommended by INCa, patients must have diagnosis histologically confirmed by central review, except if it has been already confirmed by the RRePS Network.<br>o or oesophageal or gastric carcinoma (cohort C)<br>o or hepatobiliary cancers (cohort D)<br>o or Soft Tissue Sarcoma (STS) (cohort E) : as recommended by INCa, patients must have diagnosis histologically confirmed by central review, except if it has been already confirmed by the RRePS Network<br>o or Radioiodine-Refractory Differentiated Thyroid Cancer (RR-DTC) (cohort F)<br>o or Neuroendocrine gastroenteropancreatic tumors (GEP-NETs) grade 2 and 3 (cohort G),<br>o or Non-small cell lung cancer (cohort H),<br>o or Solid tumors including soft-tissue sarcoma with immune signature (cohort I), i.e. presence of mature tertiary lymphoid structures (TLS).<br>Except if presence of TLS have been already confirmed by Biopathological platform at Bergonié Institute, presence of TLS should be confirmed by central review based on FFPE (Formalin-Fixed Paraffin-Embedded) tumor tissue sample (archived or newly obtained by biopsy for research purpose). Note that the presence of TLS could be determined by central analysis if not available before.<br>o or urothelial cancer (cohort J)<br>o or HPV-associated cancer (cohort K) with molecular confirmation of p16 positive status.<br>o or triple negative breast cancer (cohort L)<br>o or TMB-high solid tumors (cohort M) with TMB-high status already known<br>o or MSI-high solid tumors (cohort N) with MSI-high status already known<br>o or Non clear-cell renal carcinoma (cohort O)<br>o or Malignant pleural mesothelioma (cohort P).<br><br>2. Advanced non resectable / metastatic disease<br>3. Patients for which either there is no further established therapy that is known to provide clinical benefit,<br>4. Age ≥ 18 years,<br>5. ECOG, Performance status ≤ 1,

6. Measurable disease according to RECIST v1.1,

7. Life expectancy > 3 months,

8. Except for cohorts F (RR-DTC), ≥ 1 previous line (s) of systemic therapy,

9. Adequate hematological, renal, metabolic and hepatic functions:

   a. Hemoglobin ≥ 9 g/dl (patients may have received prior red blood cell [RBC] transfusion, if clinically indicated); absolute neutrophil count (ANC) ≥ 1.5 x 10^9/l and platelet count ≥ 100 x 10^9/l.

   b. Alkaline phosphatase (AP), alanine aminotransferase (ALT) and aspartate aminotransferase (ASP) ≤ 2.5 x upper limit of normality (ULN) (≤ 5 in case of extensive skeletal involvement and/or liver metastasis for AP and ≤ 5 x ULN in case of liver metastasis for AST and ALT).

   c. Total bilirubin ≤ 1.5 x ULN.

   d.Albumin ≥ 25g/l.

   e. Calculated creatinine clearance (CrCl) ≥ 30 ml/min (according to Cockroft and Gault formula).

   f. Creatine phosphokinase (CPK) ≤ 2.5 x ULN

   g. INR or PT ≤ 1.5 x ULN

   h. aPTT ≤ 1.5 X ULN.

   i. Lipase ≤ 1.5 X ULN

   j. Cohort specific criteria: Patients with hepatocellular carcinoma must have a correct hepatocellular function, id est Child-Pugh A.

10. No prior or concurrent malignant disease diagnosed or treated in the last 2 years except for adequately treated in situ carcinoma of the cervix, basal or squamous skin cell carcinoma, or in situ transitional bladder cell carcinoma,

11. At least three weeks since last chemotherapy, immunotherapy or any other pharmacological treatment and/or radiotherapy,

12. Recovery to grade ≤ 1 from any adverse event (AE) derived from previous treatment, excluding alopecia of any grade and non-painful peripheral neuropathy grade ≤ 2 (according to the National Cancer Institute Common Terminology Criteria for Adverse Event (NCI-CTCAE, version 5.0)),

13. Women of childbearing potential must have a negative serum pregnancy test within 72 hours prior to receiving the first dose of study medication.

14. Both women and men must agree to use an highly effective method of contraception throughout the treatment period and for seven months (210 days) in WOCBP or four months (120 days) in men sexually active with WOCBP after discontinuation of treatment. Acceptable methods for contraception are described in section 7.4.1.

15. Voluntary signed and dated written informed consents prior to any specific study procedure,

16. Patients with a social security in compliance with the French law.

17. Documented disease progression (as per RECIST v1.1) before study entry.

   - For patient of cohorts E (STS) and cohort I (Solid tumors – TLS+) : this progression will be confirmed by central review on the basis of two CT scan or MRI obtained at less than 6 months in the period of 12 months prior to inclusion.
   - For patient of cohort F (RR-DTC) : this progression will be confirmed by central review on the basis of two CT scan or MRI obtained at less than 12 months prior to inclusion.

18. *Cancelled (MSA6)*

19. For patients with non-small cell lung cancer (cohort H):

   - Subjects with histologically or cytologically confirmed diagnosis of non-squamous NSCLC
   - Documented disease progression based on radiographic imaging, during or after a maximum of 2 lines of systemic treatment for locally/regionally advanced recurrent, Stage IIIb/Stage IV or metastatic disease. Two

CONFIDENTIAL

	components of treatment must have been received in the same line or as separate lines of therapy<br>• A maximum of 1 line of platinum-containing chemotherapy regimen in the metastatic setting, and<br>• A maximum of 1 line of PD(L)1 mAb containing regimen, and<br>• Patients must have received at least 4 months of PD(L1) mAb treatment.<br>o No EGFR, ALK, ROS1 positive tumor mutations<br>o Subjects with known BRAF molecular alterations must have had disease progression after receiving the locally available SoC treatment for the molecular alteration.<br>20. For patients with urothelial cancer (cohort J):<br>o A maximum of 1 line of PD(L)1 mAb containing regimen, and<br>o Patients must have received at least 4 months of PD(L1) mAb treatment.<br>21. For HPV-associated cancer (cohort K), TMB-high solid tumors (cohort M) MSI-high solid tumors (cohort N), Non clear-cell renal carcinoma (cohort O):<br>o A maximum of 1 line of PD(L)1 mAb containing regimen, and<br>o Patients must have received at least 4 months of PD(L1) in the case they received this treatment<br>22. For malignant pleural mesothelioma (Cohort P):<br>o A maximum of 1 line of PD(L)1/CTLA-4 mAb containing regimen, and<br>o Patients must have received at least 4 months of PD(L1)/CTLA-4 mAb treatment in the case they received this treatment<br>23. For triple-negative breast cancer patients (Cohort L)<br>o A maximum of 1 line of PD(L)1 mAb containing regimen, and<br>o Patients must have received at least 4 months of PD(L1) mAb treatment<br>Except if CPS<10, an anterior line of PD(L)1 mAb is not mandatory<br>24. For TMB-High cancer patients (Cohort M):<br>o TMB-High is defined as TMB score ≥ 16 mutations /megabase on tissue or blood sample
**Non Inclusion criteria**	1. Previous treatment with Avelumab or Regorafenib,<br>2. For cohorts A to G and A': Has received prior therapy with an anti-PD-1, anti-PD-L1, anti-PD-L2, anti-CD137, or anti-Cytotoxic T-lymphocyte-associated antigen-4 (CTLA-4) antibody (including ipilimumab or any other antibody or drug specifically targeting T-cell co-stimulation or checkpoint pathways),<br>3. Evidence of progressive or symptomatic or newly diagnosed central nervous system (CNS) or leptomeningeal metastases. Participants with previously treated brain metastases may participate provided they are stable (without evidence of progression by imaging for at least 4 weeks before the first dose of study treatment and any neurologic symptoms have returned to baseline), have no evidence of new or enlarging brain metastases confirmed by repeat imaging, and have not required steroids for at least 7 days before study treatment,<br>4. Men or women of childbearing potential who are not using an effective method of contraception as previously described;,<br>5. Participation to a study involving a medical or therapeutic intervention in the last 30 days,<br>6. Previous enrolment in the present study,<br>7. Patient unable to follow and comply with the study procedures because of any geographical, familial, social or psychological reasons,<br>8. Known hypersensitivity to any involved study drug or of its formulation components,<br>9. Active autoimmune disease that might deteriorate when receiving an immunostimulatory agent:<br>a. Subjects with diabetes type I, vitiligo, psoriasis, hypo- or hyperthyroid disease not requiring immunosuppressive treatment are eligible

b. Subjects requiring hormone replacement with corticosteroids are eligible if the steroids are administered only for the purpose of hormonal replacement and at doses ≤ 10 mg or 10 mg equivalent prednisone per day
c. Administration of steroids through a route known to result in a minimal systemic exposure (topical, intranasal, intro-ocular, or inhalation) are acceptable

10. Has a diagnosis of immunodeficiency or is receiving systemic steroid therapy or any other form of immunosuppressive therapy within 7 days prior to the first dose of trial treatment,
11. History of idiopathic pulmonary fibrosis (including pneumonitis), drug-induced pneumonitis, organizing pneumonia, or evidence of active pneumonitis on screening chest CT scan or interstitial lung disease with ongoing signs and symptoms at inclusion. History of radiation pneumonitis in the radiation field (fibrosis) is permitted,
12. Has known hepatitis B or hepatitis C, active and/or treated by antiviral therapy,
13. Has a known history of Human Immunodeficiency Virus (HIV) (HIV1/2 antibodies) or known acquired immunodeficiency syndrome (AIDS)
14. Spot urine must not show 1+ or more protein in urine or the patient will require a repeat urine analysis. If repeat urinalysis shows 1+ protein or more, a 24-hour urine collection will be required and must show total protein excretion <1000 mg/24 hours),
15. Major surgical procedure or significant traumatic injury within 28 days before start of study medication,
16. Non-healing wound, non-healing ulcer, or non-healing bone fracture requiring orthopedic treatment,
17. Patients with evidence or history of any bleeding diathesis, irrespective of severity,
18. Any hemorrhage or bleeding event ≥ CTCAE Grade 3 within 4 weeks prior to the start of study medication,
19. Arterial or venous thrombotic or embolic events such as cerebrovascular accident (including transient ischemic attacks), deep vein thrombosis or pulmonary embolism within 6 months before the start of study medication (except for adequately treated catheter-related venous thrombosis occurring more than one month before the start of study medication),
20. Ongoing infection > Grade 2 as per NCI CTCAE v5.0,
21. Uncontrolled hypertension (Systolic blood pressure > 140 mmHg or diastolic pressure > 90 mmHg) despite optimal medical management,
22. Congestive heart failure ≥ New York Heart Association (NHYA) class 2,
23. Unstable angina (angina symptoms at rest), new-onset angina (begun within the last 3 months),
24. Myocardial infarction less than 6 months before start of study drug
25. Uncontrolled cardiac arrhythmias,
26. Pregnant or breast-feeding patients
27. Individuals deprived of liberty or placed under legal guardianship,
28. Prior organ transplantation, including allogeneic stem-cell transplantation,
29. Known alcohol or drug abuse
30. Vaccination within 4 weeks of the first dose of Avelumab and while on trial is prohibited except for administration of inactivated vaccines.
31. Patients with any condition that impairs their ability to swallow and retain tablets,
32. Other severe acute or chronic medical conditions including immune colitis, inflammatory bowel disease, immune pneumonitis, pulmonary fibrosis or psychiatric conditions including recent (within the past year) or active suicidal ideation or behavior; or laboratory abnormalities that may increase the risk associated with study participation or study treatment administration or may interfere with the interpretation of study results and, in the judgment of the investigator, would make the patient inappropriate for entry into this study..
33. Patient with anti-Vitamine K therapy,
34. Suspected or known intraabdominal fistula,
35. For cohort H (NSCLC):
    - Received > 2 prior lines of therapy for NSCLC, including subjects with BRAF molecular alterations,

- Subjects with known EGFR/ALK/ROS1 molecular alterations are excluded from participation in this study.

Route of administration

Avelumab will be administrated by intravenous 1-hour infusion every 2 weeks starting at Cycle 1 Day 15. Treatment will be administered during day-hospitalization.

Regorafenib will be taken orally once daily for three weeks on/ one week off. Treatment will be dispensed every month by the hospital’s pharmacy.

Treatment schedule

Phase I:

Regimen description				
**Agent**	**Dose**	**Route**	**Schedule**	**Cycle length**
Avelumab	10mg/kg	IV	Every 2 weeks (start on Cycle 1 Day 15)	4 weeks
Regorafenib	As appropriate for assigned dose level	Per os	Continuous, once daily<br>Day 1 – Day 21	

<u>3 dose levels</u>:

**Level**	**-1**	**1**	**2**
Avelumab	*10mg/kg*	*10mg/kg*	*10mg/kg*
Regorafenib	80 mg	120 mg	160 mg

Phase II:

For cohort A to I, as well as J to P: Patients will receive the same treatment administration modalities as those for the phase 1 trial with Regorafenib prescribed at the RP2D.
For cohort A’: Patients will receive the same treatment administration modalities as those for the phase 1 trial with Regorafenib prescribed at a fixed dose of 80 mg/day.

Endpoints

<u>PHASE I TRIAL/ DOSE ESCALATION</u>

Primary endpoint

- Toxicity graded using the common toxicity criteria from the NC-CTCAE v5
- Incidence rate of DLT at each dose level during the first 28 days.

Secondary endpoints

- Preliminary signs of antitumor activity in terms of:
  - Best overall response defined as the best response recorded from the start of the study treatment until the end of treatment taking into account any requirement for confirmation as per RECIST v1.1 criteria.
  - Objective response rate (ORR) defined as the proportion of patients with complete response or partial response, as per RECIST 1. ORR under treatment and 6-month ORR will be reported.
  - Progression-free rate (PFR) at 6 months defined as the proportion of patients with complete response, partial response or stable disease more than 24 weeks as per RECIST v1.1 criteria.
  - Progression-free survival (PFS) defined as the time from study treatment initiation to the first occurrence of disease progression or death (of any cause), whichever occurs first. 1-year PFS rate and median PFS will be reported.
  - Overall Survival (OS) defined as the time from study treatment initiation to death (of any cause). 1-year OS rate and median OS will be reported.
  - Growth modulation index (GMI): GMI will be defined for each patient as the ratio of its PFS on Regorafenib + Avelumab treatment to its PFS on the previous line of therapy. This method accounts for inter-patient variability, the patient serving as his/her own control and implies by the

CONFIDENTIAL

natural history of the disease that the PFS tends to become shorter in successive lines of therapy. It is thought that an anti-cancer agent should be considered effective if the GMI is greater than 1.3

- PK measurements expressed as AUC, half-life and concentration peak for Regorafenib
- Pharmacodynamic activity: Predictive biomarkers analysis and pharmacodynamic (PD)/mechanism of action (MOA) in blood (levels of angiogenic and immunologic biomarkers in blood at baseline and different study time points), potentially including but not limited to:
    - Serum/plasma cytokines levels (ELISA)
    - Treg, CD4+, CD8+ and DR lymphocytes subpopulations (flow cytometry)
    - Archived tumor tissue will be collected for assessment of tumor VEGFR, PDGFR, HIF1alpha expression and lymphocytes, TAM and MDSC tumor infiltrates (IHC)
    - In additional, for all patients, optional biopsy at baseline and after 4 weeks of treatment will be proposed for mechanisms of action documentation: tumor VEGFR, PDGFR, HIF1alpha expression as well as PD-L1/PD1, lymphocytes, TAM, MDSC tumor infiltrates (IHC) and mutational burden.

<u>PHASE II TRIALS</u>

Primary endpoint

- **For cohorts A (Colorectal cancer not MSI-H or MMR-deficient [standard dose]), C (Oesophageal or gastric carcinoma), D (Biliary tract cancer, hepatocellular carcinoma), E (Soft Tissue Sarcoma [STS]), F (Radioiodine-Refractory Differentiated Thyroid Cancer), G (Neuroendocrine gastroenteropancreatic tumors):** Antitumor activity will be assessed in terms of objective response under treatment based on adapted RECIST 1.1 criteria, and after in-stream centralized radiological review:
    - Objective response under treatment is defined as patients with confirmed complete response (CR) or partial response, as per RECIST v1.1 criteria, observed during treatment with the investigational product(s).
    - As per RECIST v1.1 criteria, to be considered as “confirmed”, complete and partial responses will have to be confirmed at least 4 weeks later to ensure responses identified are not the result of measurement errors. If the new imaging to confirm response is not performed after 4 weeks, complete or partial responses will be considered as unconfirmed responses.
    - Objective response rate (ORR) under treatment is defined as the proportion of patients with objective response (confirmed or unconfirmed) under treatment based on adapted RECIST 1.1.
- **For cohorts B (GIST), H (NSCLC), I (Solid tumors -TLS+), M (TMB-high solid tumors), N (MSI-high solid tumors), O (non clear-cell renal carcinoma) and P (malignant pleural mesothelioma):** antitumor activity will be assessed in terms of 6-month progression-free rate (6-month PFR) based on RECIST 1.1 criteria after in-stream centralized radiological review. 6-month PFR is defined as the proportion of patients with progression-free status at 6 months. Progression-free status is defined as complete response (confirmed or unconfirmed), partial response (confirmed or unconfirmed) or stable disease more than 24 weeks as defined as per RECIST v1.1 criteria.
- **For cohort A’ with immune signature (based on low tumor-associated macrophages infiltrate level):** antitumor activity will be assessed in terms of 4-month progression-free rate (4-month PFR) based on RECIST 1.1 criteria after in-stream centralized radiological review. 4-month PFR is defined as the proportion of patients with progression-free status at 4 months. Progression-free status is defined as complete response (confirmed or unconfirmed), partial

CONFIDENTIAL

response (confirmed or unconfirmed) or stable disease more than 24 weeks as defined as per RECIST v1.1 criteria.

- **For cohorts J (Urothelial cancer), K (HPV-associated cancer) and L (Triple negative breast cancer):** antitumor activity will be assessed in terms of disease control rate at 6-month (6-month DCR rate) based on RECIST 1.1 criteria after in-stream centralized radiological review. 6-month DCR rate is defined as as the proportion of participants with confirmed complete response (CR), unconfirmed complete response (CRu), confirmed partial response (PR) or unconfirmed partial response (PRu) or stable disease (SD), as per adapted RECIST v1.1, observed within 24 weeks of treatment onset (while treated with the investigational product).

Secondary endpoints

- Best overall response is defined as the best response across all time points (RECIST v1.1). Following RECIST v1.1 recommendations:
  - The best overall response is determined once all the data for the patient is known.
  - The best overall response will be classified as confirmed complete response (CR), unconfirmed complete response (CRu), confirmed partial response (PR), unconfirmed partial response (PRu), stable disease or progressive disease, as per RECIST v1.1 criteria.
  - As per RECIST v1.1 criteria, to be considered as “confirmed”, complete and partial responses will have to be confirmed at least 4 weeks later to ensure responses identified are not the result of measurement errors.
- Objective response rate at 6 months (6-month ORR) is defined as the proportion of patients with objective response (confirmed or unconfirmed) at 6 months.
- Progression-free status is defined as complete response (confirmed or unconfirmed), partial response (confirmed or unconfirmed) or stable disease more than 24 weeks as defined as per RECIST v1.1 criteria (appendix 3). 6-month progression-free rate (6-month PFR) is defined as the proportion of patients with progression-free status at 6 months. 4-month progression-free rate (4-month PFR) is defined as the proportion of patients with progression-free status at 4 months.
- Growth modulation index (GMI): GMI is defined for each patient as the ratio of its PFS on Regorafenib + Avelumab treatment to its PFS on the previous line of therapy. This method accounts for inter-patient variability, the patient serving as his/her own control and implies by the natural history of the disease that the PFS tends to become shorter in successive lines of therapy. It is thought that an anti-cancer agent should be considered effective if the GMI is greater than 1.3
- Progression-free survival (PFS) is defined as the time from study treatment initiation to the first occurrence of disease progression or death (of any cause), whichever occurs first. 1-year PFS rate and median PFS will be reported.
- Overall Survival (OS) is defined as the time from study treatment initiation to death (of any cause). 1-year OS rate and median OS will be reported.
- Safety profile of the association Regorafenib + Avelumab: Toxicity will be graded using the common toxicity criteria from the NCI v5.0.
- Pharmacodynamic activity:
  - archived tumor tissue will be collected for assessment of the tumor microenvironment.
  - to perform integrative assessment of biomarkers of efficacy (genetic, metabolomics profiling in blood/tissue at baseline and different study time points) and its prognostic value on efficacy.
- For the cohort B: To evaluate the antitumor activity based on Choi criteria in terms of non-progression at 6 months.

**Statistical considerations**	**<u>NUMBER OF SUBJECTS NEEDED / DOSE ESCALATION PART</u>**<br>• The dose escalation part is designed to detect the MTD of Regorafenib given in association with Avelumab.<br>• The dose escalation design to identify the maximum tolerated dose will be the traditional 3+3 design. Adaptive designs, such a continual reassessment method (CRM) using likelihood inference do not appear relevant in this context given there are only three dose levels investigated and the toxicity profile of Regorafenib.<br>• A minimum of 3 patients and a maximum of 6 patients per dose level<br>• Since there are three dose levels investigated, a maximum of 12 eligible patients assessable for DLT is expected.<br><br>**<u>NUMBER OF SUBJECTS NEEDED / PHASE II TRIALS / COHORTS A, B, C, D, E, F, G</u>**<br>Independently for each of these phase 2 trials, a Bayesian approach will be used following an adaptive trial design, allowing for smaller and more informative trials, specifically tied to decision making within a drug development program. This process allows updating knowledge gradually rather than restricting revisions in a trial design with fixed sample sizes.<br><br>In each of the phase 2 trials:<br>• A maximum sample size of 50 patients will be included<br>• The analysis of the primary endpoint (ORR under treatment for cohorts A as well as C to G or 6-month PFR (for cohort B) will be carried out sequentially, with interim analyses planned after 16-week follow-up of the first 10 patients and then every 5 patients. Inclusions will not be suspended between interim analyses, except in case of an important accrual rate in a given trial (2 patients/center/months during at least 3 months).<br>• The probability of success (ORR under treatment (for cohorts A as well as C to G) or 6-month PFR (for cohort B) will be estimated from a beta-binomial model (Zohar et al., 2008). Initial parameters of the model will be pre-specified (the prior distribution represents the knowledge of the non-progression probability prior to observing the data). Successive results observed will then be used to update and refine the distribution, generating the so-called posterior distribution.<br>• In the absence of a strong idea about the response rates to be observed, a non-informative prior distribution (beta(1,1)) will be considered.<br>• At each update of the distribution (interim analysis), a stopping rule for inefficacy will recommend stopping the trial if there is a high predictive probability (≥80%) that the ORR (for cohorts A as well as C to G) or 6-month PFR (for cohort B) is lower or equal to the futility bound p0 (Strata A and E: p0=5%; Strata B/C/D/F: p0=20%; Stratum G: p0=10%).<br>• At each update of the distribution (interim analysis), a stopping rule for efficacy will recommend stopping the trial if there is a high predictive probability (≥80%) that the ORR (for cohorts A as well as C to G) or 6-month PFR (for cohort B) is higher or equal to the the maximal response probability threshold p1 (Strata A and E: p1=20%; Strata B/C/D/F: p1=40%; Stratum G: p1=25%).<br>• The trial will continue recruiting until the stopping rule applied at each interim analysis is met, or until the maximum sample size of 50 patients is reached.<br>• When a trial is not completed to allow efficacy analysis, descriptive data only are presented.<br><br>**<u>NUMBER OF SUBJECTS NEEDED / PHASE II TRIALS / COHORTS H, I and A'</u>**<br>• Single-arm phase 2 trial based on an optimal two-stage Simon's design<br>• Cohort H: 43 eligible and assessable patients (13 at stage 1) for a total of 47 patients to be included<br>• Cohort I: 29 eligible and assessable patients (13 at stage 1) for a total of 32 patients to be included<br>• Cohort A': 43 eligible and assessable patients (13 at stage 1) for a total of 47 patients to be included

CONFIDENTIAL

	**NUMBER OF SUBJECTS NEEDED / PHASE II TRIALS / COHORTS J to P**<br>• Single-arm phase 2 trial based on an exact single-stage A'Hern design<br>• Cohort J / L / N: 28 eligible and assessable patients for a total of 31 patients to be included<br>• Cohort K / P: 40 eligible and assessable patients for a total of 44 patients to be included<br>• Cohort M / O: 35 eligible and assessable patients for a total of 39 patients to be included<br><br>**STATISTICAL ANALYSES**<br>• Phase I trial (Dose escalation part)<br>o All analyses will be descriptive; no p-values will be calculated.<br>o Primary endpoint will be analyzed on the population assessable for safety of the phase I trial (escalation part).<br>o Toxicity observed at each dose level, graded according to the Common Terminology Criteria for Adverse Events v5.0 from the NCI, will be recorded in terms of event type, severity, dates of beginning and end, reversibility and evolution. Data will be gathered in tables summarizing toxicities and side effects for each dose level and cycle.<br>o DLT will be described in terms of number and incidence rates at each dose level. The number and percentage of patients who will have developed a DLT in each dose level will also be reported.<br>o Data analyses will be provided by dose groups and for all study patients, combined wherever appropriate.<br>o Categorical endpoints: best overall response, ORR under treatment and at 6 months, 6-month ORR and 6-month PFR, will be reported in terms of counts by dose level.<br>o Continuous endpoints, e.g. GMI, will be reported in terms of summary statistics that will include number of patients, median, minimum, and maximum, and additional percentiles if appropriate.<br>o Survival endpoints (PFS and OS) will be analyzed using the Kaplan-Meier method. The median survival rates will be reported with a 95% confidence interval. Median follow-up will be calculated using the reverse Kaplan-Meier method.<br>o Missing data will not be imputed.<br><br>• Phase II trials<br>o All analyses will be descriptive; no p-values will be calculated.<br>o Analyses will be conducted independently for each phase II trial.<br>o Analysis of the primary efficacy endpoint will be analysed based on the eligible population assessable for efficacy.<br>o Same statistical methods as for the phase I trial.

CONFIDENTIAL

Schedule of assessments and procedures

	SCREENING	*CYCLE 1*				*CYCLE 2*				*CYCLE N*	*DISC. VISIT*	*SAFETY FOLLOW-UP VISIT*
		**Wk1**	**Wk2**	**Wk3**	**Wk4**	**Wk1**	**Wk2**	**Wk3**	**Wk4**			
Regorafenib (per os)		From D1 to D21 continuously				From D1 to D21 continuously				D1 to D21		
Avelumab (i.v)Administration during day-hospitalization				D15		D1		D15		D1 + D15		
**Consultation**	**X**	**X**									**X**	**X**
**Regorafenib dispensation**		**D1**				**D1**				**D1**		
**Day-hospitalization**				**D15**		**D1**		**D15**		**D1 + D15**		
Written Informed consent	X											
Demographics data	X											
Medical history/baseline condition	X											
Concomitant treatments	X	Throughout the study										
Physical examination	X	D1		D15		D1		D15		D1	X	X
Assessment of signs and symptoms	X	Throughout the study										
Performance status (ECOG)	X	D1		D15		D1		D15		D1	X	X
Vital signs (heart rate, blood pressure, temperature)	X	D1		D15		D1		D15		D1 + D15	X	X
Height	X											
Weight	X	D1		D15		D1		D15		D1	X	X
Hematology[a]	X	D1	D8	D15	D22	D1	D8	D15	D22	D1 + D15	X	X
Biochemistry[b]	X	D1	D8[g]	D15	D22[g]	D1	D8[g]	D15	D22[g]	D1 + D15	X	X
Urinalysis[c]	X	D1		D15		D1		D15		D1	X	X
Thyroid test[h]	X	D1		D15		D1		D15		D1	X	X
ECG	X	Repeat if indicated										
Toxicity	Throughout the study											
Tumor measurement	X	Every 8 weeks										
Serum pregnancy test (*if indicated*)	X	X				D1				D1	D1	
PK[d] – *phase I*				D15		D1		D15				
Biomarkers[i]		D1				D1				D1[e]	D[f]	
Stool sample – *phase II (optional)*[j]		D1										
Tumor biopsy *(optional)*	*X*					*D1*						

a: differential WBC, haemoglobin, platelets (During treatment : D1, D8, D15 and D22 of cycle 1 and 2, and repeat before each Avelumab injection (ie. Day 1 and Day 15), at discontinuation visit and safety follow-up visit), aPTT, PT, INR (During treatment : D1, D15 of cycle 1 and 2, then D1 of each subsequent cycle, at discontinuation visit and safety follow-up visit)
b: albumin, alkaline phosphatase, total bilirubin, urea, calcium, chloride, creatinine, creatinine clearance, glucose, LDH, phosphorus, potassium, total protein, SGOT [AST], SGPT [ALT], GGT, sodium, CPK, lipase, amylase (Screening, C1D1 and D15, C2D1 and D15, then D1 of each subsequent cycle, at discontinuation visit and safety follow-up visit)
c:blood, glucose, protein, gravity (dipstick)
d: all blood samples will be collected on pre-dose (1 point)
e: D1C4, D1C6
f: at progression
g: only hepatic assessment (AST, ALT, alkalin phosphatase and GGT)
h: All cohorts :TSH at Screening, on Day 1 and Day 15 of Cycle 1and Cycle 2, then on Day 1 of each subsequent cycle, at discontinuation visit and safety follow-up visit. For cohort F only: Thyroglobulin and Antithyroglobulin antibody at Screening then repeat on Day 1 every 2 cycles, at discontinuation visit and safety follow-up visit.
i: Biomarkers : Blood samples at C1D1, C2D1, C4D1, C6D1 and at progression (pre-dose).
j: Stool sample (optional) for the microbiota only at C1D1 (pre-dose)

CONFIDENTIAL

LIST OF ABBREVIATIONS AND DEFINITION OF TERMS

**AE (s)**	Adverse Event (s)
**ALT**	Alanine Aminotransferase
**ANC**	Absolute Neutrophil Count
**ANSM**	Agence Nationale de sécurité du Médicament
**AP**	Alkaline Phosphatase
**aPTT**	Activate Partial Prothrombin Time
**AST**	Aspartate Aminotransferase
**AUC**	Area Under the Curve
**BP**	Blood Pressure
**BTC**	Biliary Tract Cancer
**CI**	Confidence Interval
**CNS**	Central Nervous System
**CPK**	Creatine PhosphoKinase
**CR**	Complete Response
**CRC**	Colo Rectal Cancer
**CRA**	Clinical Research Assistant
**CrCL**	Creatinine Clearance
**CRF**	Case Report Form
**CRM**	Continual Reassessment Method
**CT**	Computerized Tomography
**DLT**	Dose Limiting Toxicity
**DMP**	Data Management Plan
**DQF**	Data Query Form
**EC**	Ethic Committee
**ECG**	Electrocardiogram
**ECOG**	Eastern Cooperative Oncology Group
**EMEA**	European Medicines Evaluation Agency
**FCPRCC**	Fédération des Comités de Patients pour la Recherche Clinique en Cancérologie
**FFPE**	Formalin-Fixed Paraffin-Embedded
**FNCLCC**	Fédération Nationale des Centres de Lutte Contre le Cancer
**FUP**	Follow-Up
**GEP-NETs**	Neuroendocrine gastroenteropancreatic tumors
**GCP**	Good Clinical Practice
**GGT**	Gamma Glutyl-transferase
**GIST**	Gastro Intestinal Stromal Tumor
**GMI**	Growth Modulation Index
**HCC**	HepatoCellular Carcinoma
**HCG**	Human Chorionic Gonadotropin
**HIV**	Human Immunodeficiency Virus
**IB**	Investigator Brochure
**ICF**	Informed Consent Form
**IDMC**	Independent Data Monitoring Committee
**IEC**	Institutional Ethics Committee
**IHC**	Immunohistochemistry
**IMP**	Investigational Medical Product
**INR**	International Normalized Ratio
**IRB**	Institutional Review Board
**IUD**	IntraUterine Device
**IV**	Intraveinous
**LDH**	Lactate Dehydrogenase
**LFT**	Liver Function Test
**MAD**	Maximum Administered Dose
**MedDRA**	Medical Dictionary for Regulatory Activities
**MOA**	Mechanism Of Action
**MRI**	Magnetic Resonance Imaging
**MSI**	Micro Satelite Instability
**MTD**	Maximum Tolerated Dose
**NCI-CTCAE**	National Cancer Institute Common Terminology Criteria for Adverse Events
**NPR**	Non Progression Rate
**NSAIDs**	NonSteroidal Anti-Inflammatory Drugs
**NSCLC**	Non-Small Cell Lung Cancer
**NYHA**	New York Heart Association

CONFIDENTIAL

**ORR**	Objective Response Rate
**OS**	Overall Survival
**PD**	Progressive Disease
**PDGFR**	Platelet-Derived Growth Factor Receptor
**PFS**	Progression Free Survival
**PK**	Pharmacokinetic
**PR**	Partial Response
**PRE TT**	Pre-Treatment
**PTT**	Prothrombin Time
**RBC**	Red Blood Cell
**RECIST**	Response Evaluation Criteria in Solid Tumors
**RR-DTC**	Radioiodine-Refractory Differentiated Thyroid Cancer
**RP2D**	Recommanded Phase II Dose
**RRePS**	Réseau de Référence en Pathologie des Sarcomes des Tissus mous et des Viscères
**SAE**	Serious Adverse Event
**SD**	Stable Disease
**SFUP**	Safety Follow-up
**SmPC**	Summary Product Characteristic
**SPC**	Summary of Product Characteristics
**STS**	Soft Tissue Sarcoma
**SUSAR**	Suspected Unexpected Serious Adverse Reaction
**TAM**	Tumor Associated Macrophage
**TLS**	Tertiary Lymphoid Structure
**TME**	Tumor Micro-Environment
**ULN**	Upper Limit of Normality
**VEGFR**	Vascular Endothelial Growth Factor Receptors
**TT**	Treatment
**TSH**	Thyroid Stimulating Hormon

CONFIDENTIAL

1. RATIONALE OF THE TRIAL

1.1. PATHOLOGIES OF INTEREST

1.1.1.Management of colorectal cancer in advanced setting (cohorts A – A')

Colorectal cancer is the second most frequent cancer worldwide, account for approximatively 13% of all cancer cases in men and women. Twenty five percent of patients present with metastases at initial diagnosis and 50% of patients with CRC will develop metastasis which will contribute to the high mortality rate of the disease. Five year survival rate is estimated around 60% [1]. Unless the cases of potential resectable metastases, the main treatment of metastatic CRC (mCRC) is palliative systemic combinaison of molecules. For the first line, it is recommended a doublet of cytotoxics associated with a targeted agent, most often bevacizumab [1]. For the lines after: doublet of cytotoxics associated whenever possible with anti-EGFR agents [2, 3]. Oxaliplatin combinations can be de-escalated to 5-FU/LV as maintenance treatment after a few months [4]. The use of anti–EGFR antibodies is conferred to RAS wild-type tumors [5]. The ultimate line to be considered is an oral targeted therapy: Regorafenib which has demonstrated a gain of 1.5 months of overall survival compared to placebo [6]. Since then, no advances have been done in mCRC and median survival do not exceed 30 months (patients in clinical trials). Therefore, progresses are needed in this disease.
It has been shown that high dose of antiangiogenic agents often used in animal models, can lead to shorter time windows of normalization due to rapid and excessive pruning of tumor vessels and resulting hypoxia. This may further restrict the access of effector T cells into tumor parenchyma, enhance the immunosuppressive tumor microenvironment, and thus compromise the efficacy of an anticancer immunotherapy. Several preclinical experiments have supported this concept of dose-dependent normalization of vessels with anti-VEGF therapy and found that lower doses of anti-VEGFR2 antibody therapy was superior to high-dose in facilitating T-cell tumor infiltration and at polarizing perivascular TAMs from an M2-like to an M1-like phenotype [88].
Clinical data seems to support these preclinical findings. Indeed, a recent study investigating the combination of nivolumab and regorafenib in chemotherapy-refractory metastatic colorectal cancer patients (microsatellite stable) showed a remarkable objective response rate 36% and a median PFS of 6.3 months despite the fact that the majority of the patients were treated with a low dose of regorafenib (80 mg) [90].

1.1.2.Management of gastrointestinal stromal tumors in advanced setting (cohort B)

Gastrointestinal stromal tumors (GIST) are the most frequent mesenchymal tumors of the gastrointestinal tract. In most cases of GIST, somatic mutations can be found in the gene encoding the KIT protein, typically in exons 11 and 9 (more rarely in exon 13 or exon 17). These mutations confer a gain of function to the receptor, which becomes constitutively activated regardless of the presence of its ligand, stem cell factor. In some cases of GIST, no mutation is found in KIT. The search for additional molecular abnormalities led to the discovery that in many KIT-negative GIST there is a somatic mutation in the gene encoding the platelet-derived growthfactor receptor (PDGFR). PDGFR is closely related to KIT, and also belongs to the type III family of receptor tyrosine kinases. Mutated KIT or PDGFR confer a growth advantage to tumor cells, and have recently become targets for therapeutic intervention. Imatinib is an oral tyrosine-kinase inhibitor that has revolutionized the treatment of GIST, since this drug is able to inhibit the tyrosine-kinase activities of KIT and PDGFR. Imatinib was approved in February 2002 for the treatment of patients with metastatic and/or unresectable GIST [7]. However, resistance to imatinib will develop in almost all patients and represents a clinical challenge. Others tyrosin kinase inhibitors have been approved in this indication : Sunitinib and Regorafenib [8, 9]. Nevertherless, patients will undoubtely undergo ultimate progression of their disease.

1.1.3.Management of advanced oesophageal and gastric carcinoma (cohort C)

The incidence of oesophageal and gastric carcinoma is 3.9 and 4.9/100000 respectively. Both have a very poor prognosis with a 5-year survival rates of 10 and 15% respectively [10, 11]. Squamous cell carcinoma of the oesophagus is link with smoking and alcohol consumption while adenocarcinoma is link with gastro-oesophageal reflux disease. In a metastatic setting, palliative chemotherapy is the indicated treatment when the performans status of the patient allows it, and the number of therapeutic solutions is very limited. The classical schedule for both is 5-FU/cisplatin with a possible alternative combining oxaliplatin and fluoropyrimidine, or tri-therapy schedules whenever possible [12, 13]. Taxanes can be considered as second line therapy [10, 14]. The use of a targeted therapy in adenocarcinoma of the oesophagus or gastric carcinoma is limited to a small proportion of patients with HER2-positive tumors (10 to 15%). Thus, trastuzumab can be associated with capecitabin or 5FU plus cisplatin as first line treatment [15]. Additionnally, an anti-VEGFR2 antibody has been recently approved in gastric carcinoma associated with paclitaxel as second line of treatment. Ramucirumab has improved the overall survival by 2.3 months in the Rainbow study [16].

CONFIDENTIAL

Regarding the dramatic prognosis of these 2 diseases in the metastatic setting and the poor efficacy of the validated treatments, the situation represents an unmet medical need.

1.1.4.Management of biliary tract cancer and hepatocellular carcinoma (cohort D)

The incidence of biliary tract cancer (BTC) is 3.2 and 5.4 /100000 for male and female respectively. The incidence of hepatocellular carcinoma (HCC) is variable, depending on the worldwide distribution of viral hepatitis B and C [17, 18]. In advanced or metastatic setting, both of the diseases confer worse prognosis, with a 5-year survival of 12-15 months for BTC, and <8 months for HCC, dominated by the level of subsequent cirrhosis in patients with HCC [19]. Only one line is validated in the metastatic setting for BTC, based on the association of gemcitabine and cisplatin or oxaliplatin [20] with no validated second line after progression. Classic cytotoxics have not demonstrated overall survival improval in HCC. Sorafenib, an oral drug which blocks PDGF, VEGF, c-Kit and raf signaling, is the standard treatment in metastatic HCC patients who have a preserved liver function [21]. The lack of validated treatments in these diseases in the metastatic setting arises the urgent need of new therapeutic solutions.

1.1.5.Management of Soft Tissue Sarcoma (STS) (cohorts E – I)

Soft tissue sarcomas (STS) constitute a heterogeneous group of mesenchymal tumors that account for 1% of adult cancers. These tumors are mostly classified according to their eventual line of differentiation determined along morphology and immuno-histochemical (IHC) patterns. They can be further classified into two main genetic types:

- sarcomas with ‘simple genomics’ associated with a simple genomic alteration, such as a specific translocation as in synovialosarcomas;
- and sarcomas with ‘complex genomics’ (SCG), characterized by very complex karyotypes and genomic profiles in array-comparative genomic hybridization (CGH). This group is mainly composed of tumors with spindle or pleiomorphic cells, corresponding to leiomyosarcomas (LMS) and undifferentiated pleiomorphic sarcomas (UPS).

Despite adequate loco-regional treatment, up to 40% of patients with sarcoma, soft tissue or bone, will develop metastatic disease. When metastases are detected, the standard of care is based on palliative chemotherapy with a median survival in this setting of only 18 months.
A slight improvement has been obtained over years thank to registration of a couple of drugs such as Trabectedin and Pazopanib, the first antiangiogenic registered for sarcoma patients [50]. Pazopanib is routinely prescribed worldwide after failure of first line chemotherapy. However after failure of these agents, the survival is very poor. Sarcomas are therefore tumors with an urgent unmet medical need.

PDL1 expression is part of an immune inhibitory pathway with high therapeutic potential. Recent studies have shown that PDL1 is variably expressed in STS [51-53] leading to speculate that drugs targeting this immune checkpoint would be of interest in STS, and particularly in sarcomas with complex genomics such as LMS and UPS.
Results of the PEMBROSARC trial conducted in France, assessing the combination of Pembrolizumab, a selective PD-1 inhibitor from Merck, and metronomic cyclophosphamide, have been published recently published in the JAMA Oncology. In this study, only one patient with a progressive solitary fibrous tumor experienced a partial response [54].
Importantly, correlative studies performed in the PEMBROSARC trial shed lights on the immune landscape of STS and GIST and allowed new therapeutic hypotheses:
Indeed, assessment of tumor samples from patients included in the study demonstrated that PDL1 expression on tumor cells was ≥1% in 6 (12%) of the 49 patients with available marker whereas PD-L1 expression on immune cells was ≥1% in 19 (40%) of the 48 patients assessable for this marker, and ranged from 23% in LMS to 64% in UPS. The patient with the best partial response was the only one with a PDL1 expression > 10% on immune cells. These results both underline the importance of better selecting the right population for antiPD1 targeting in STS, and lead to speculate that other inhibitory pathways may be involved in resistance to anti-tumor immunity in these tumors.
Noteworthy, correlative assessment of tumor samples from the PEMBROSARC study showed strong macrophages presence, with a large preponderance of M2 phenotype. M2 macrophages are known to be pro-tumoral immunosuppressive cells and their role as a resistance mechanism to immune surveillance is well documented [55-56]. STS are known to be infiltrated with macrophages, especially STS with complex genomics such as LMS, and this has been demonstrated to be of poor prognosis [57-58]. Importantly, M2 Macrophages are known to promote angiogenesis [59].
Regorafenib inhibits multiple receptor tyrosine kinases (RTKs) implicated in tumor growth, metastasis, and angiogenesis (Investigator’s Brochure). Regorafenib has shown very promising preliminary activity in a phase II trial in soft-tissue sarcomas [23] (REGOSARC trial, ongoing).
In preclinical studies, while immune checkpoint inhibitors were insufficient in controlling tumour growth, combining them with M2 macrophage or angiogenesis targeting resulted in superior tumour control [60, 61].

CONFIDENTIAL

Given the ancillary results of the PEMBROSARC trial, the importance of M2 macrophage infiltration and the potential of angiogenesis targeting in sarcoma, further strategies assessing combination of a PD-1/PDL1 inhibitor and an antiangiogenic agent are warranted.
Moreover, a recent study investigating the transcriptomic 608 tumours across STS subtypes, established an immune-based classification based on the composition of the tumour microenvironment (TME), identifying five distinct phenotypes: immune-low (A, B), immune-high (D, E), and highly vascularised (C) groups. In situ analysis of an independent validation cohort showed that class E was also characterized by the presence of B cell rich tertiary lymphoid structures (TLS). The immune-high E group demonstrated improved survival and a high response rate to PD-1 blockade by pembrolizumab. Altogether, this work provides novel evidence regarding the presence of immune subtypes in STS patients, and unravels the potential of TLS to be used as a biomarker for immunotherapy-driven clinical trials in soft-tissue sarcomas [63,64].

1.1.6.Management of Radioiodine-Refractory Differentiated Thyroid Cancer (RR-DTC) (cohort F)

Distant metastases are observed at diagnosis in less than 5% of DTC patients, and recurrent disease occurs in 10-15%. Radioiodine treatment may be efficient in case of distant metastases, especially in young patients with well differentiated tumors and small lung metastases. Even if the vast majority of metastatic patients benefit from 131I, the treatment is not efficient in all cases. Indeed, the disease might progress in about 10% of patients as a consequence of tumor dedifferentiation and consecutive loss of radioiodine uptake, despite well-conducted initial treatment.

In these cases of progressive radioiodine-refractory differentiated thyroid cancer (RR-DTC), targeted therapies that inhibit the VEGF pathway have demonstrated their effectiveness. Lenvatinib and sorafenib were approved for the treatment of patients with progressive RR-DTC both by the US Food and Drug Administration and by the European Medicines Agency [46].

However, secondary resistance is observed in the majority of cases with no real second-line treatment available. Various studies found a very strong implication of programmed death ligand 1(PD-L1) in the aggressiveness of thyroid cancers [47]. PD-L1 expression is also a prognostic factor [48]. However, a promising activity of Pembrolizumab has been detected in RR-DTC patients (Keynote 028) (Abstract ASCO2015).

Although it has been little evaluated, Regorafenib (administered orally) is a small molecule inhibitor against multiple tyrosine kinases, including VEGFR1/2/3, PDGFR, Kit, RET and Raf-1 (similar to the treatment use in RR-DTC), could be an effective treatment in RR-DTC.
It's association with Avelumab (a human monoclonal antibody that inhibits PD-L1) could have a synergistic immuno-stimulating and antiangiogenic activity in RR-DTC.
This combination, which could be a real therapeutic alternative in the second line of treatment, needs a therapeutic evaluation.

1.1.7.Management of Neuroendocrine gastroenteropancreatic tumors (GEP-NETs) (cohort G)

GEP-NETs are a rare heterogeneous population of tumors classified based on their anatomic site of origin. Their incidence has significantly increased over the last years and is estimated to be 5.25/100,000/year. The WHO classification defines three main categories from well-differentiated NETS (low grade) to poorly differentiated (high-grade). GEP-NETs share a common phenotype with immunoreactivity for the « pan-neuroendocrine » markers including chromogranin A and synaptophysin. Neuron-specific enolase (NSE) and CD56 are less specific. The 5-year survival rates for metastatic disease varies from 35% for well-differentiated G1/G2 NETs to 4% for poorly differentiated disease. For this reason, there is a need of finding new therapeutic solutions in this disease. Of note, PD-L1 expression has been reported in 21.9% of GEP-NETs [49].

1.1.8.Management of non-small cell lung cancer (NSCLC) (cohort H)

Treatments for patients with advanced NSCLC are palliative, and thus intended to prolong survival and preserve quality of life for as long as possible, while minimizing the side effects due to treatment. Immune checkpoint inhibitors targeting either programmed cell death protein 1 (PD-1) or programmed cell death ligand 1 (PD-L1) have become routinely part of the clinical approach for management of NSCLC. Pembrolizumab has received European Medicines Agency (EMA) and US Food and Drug Administration (FDA) approval for the frontline treatment of metastatic nonsquamous NSCLC in combination with platinum-based chhemotherapy irrespective of programmed cell death ligand 1 (PD-L1) expression.
Targeting angiogenesis remains an important therapeutic strategy in the management of NSCLC. Several studies have found that adding angiogenic inhibitors to standard chemotherapy iporved outcome of NSCLC patients. Therefore several antiangiogenic therapies have been approved in the

CONFIDENTIAL

second line by the U.S. Food and Drug Administration (bevacizumab, ramucirumab) and the European Medicines Agency (nintedanib) in this setting.
Some preclinical evidence also suggests that immunotherapy combined with antiangiogenic agents can potentially improve the efficacy of patients with advanced NSCLC [65-66]. For example, in an in vivo lung adenocarcinoma model, immunotherapy combined with bevacizumab synergistically inhibits tumor growth [66]. In the preclinical model of lung cancer, PD-L1 mAb combined with VEGFR2 small molecule inhibitor can significantly downregulate the expression of PD-1 and PD-L1, increase TILs, and inhibit tumor growth by reducing Tregs and MDSCs [67].

1.1.9. Management of urothelial cancer (cohort J)

Metastatic urothelial cancer is traditionally treated by a first platinum-based polychemotherapy (MVAC or Gemcitabine-Cisplatin), with an overall response rate of approximately 50% and a median progression-free survival of 7 months. Maintenance avelumab is associated with an overall survival advantage compared with best supportive care in patients who did not have disease progression after 4 to 6 cycles of gemcitabine plus cisplatin or carboplatin, and is now recommended [Powles T, Park SH, Voog E, et al. Avelumab maintenance therapy for advanced or metastatic urothelial carcinoma. N Engl J Med. 2020;383(13):1218-1230]. Second line usually consists of taxane-based regimen, with an overall 20% response rate [78]. However, in the metastatic setting, the prognosis remains poor.
VEGF and VEGFR are expressed in tumoral urothelial tissus and are correlated to poor prognosis [Wu W, Shu X, Hovsepyan H, et al. VEGF receptor expression and signaling in human bladder tumors. Oncogene. 2003;22:3361–70 et Streeter EH, Harris AL. Angiogenesis in bladder cancer–prognostic marker and target for future therapy. Surg Oncol 2002;11:85–100]. Anti angiogenic agents results in the treatment of urothelial cancer have been disappointing [César Serrano 1 , Rafael Morales, Cristina Suárez, Isaac Núñez, Claudia Valverde, Jordi Rodón, Jordi Humbert, Olga Padrós, Joan CarlesCancer Treat Rev. 2012 Jun;38(4):311-7]. However, combined with immune checkpoint inhibitors, they possibly work synergistically [Dirkx AE, oude Egbrink MG, Castermans K, et al. Anti-angiogenesis therapy can overcome endothelial cell anergy and promote leukocyte-endothelium interactions and infiltration in tumors. FASEB J. 2006;20:621-630; Yasuda S, Sho M, Yamato I, et al. Simultaneous blockade of programmed death 1 and vascular endothelial growth factor receptor 2 (VEGFR 2) induces synergistic anti-tumour effect in vivo. Clin Exp Immunol. 2013;172:500-506].
As consequence, the avelumab and regorafenib combination appears attractive in this population in order to reverse the resistance to anti PD1/PD-L1.

1.1.10. Management of HPV-associated cancer (cohort K) with molecular confirmation of p16 positive status

Human papilloma virus (HPV) infections are involved in most cervix, anal, vulva, vagina, penis and some head and neck squamous cell carcinoma cases [Osborne, Maykel et al.2014, Dotto and Rustgi 2016, Brianti, De Flammineis et al. 2017, Brotherton, Budd et al. 2020).]. The prognosis widely depends on the stage of these cancers. However, in the metastatic setting, the prognosis remains poor.
Data are controversial regarding the possible response of HPV-mediated cancers to checkpoint inhibitors and combination of treatments may increase the anti tumor effect of immunotherapy in these tumors [Fakhr Immunotherapy 2021].
Thus, the regorafenib+ avelumab combination appears as an interesting alternative therapeutic in this setting to be evaluate.

1.1.11. Management of triple negative breast cancer (cohort L)

Brest cancer (BC) is the most commun cancer in women worldwide. Treatment of advanced/metastatic BC is dominated by hormonotherapy for HR+ tumors and conventional chemotherapy regimen for eventually all patients, including anthracyclines, taxanes and several other agents such as capecitabine, gemcitabine or more recently eribulin [Cortes J, O'Shaughnessy J, Loesch D et al. Eribulin monotherapy versus treatment of physician's choice in patients with metastatic breast cancer (EMBRACE): a phase 3 open-label randomised study. Lancet 2011; 377: 914-923.]. Triple-negative breast cancer (TNBC) is a particular sub group of BC defined by distinct molecular sub types with variable outcomes [Burstein M.D., Tsimelzon A., Poage G.M., Covington K.R., Contreras A., Fuqua S.A., Savage M.I., Osborne C.K., Hilsenbeck S.G., Chang J.C. Comprehensive genomic analysis identifies novel subtypes and targets of triple-negative breast cancer. *Clin. Cancer Res.* 2015;21:1688–169].
Immune checkpoint inhibitors have demonstrated their interest in the metastatic setting of TNBC with first the accelerated approval for the PD-L1 antagonist atezolizumab (Tecentriq) in combination with chemotherapy for the 1st line treatment of patients with locally advanced or metastatic TNBC that cannot be treated surgically and whose tumors are positive for PD-L1. FDA’s approval was based on results from the phase 3 IMpassion130 clinical trial, which compared atezolizumab plus nab-paclitaxel with placebo plus nab-paclitaxel as the initial, or first-line, treatment for patients with triple-negative breast cancer [Schmid P, Adams S, Rugo HS, Schneeweiss A, Barrios CH, Iwata H, Diéras V, Hegg R, Im SA, Shaw Wright G, Henschel V, Molinero L, Chui SY, Funke R, Husain A, Winer EP, Loi S,

CONFIDENTIAL

Emens LA; IMpassion130 Trial Investigators. Atezolizumab and Nab-Paclitaxel in Advanced Triple-Negative Breast Cancer. N Engl J Med. 2018 Nov 29;379(22):2108-2121.]. Then the results of the checkmate-355 showed the benefit of pembrolizumab (KEYTRUDA) adjonction to chemotherapy versus chemotherapy alone in the same population and led to FDA's approval tpp [Lancet. 2020 Dec 5;396(10265):1817-1828.Pembrolizumab plus chemotherapy versus placebo plus chemotherapy for previously untreated locally recurrent inoperable or metastatic triple-negative breast cancer (KEYNOTE-355): a randomised, placebo-controlled, double-blind, phase 3 clinical trial Javier Cortes [1], David W Cescon [2], et al.].
However, initial responders patients will eventually progressed under immunotherapy maintenance alone and there is a need to overcome this resistance. In that purpose, anti angiogenic + checkpoint inhibitor association could have this synergistic immuno-stimulative effect in this population.

1.1.12. Management of TMB-high solid tumors (cohort M) with TMB-high status already known

TMB, quantified as the number of somatic mutations per length of tumor DNA analyzed, is an interesting biomarker of response to immune checkpoint blockade [Yarchoan M, Hopkins A, Jaffee EM. Tumor mutational burden and response rate to PD-1 inhibition [correspondence]. N Engl J Med. 2017 Dec 21;377(25):2500-1]. The main issue regarding this biomarker is that there is no universally valid TMB cut-off available for all cancer types to determine which tumor will respond to immune checpoint inhibitors [Zheng M. Tumor mutation burden for predicting immune checkpoint blockade response: the more, the better. J Immunother Cancer. 2022 Jan;10(1):e00308]. A large review of patients who had undergone NGS and received checkpoint inhibitor therapy revealed that higher TMB was independently associated with better outcomes with notable response rate (RR) [Goodman AM, Kato S, Bazhenova L, Patel SP, Frampton GM, Miller V, et al. Tumor mutational burden as an independent predictor of response to immunotherapy in diverse cancers. Mol cancer therapeutics. 2017;16(Suppl 11):2598–608]. When evaluating the TMB stautus in a cohort of solid tumors patients treated with pembrolizumab in a prospective phase II trails, high-TMB identified a sub group of patients more likely to respond to thet tratment [Marabelle 2020 Lancet oncol.]
We believe that the association of regorafenib + Avelumab may enhance the response to treatment in patients with TMB-High advanced solid tumors, defined as $\geq$ 16 mutations per Mb

1.1.13. Management of MSI-high solid tumors (cohort N) with MSI-high status already known

High level of microsatellite instability (MSI-H) is the genetic feature of a well-defined subgroup of cancers characterized by a deficient mismatch repair (dMMR) system. This status leads to an inability to correct damage to DNA that primarily derives from single base pair insertions or deletions that may occur during DNA replication by DNA polymerases. MSI-H tumors patients have demonstrated interesting outcomes when treated with immune checkpoint inhibitors.
Indeed, not only on MSI-high colorectal cancer patients, whatever the studied primary tumors, MSI-high status is associated with better outcomes when the patients are treated with pembrolizumab [André T. et al. N Engl J Med. 2020 Dec 3;383(23):2207-2218 ; Chao J. et al. JAMA Oncol. 2021 Jun 1;7(6):895-902 ;O'Mailly et al. J Clin Oncol. 2022 Mar 1;40(7):752-761]. Similar data have been previously reported with nivolumab + ipilimumab association [Overman MJ. Et al. J Clin Oncol. 2018 Mar 10;36(8):773-779].

However, despite an improved response rate with anti PD-1 treatment in patients with MSI-high solid tumors, some patients do not response to the immunotherapy and we believe that combination with anti angiogenic molecule may icnrease such results.

1.1.14. Management of Non-clearcell renal carcinoma (cohort O)

Non-clear cell renal cell carcinomas are a genetically and histologically diverse subgroup of cancers that arise from the kidney. They include several histologies such as : pappillary renal cell carcinoma, chromophobe renal cell carcinoma, translocation carcinoma and other rare subtypes. They represent 25% of renal cell cancers. Due to this rareness, these cases are mostly excluded from phase 3 studies. The response observed with pembrolizumab treatment treatment depends mostly on the histology subtypes [MvDermott et al. J Clin Oncol. 2021 Mar 20;39(9):1029-1039]. Combination of nivolumab + Lenvatinib has demonstrated very interesting data with an overall response rate of 47% [Lee et al.].

According to these results, the avelumab+ regorafenib combination seems very promising in non-clear cell renal cell carcinomas.

1.1.15. Management of Malignant pleural mesothelioma (cohort P).

Malignant pleural mesothelioma (MPM) is a rare and aggressive disease arising from the mesothelial surface of the pleura. It is almost always caused by asbestos exposure. Histologic subtypes of MPM include epithelioid, non epithelioid including sarcomatoid, biphasic or others for 60 and 40% respectively. The prognosis is very poor, with median of overall survival around 9 to 12 months [Vogelzang et al. J Clin Oncol. 2003; 21(14):2636-44]. Since recently, the main therapeutic option in unresectable cases was a combination of cisplatin and pemetrexed +/- bevacizumab, allowing a response rate of 40% and a median overall survival of 18.8 months in the MAPS trial [Zalcman G etal. Lancet 2016; 387 (10026):1405-14].
However, immunotherapy has recently changed this landscape. The checkmate-743 study has demonstrated the benefit of the nivolumab + ipilimumab association over standard-of-care chemotherapy to improve meaningfully the median overall survival in previously untreated MPM patients and is now validated as preferred fiorst line of treatment [Baas P. et al. Lancet 2021; 397: 375–86].
However, the majority of the patients will ultimately progress under this treatment, and epithelioid sub group will not benefit of this combination as the sarcomatoid subgroup. Thus, it appears interesting to propose to previously treated MPM patients the combination of avelumab and regorafenib.

1.2. REGORAFENIB

1.2.1.Chemical structure

The multitargeted receptor tyrosine kinase inhibitor Regorafenib [Stivarga®, BAY 73-4506] is an orally bioavailable small molecule with potential antiangiogenic and antineoplastic activities. Its chemical name is [4-[4-[[4-chloro-3-(trifluoromethyl)phenyl]carbamoylamino]-3-fluorophenoxy]-N-methylpyridine-2-carboxamide].
The Regorafenib 40 mg tablet is coated, not divisible, gray-orange-red, oval (length 16 mm, width 7 mm, thickness 4.9-5.6 mm) and 472 mg each in total weight.

1.2.2.Preclinical data and mechanism of action

Regorafenib is a small molecule inhibitor of multiple membrane-bound and intracellular kinases involved in normal cellular functions and in pathologic processes such as oncogenesis, tumor angiogenesis, and maintenance of the tumor microenvironment. Regorafenib binds to and inhibits vascular endothelial growth factor receptors (VEGFRs) 2 and 3, and Ret, Kit, PDGFR and Raf kinases, which may result in the inhibition of tumor angiogenesis and tumor cell proliferation. VEGFRs are receptor tyrosine kinases that play important roles in tumor angiogenesis; the receptor tyrosine kinases RET, KIT, and PDGFR, and the serine/threonine-specific Raf kinase are involved in tumor cell signaling. In in vitro biochemical or cellular assays, Regorafenib or its major human active metabolites M-2 and M-5 inhibited the activity of RET, VEGFR1, VEGFR2, VEGFR3, KIT, PDGFR-alpha, PDGFR-beta, FGFR1, FGFR2, TIE2, DDR2, TrkA, Eph2A, RAF-1, BRAF, BRAFV600E, SAPK2, PTK5, and Abl at concentrations of Regorafenib that have been achieved clinically. In in vivo models, Regorafenib demonstrated anti-angiogenic activity in a rat tumor model, and inhibition of tumor growth as well as anti-metastatic activity in several mouse xenograft models including some for human colorectal carcinoma [22].

1.2.3.Clinical data

Regorafenib (BAY 73-4506) is an orally active and highly potent inhibitor against multiple tyrosine kinases, including VEGFR1/2/3, PDGFR, Kit, RET and Raf-1. It has demonstrated a significant benefit in overall survival when given at 160mg once daily, for the first 3 weeks of each 4 week cycle in colorectal cancer [6] and gastrointestinal stromal tumors patients with metastatic disease and progression during their last standard therapy [9]. Regorafenib has also demonstrated efficacy in advanced soft-tissue sarcomas and in hepatocellular carcinoma [23,43] and is currently investigated in various other solid tumors such as biliary cancer, pancreatic cancer, neuro endocrine tumors and gastrooesophageal carcinoma (clinicaltrials.gov).
The most common drug-related, treatment-emergent adverse events (occurring in at least 10%) include hand-foot skin reaction (56%) hypertension (48%) diarrhea (41%) fatigue (38.6%) oral mucositis (38%) alopecia (23.5%) hoarseness (22%) anorexia (20.5%) maculopapular rash (18%) nausea (15.9%), constipation (15.2%), myalgia (13.6%), and voice alteration (11.4%) [9].

1.3. AVELUMAB

1.3.1.Chemical structure

Avelumab [MSB-0010718C, PF-06834635] is a human monoclonal antibody (mAb) of the immunoglobulin G1-lambda1, subclass that inihibits binding of programmed cell death ligand 1 (CD274

CONFIDENTIAL

(programmed death ligand 1, PDL1, pd-l1, B7 homolog 1, B7H1)) to programmed cell death 1 (PD-1, CD279) and CD80 (B7-1). Its molecular weight is 143832 Dalton.

1.3.2.Preclinical and mechanism of action

Avelumab is an investigational fully human IgG1 monoclonal antibody that selectively binds with high affinity (0.7nM) and specificity (it does not bind any other B7 family) to human PD-L1 (programmed death-ligand 1) and blocks its interaction with PD-1 and CD80. In vitro studies [24] show that Avelumab antagonizes the inhibitory effect of PD-L1 on primary human T cells and enhances antigen-specific T-celle response. In addition, it is demonstrated that Avelumab elicits antibody-dependent cellular cytotoxicity as a secondary mechanism for its antitumor activity in cell-based functional assays [25]. In vivo studies show that Avelumab inhibits tumor growth in a mouse model via a T-lymphocyte (T-cell) dependent mechanism [26].

1.3.3.Clinical data

Avelumab is an investigational molecule. JAVELIN is a multipronged phase Ib clinical trial which to date has enrolled more than 1,000 participants with various types of advanced cancer. Avelumab has shown antitumor activity in patients with lung, gastric, bladder, ovarian, and others cancers [44,45]. Phase III randomized trials are underway evaluating Avelumab in patients with advanced non-small cell lung cancer and gastric cancer. Avelumab has a very favourable toxicity profile that allows even more powerful combinations. Several studies are presently ongoing (clinicaltrials.gov). Recently, Avelumab has demonstrated efficacy in Merkel cell carcinoma with favourable toxicity profile [27]. No grade 4 or death related to the treatment occurred. Serious related adverse events were uncommon (6% of patients) in this study [27]. The most common drug-related, treatment-emergent adverse events (occurring in at least 5%) include: fatigue (24%), infusion-related reaction (17%), diarrhea (9%), nausea (9%), asthenia (8%), rash (7%), decreased appetite (6%), maculopapular rash (6%)[27].

1.4. STUDY RATIONALE

Immunotherapy has introduced a paradigm shift in the treatment of solid tumors. Our pathologies of interest in this protocol have an interesting immunologic background.
Consistent with potentially high immunogenicity, MSI-H colorectal cancer is associated with high numbers of tumor-infiltrating lymphocytes and a Crohn-like host response [28]. PD-L1 immune checkpoint regulation has also been directly implicated in colorectal cancer pathogenesis. Thus, PD-L1 was expressed on tumor cells in 53% of patients with colon adenocarcinoma [29]. A case report has been published describing a patient with PD-L1-positive MSI-H colorectal cancer who exhibited a long-term response with the anti-PD-1 antibody BMS-936558 [30]. An impressive activity of pembrolizumab has been aslo recently reported in MSI colorectal tumors [31]. Finally, the anti-PD-L1 antibody MPDL3280A demonstrated preliminary activity against colorectal cancer in Study PCD4989g as a single agent and in Study GP28328 in combination with bevacizumab (https://clinicaltrials.gov/ct2/show/NCT01633970).
PDL1 expression represents an independent prognostic factor in GIST patients [32]. Strikingly, KIT inhibition was associated with potentiation of antitumor T cell responses in gastrointestinal stromal tumor through the inhibition of Ido [33]. Moreover, PD-1/PD-L1 blockade has been shown to synergize with KIT inhibition in a pre-clinical model of gastrointestinal stromal tumor [34].
PDL1 expression represents also a prognostic factor in esophageal and gastric cancer [35, 36]. A promising activity of Pembrolizumab has been detected in patients with gastric cancer in the phase Ib Keynote 012 [37] and in the phase Ib Keynote 028 in esophageal cancer patient [38].
Same kinds of data exist in HCC. Nivolumab, an anti PD1 antibody, has demonstrated interesting anti tumor activity [39] in this disease. Checkpoint inhibitors are under investigation in BTC (clinicaltrials.gov).
Targeted therapies act by blocking essential biochemical pathways or mutant proteins that are required for tumor cell growth and survival. Recent preclinical data suggest synergy between targeted therapies and immunotherapy. For instance, immunity and angiogenesis are tightly connected [40]. The abnormal tumor vasculature creates a hypoxic microenvironment that polarizes inflammatory cells toward immune suppression. Moreover, tumors systemically alter immunes cells proliferation, differenciation, and function via secretion of growth factors and cytokines such as VEGF, that plays a critical role in immunosuppression. Hence, antiangiogenic treatment may be an effective modality to potentiate immunotherapy [41]. Recent preclinical data have indeed shown that combination of antiangiogenic therapy with immunotherapy can exert synergistic antitumor effect [42].
Altogether, these findings provide a strong rational for assessment of Regorafenib combined with Avelumab in patients with digestive cancers. We hypothesized that the association of Regorafenib and Avelumab could have a synergistic immuno-stimulating and antiangiogenic activity in patients with advanced digestive cancers with PDL1 expression on IHC and more specifically in colorectal Cancer and GIST.

CONFIDENTIAL

Overcoming immunosuppression induced by VEGF as well as by its blockade may be potentially achieved by using different strategies. One approach could be careful titration of VEGF inhibition to inhibit VEGF pathway and angiogenesis while avoiding excessive pruning and hypoxia. For example, Huang et al. showed that dose-titrated anti-VEGFR2 antibody therapy can alleviate hypoxia (via vascular normalization) and potentiate the effects of vaccination in a mouse model of breast cancer [88]. By using a low-dose anti-VEGFR2 antibody, effector T-cell infiltration increased compared with high-dose anti-VEGFR2 treatment. In addition, tumor-infiltrating macrophages showed a more immune stimulatory (M1) phenotype. Similar results have reported using TNF-α blockade in the mouse models. While TNF-α blockade itself can alleviate immune suppressive phenotype on immune cells, vascular destruction by TNF-α blockade resulted in increased tumor hypoxia and paradoxically caused immune suppression [89].
A recent study investigating low-dose regorafenib (80 mg/day instead of standard 160 mg/day) in combination with nivolumab has demonstrated a high-level of activity in a population of japanese patients with chemo-refractory colorectal and gastric cancer patients [90]. We hypothesize that such anti-tumor efficacy is achievable in Caucasian patients.

1.5. BENEFIT/RISK AND ETHICAL ASSESSMENT

Some clinical trials (phase 1 trials essentially) are ongoing to explore the role of targeted therapies in combination with immunotherapies but no final results with Regorafenib are available. Combination with sunitinib or pazopanib in metastatic renal cancer patients showed similar proportion of treatment discontinuation due to adverse events (preliminary results). Data of Avelumab alone AND Regorafenib alone have proved that both can be safely used. Thus, it can be expected that Regorafenib, even administered at a daily dose of 120mg (which is lower than the recommended one for patients treated at the RP2D) or a low-dose of 80 mg/day in combination with Avelumab would increase the anti-tumor efficacy while maintaining an acceptable safety profile.

2. OBJECTIVES

2.1. PRIMARY OBJECTIVE

2.1.1.Phase I trial

Primary objective of the phase I trial is to establish the recommended phase II dose (RP2D), the maximum tolerated dose (MTD) evaluated on the first cycle (D1 to D28), the safety profile, and the Dose Limiting Toxicities (DLT) of Regorafenib when prescribed in association with Avelumab (no dose escalation for Avelumab) in patients treated for advanced digestive solid tumors.

2.1.2.Phase II trial

To investigate the antitumor activity of Regorafenib when prescribed in association with Avelumab, independently for 17 cohorts of patients : Colorectal cancer not MSI-H or MMR-deficient (cohorts A and A' with immune signature (based on low tumor-associated macrophages infiltrate level), GIST (Cohort B), Oesophageal or gastric carcinoma (Cohort C), Biliary tract cancer, hepatocellular carcinoma (Cohort D), Soft Tissue Sarcoma (STS – Cohort E), Radioiodine-Refractory Differentiated Thyroid Cancer (RR-DTC – Cohort F), Neuroendocrine gastroenteropancreatic tumors (GEP-NETs – Cohort G), Non-small cell lung cancer (Cohort H), Solid tumors (including Soft Tissue Sarcoma) with immune signature (TLS+) (Cohort I), urothelial cancer (Cohort J), HPV-associated cancer (Cohort K), triple negative breast cancer (L), TMB-high solid tumors (Cohort M), MSI-high solid tumors (Cohort N), non clear-cell renal carcinoma (Cohort O) and malignant pleural mesothelioma (Cohort P).

For cohorts A (Colorectal cancer not MSI-H or MMR-deficient [standard dose]), C (Oesophageal or gastric carcinoma), D (Biliary tract cancer, hepatocellular carcinoma), E (Soft Tissue Sarcoma [STS]), F (Radioiodine-Refractory Differentiated Thyroid Cancer), G (Neuroendocrine gastroenteropancreatic tumors)::, antitumor activity will be assessed in terms of objective response under treatment based on adapted RECIST 1.1 criteria after in-stream centralized radiological review (see endpoints, section 9).

For cohorts B (GIST), H (NSCLC), I (Solid tumors-TLS+), M (TMB-high solid tumors), N (MSI-high solid tumors), O (non clear-cell renal carcinoma) and P (malignant pleural mesothelioma), antitumor activity will be assessed in terms of 6-month progression-free rate (6-month PFR) based on RECIST 1.1 criteria after in-stream centralized radiological review (see endpoints, section 9).

For cohort A' with immune signature (based on low tumor-associated macrophages infiltrate level, antitumor activity will be assessed in terms of 4-month progression-free rate (4-month PFR) based on RECIST 1.1 criteria after in-stream centralized radiological review (see endpoints, section 9).

CONFIDENTIAL

For cohorts J, K and L, antitumor activity will be assessed in terms of disease control rate at 6-month (6-month DCR) based on RECIST 1.1 criteria after in-stream centralized radiological review (see endpoints, section 9).

2.2. SECONDARY OBJECTIVES

2.2.1.Phase I trial

- To evaluate preliminary signs of antitumor activity of Regorafenib prescribed in combination with Avelumab in terms of 6-month objective response, 6-month progression-free status, best overall response, objective response under treatment, growth modulation index (GMI), 1-year progression-free survival (PFS) and 1-year overall survival (OS).
- To describe the pharmacokinetics (PK) of Regorafenib when prescribed in combination with Avelumab.
- Biomarker study: To perform pharmacodynamic (PD)/mechanism of action (MOA) biomarkers analysis as well as predictive biomarkers analysis (levels of angiogenic and immunologic biomarkers in blood and tissue at baseline and different study time points).

2.2.2.Phase II trials

Independently for each trial:

- To evaluate the antitumor activity of Regorafenib when prescribed in association with Avelumab in terms of 6-month objective response, 6-month progression-free rate, best overall response, growth modulation index (GMI), 1-year progression-free survival (PFS) and 1-year overall survival (OS).
- To evaluate the Regorafenib safety profile of Regorafenib when prescribed in association with Avelumab.
- To perform pharmacodynamic (PD)/mechanism of action (MOA) biomarkers analysis as well as predictive biomarkers analysis (levels of angiogenic and immunologic biomarkers in blood/tissue at baseline and different study time points).
- For the cohort B: To evaluate the efficacy based on Choi criteria by independent radiologic review

3. STUDY DESIGN

3.1. OVERALL STUDY DESIGN

This is a multicenter, prospective open-labeled phase Ib trial based on a dose escalation study design (3+3 traditional design) assessing three dose levels of Regorafenib given in combination with Avelumab (no dose escalation for Avelumab) in patients with advanced digestive solid tumors followed by independent phase II trials in patients with advanced or metastatic solid and digestive tumors to evaluate the association of Regorafenib at the RP2D with Avelumab in 17 cohorts:

- Cohort A: Colorectal cancer not MSI-H or MMR-deficient (standard dose)
- Cohort B: GIST
- Cohort C: Oesophageal or gastric carcinoma
- Cohort D: Biliary tract cancer, hepatocellular carcinoma
- Cohort E: Soft Tissue Sarcoma (STS)
- Cohort F: Radioiodine-Refractory Differentiated Thyroid Cancer (RR-DTC)
- Cohort G: Neuroendocrine gastroenteropancreatic tumors (GEP-NETs)
- Cohort H: Non-Small cell lung cancer (NSCLC)
- Cohort I: Solid tumors (including Soft Tissue Sarcoma) with immune signature (TLS+).
- Cohort J: Urothelial cancer
- Cohort K: HPV-associated cancer
- Cohort L: Triple negative breast cancer
- Cohort M: TMB-high solid tumors
- Cohort N: MSI-high solid tumors
- Cohort O: Non clear-cell renal carcinoma
- Cohort P: Maligant pleural mesothelioma.

Moreover, we propose to evaluate in a phase II trial, the association of a low-dose of regorafenib (80 mg/day) with avelumab in patients with colorectal cancer not MSI-H or MMR-deficient, with immune signature (based on low tumor-associated macrophages infiltrate level:

- Cohort A': Colorectal cancer not MSI-H or MMR-deficient (low dose) with immune signature (based on low tumor-associated macrophages infiltrate level.

CONFIDENTIAL

3.2. PHASE I – DOSE ESCALATION PART

3.2.1. Definitions

Dose-limiting toxicity (DLT) is defined as an adverse event or laboratory abnormality that fulfills all the criteria below:

- Begins on the first 28 days of treatment
- Is considered to be at least possibly related to the study treatment
- Meets one of the criteria below:
  - Hematotoxicity:
    - Persistent grade 4 neutropenia lasting ≥7 days;
    - When a neutrophil count of <1000/mm3 and fever of ≥38.0°C is observed for >2 days;
    - Grade 4 thrombopenia or thrombopenia associated with a hemorrhage requiring platelet transfusion.
    - Grade 3 thrombocytopenia with bleeding.

  - Non-hematotoxicity:
    - ≥Grade 3 non-hematotoxicities are considered as DLTs with the following specifications:
    - ≥Grade 3 diarrhea, nausea, vomiting, and loss of appetite for ≥5 consecutive days (despite supportive therapy);
    - ≥Grade 3 electrolyte imbalance for ≥7 consecutive days (despite supportive therapy);
    - Grade 3 dermatologic toxicity (Hand foot syndrome reaction and non-life threatening events) for ≥7 consecutive days;
    - Grade 4 dermatologic toxicity of any duration;
    - ALT/AST increases 5-8 X ULN with concomitant bilirubin increase < 2 X ULN not resolving to < 5 X ULN within 7 days
    - ALT/AST increases 5-8 X ULN with concomitant bilirubin increase > 2 X ULN
    - ALT/AST increases > 8 X ULN regardless of concomitant bilirubin increase
    - Bilirubin increase > 3 X ULN not resolving to baseline within 7 days
    - ≥Grade 3 immune-related adverse events lasting for ≥8 consecutive days despite steroid therapy.

In addition, the following will be considered as DLT:

- Any other study drug related AE considered significant enough to be qualified as DLT in the opinion of the investigators after discussion with the sponsor
- Any drug-related AE leading to Regorafenib or Avelumab relative dose intensity lower or equal to 75% over the first treatment cycle.

Maximum tolerated dose (MTD): is defined as the highest dose at which no more than 1 in 6 of the patients in the cohort experienced a DLT in the first treatment cycle.

Recommended phase II dose (RP2D): will be identified by the steering committee based on the MTD, additional safety data (all cycles), PK data and PD data. Data from all cycles will be used to define the dose level to be recommended for further investigations in phase II

3.2.2. Treatment scheme

Regorafenib will be taken orally once daily for three weeks on/one week off, as appropriate for assigned dose level.
Avelumab will be administered intravenously, at fixed doses of 10 mg/kg, every two weeks, starting on Cycle 1 Day 15.
A treatment cycle consists of 4 weeks. Treatment may continue until disease progression or study discontinuation (withdrawal of consent, intercurrent illness, unacceptable adverse event or any other changes unacceptable for further treatment, etc.).

3.2.3. Dose levels

Dose escalation study assessing 3 doses level of Regorafenib given in combination with Avelumab (fixed dose).

Dose level	Regorafenib
-1	*80 mg once daily*
1 (starting)	120 mg once daily

CONFIDENTIAL

2	160 mg once daily

- The starting dose of Regorafenib is 120 mg once daily.
- The maximum dose of Regorafenib administered (160 mg once daily) will not be exceeded.
- No skipping of the dose will be allowed.
- For a given patient, dose will never be escalated.
- Patients will be allocated to 2 doses levels following a 3 + 3 design.
- A minimum of 3 patients and a maximum of 6 patients will be entered on each dose level.
- All 3 patients within a dose level will be observed during 28 days (the period of observation of DLTs) before accrual to the next higher dose level may begin.
- Dose escalation will proceed according to the following scheme:

**Number of patients with DLT at one dose level**	**Escalation Decision Rule**
0 out of 3	Enter 3 patients at the next dose level.
≥2	Dose escalation will be stopped. This dose level will be declared as the maximum administered dose (MAD). Three additional patients will be entered at the next lowest dose level if only 3 patients were treated previously at that dose.
1 out of 3	Enter at least 3 more patients at this dose level.<br>• If 0 of these 3 patients experience DLT, proceed to the next dose level.<br>• If 1 or more of this group suffer DLT, dose escalation will be stopped, and this dose is declared as the MAD. Three additional patients will be entered at the next lowest dose if only 3 patients were treated previously at that dose.
≤ 1 out of 6	If this is the highest dose level, this will be the maximum tolerated dose (MTD).<br>Else, proceed to the next dose level.

- As described above, the maximum administered dose (MAD) for Regorafenib is the dose in which ≥2/3 or ≥2/6 patients experience DLT.
- If the MAD for Regorafenib is seen at the starting dose level, then dose level "-1" will be the recommended dose. (Note: upon dose reduction, MAD criteria could be fulfilled but no tolerable combination may be found to continue into Phase II).
- The MTD for Regorafenib is defined as the highest dose at which no more than 1 in 6 of the patients in the cohort experienced a DLT during the period of observation of DLTs.
- The steering committee will meet before proceeding or not to each dose escalation. In addition, in case of discussion regarding the DLT status of a specific patient, the steering committee will be consulted to resolve any specific question regarding the DLT status (see section 12.1).
- The sponsor may decide to submit the cConclusions of the steering committee for the definition of MTD and the RP2D to an independent data monitoring committee (IDMC) before opening the phase II trials.

3.3. PHASE II TRIALS

Patients with advanced or metastatic solid tumors will be included in 10 independent single-arm open-label phase II trials:

- [A] Colorectal cancer not MSI-H or MMR-deficient (standard dose)
- [B] GIST
- [C] Oesophagal or gastric carcinoma
- [D] Biliary tract cancer, hepatocellular carcinoma
- [E] Soft Tissue Sarcoma (STS)
- [F] Radioiodine-Refractory Differentiated Thyroid Cancer (RR-DTC)
- [G] Neuroendocrine gastroenteropancreatic tumors (GEP-NETs).

The design of these single-arm phase II trials will follow an adaptative trial design (Bayesian approach, Section 10 for statistical considerations).

CONFIDENTIAL

The administered dose of Regorafenib will be the RP2D defined in the dose escalation part of the trial (see above).

- [H] Non-small cell lung cancer (NSCLC)
- [I] Solid tumors (including soft Tissue Sarcoma) with immune signature (TLS+).

The design of these single-arm phase II trials will follow an optimal two-stage Simon's design (see section 10 for statistical considerations).
The administered dose of Regorafenib will be the RP2D defined in the dose escalation part of the trial (see above).

- [A'] Colorectal cancer not MSI-H or MMR-deficient with immune signature (based on low tumor-associated macrophages infiltrate level.

The design of this single-arm phase II trial will follow an optimal two-stage Simon's design (see section 10 for statistical considerations).
The administered dose of Regorafenib will be at a fixed low-dose of 80 mg/day.

- [J]: Urothelial cancer
- [K]: HPV-associated cancer
- [L]: Triple negative breast cancer
- [M]: TMB-high solid tumors
- [N]: MSI-high solid tumors
- [O]: Non clear-cell renal carcinoma
- [P]: Malignant pleural mesothelioma.

The design of these single-arm phase II trials will follow an exact single-stage A'Hern design.
The administered dose of Regorafenib will be the RP2D defined in the dose escalation part of the trial.

3.4. PATIENT'S REPLACEMENT

See section 10.2

4. SELECTION OF PATIENTS

4.1. INCLUSION CRITERIA

1. Histology:
- Dose escalation part: histologically confirmed non MSI-H or MMR-deficient colorectal cancer, or GIST, or oesophageal or gastric carcinoma or hepatobiliary cancers,
- Phase II trials: histologically confirmed:
  - non MSI-H or MMR-deficient colorectal cancer (cohort A),
  - non MSI-H or MMR-deficient colorectal cancer with immune signature (cohort A'), i.e. low tumor-associated macrophages infiltrate level as determined by central review.

    Except if the low level of tumor-associated macrophages infiltrate levelhas been already confirmed by Biopathological platform at Bergonié Institute, the low level of tumor-associated macrophages infiltrate level should be confirmed by central review based on FFPE (Formalin-Fixed Paraffin-Embedded) tumor tissue sample (archived or newly obtained by biopsy for research purpose). Note that the level of tumor-associated macrophages infiltrate could be determined by central analysis if not available before.
  - or GIST (cohort B),: as recommended by INCa, patients must have diagnosis histologically confirmed by central review, except if it has been already confirmed by the RRePS Network.
  - or oesophageal or gastric carcinoma (cohort C)
  - or hepatobiliary cancers (cohort D),
  - or Soft Tissue Sarcoma (STS) (cohort E) : as recommended by INCa, patients must have diagnosis histologically confirmed by central review, except if it has been already confirmed by the RRePS Network
  - or Radioiodine-Refractory Differentiated Thyroid Cancer (RR-DTC) (cohort F)
  - or Neuroendocrine gastroenteropancreatic tumors (GEP-NETs) grade 2 and 3 (cohort G),
  - or Non-small cell lung cancer (cohort H),
  - or Solid tumors including soft-tissue sarcoma with immune signature (cohort I), i.e. presence of mature tertiary lymphoid structures (TLS).

CONFIDENTIAL

Except if presence of TLS have been already confirmed by Biopathological platform at Bergonié Institute, presence of TLS should be confirmed by central review based on FFPE (Formalin-Fixed Paraffin-Embedded) tumor tissue sample (archived or newly obtained by biopsy for research purpose). Note that the presence of TLS could be determined by central analysis if not available before.

- or urothelial cancer (cohort J)
- or HPV-associated cancer (cohort K) with molecular confirmation of p16 positive status.
- or triple negative breast cancer (cohort L)
- or TMB-high solid tumors (cohort M) with TMB-high status already known
- or MSI-high solid tumors (cohort N) with MSI-high status already known
- or Non clear-cell renal carcinoma (cohort O)
- or Malignant pleural mesothelioma (cohort P).

2. Advanced non resectable / metastatic disease,
3. Patients for which either there is no further established therapy that is known to provide clinical benefit,
4. Age ≥ 18 years,
5. ECOG, Performance status ≤ 1,
6. Measurable disease according to RECIST v1.1,
7. Life expectancy > 3 months,
8. Except for cohorts F (RR-DTC), ≥ 1 previous line (s) of systemic therapy
9. Adequate hematological, renal, metabolic and hepatic functions:
    a. Hemoglobin ≥ 9 g/dl (patients may have received prior red blood cell [RBC] transfusion, if clinically indicated); absolute neutrophil count (ANC) ≥ 1.5 x 10^9/l and platelet count ≥ 100 x 10^9/l,
    b. Alkaline phosphatase (AP), alanine aminotransferase (ALT) and aspartate aminotransferase (ASP) ≤ 2.5 x upper limit of normality (ULN) (≤ 5 in case of extensive skeletal involvement and/or liver metastasis for AP and ≤ 5 x ULN in case of liver metastasis for AST and ALT).
    c. Total bilirubin ≤ 1.5 x ULN.
    d. Albumin ≥ 25g/l.
    e. Calculated creatinine clearance (CrCl) ≥ 30 ml/min (according to Cockroft and Gault formula).
    f. Creatine phosphokinase (CPK) ≤ 2.5 x ULN
    g. INR or PT ≤1.5 x ULN
    h. aPTT ≤ 1.5 X ULN.
    i. Lipase ≤ 1.5 X ULN
    j. Cohort specific criteria: Patients with hepatocellular carcinoma must have a correct hepatocellular function, id est Child-Pugh A.
10. No prior or concurrent malignant disease diagnosed or treated in the last 2 years except for adequately treated in situ carcinoma of the cervix, basal or squamous skin cell carcinoma, or in situ transitional bladder cell carcinoma,
11. At least three weeks since last chemotherapy, immunotherapy or any other pharmacological treatment and/or radiotherapy,
12. Recovery to grade ≤ 1 from any adverse event (AE) derived from previous treatment, excluding alopecia of any grade and non-painful peripheral neuropathy grade ≤ 2 (according to the National Cancer Institute Common Terminology Criteria for Adverse Event (NCI-CTCAE, version 5.0)),
13. Women of childbearing potential must have a negative serum pregnancy test within 72 hours prior to receiving the first dose of study medication.
14. Both women and men must agree to use an highly effective method of contraception throughout the treatment period and for seven months (210 days) in WOCBP or four months (120 days) in men sexually active with WOCBP after discontinuation of treatment. Acceptable methods for contraception are described in section 7.4.1.
15. Voluntary signed and dated written informed consents prior to any specific study procedure,
16. Patients with a social security in compliance with the French law.
17. Documented disease progression (as per RECIST v1.1) before study entry.
    - For patient of cohorts E (STS) and cohort I (Solid tumors - TLS+): this progression will be confirmed by central review on the basis of two CT scan or MRI obtained at less than 6 months in the period of 12 months prior to inclusion.
    - For patient of cohort F (RR-DTC) : this progression will be confirmed by central review on the basis of two CT scan or MRI obtained at less than 12 months prior to inclusion.
18. *Cancelled (MSA6)*
19. For patients with non-small cell lung cancer (cohort H):

CONFIDENTIAL

- Subjects with histologically or cytologically confirmed diagnosis of non-squamous NSCLC
- Documented disease progression based on radiographic imaging, during or after a maximum of 2 lines of systemic treatment for locally/regionally advanced recurrent, Stage IIIb/Stage IV or metastatic disease. Two components of treatment must have been received in the same line or as separate lines of therapy
    - A maximum of 1 line of platinum-containing chemotherapy regimen in the metastatic setting, and
    - A maximum of 1 line of PD(L)1 mAb containing regimen, and
    - Patients must have received at least 4 months of PD(L1) mAb treatment.
- No EGFR, ALK, ROS1 positive tumor mutations
- Subjects with known BRAF molecular alterations must have had disease progression after receiving the locally available SoC treatment for the molecular alteration.

20. For patients with urothelial cancer (cohort J):
    - A maximum of 1 line of PD(L)1 mAb containing regimen, and
    - Patients must have received at least 4 months of PD(L1) mAb treatment.
21. For HPV-associated cancer (cohort K), TMB-high solid tumors (cohort M) MSI-high solid tumors (cohort N), Non clear-cell renal carcinoma (cohort O):
    - A maximum of 1 line of PD(L)1 mAb containing regimen, and
    - Patients must have received at least 4 months of PD(L1) in the case they received this treatment
22. For malignant pleural mesothelioma (Cohort P):
    - A maximum of 1 line of PD(L)1/CTLA-4 mAb containing regimen, and
    - Patients must have received at least 4 months of PD(L1)/CTLA-4 mAb treatment in the case they received this treatment
23. For triple-negative breast cancer patients (Cohort L)
    - A maximum of 1 line of PD(L)1 mAb containing regimen, and
    - Patients must have received at least 4 months of PD(L1) mAb treatment

Except if CPS<10, an anterior line of PD(L)1 mAb is not mandatory

24. For TMB-High cancer patients (Cohort M):
    - TMB-High is defined as TMB score > 16 mutations /megabase on tissue or blood sample

4.2. NON-INCLUSION CRITERIA

1. Previous treatment with Avelumab or Regorafenib,
2. For cohorts A to G and A': Has received prior therapy with an anti-PD-1, anti-PD-L1, anti-PD-L2, anti-CD137, or anti-Cytotoxic T-lymphocyte-associated antigen-4 (CTLA-4) antibody (including ipilimumab or any other antibody or drug specifically targeting T-cell co-stimulation or checkpoint pathways),
3. Evidence of progressive or symptomatic or newly diagnosed central nervous system (CNS) or leptomeningeal metastases.Participants with previously treated brain metastases may participate provided they are stable (without evidence of progression by imaging for at least 4 weeks before the first dose of study treatment and any neurologic symptoms have returned to baseline), have no evidence of new or enlarging brain metastases confirmed by repeat imaging, and have not required steroids for at least 7 days before study treatment.
4. Men or women of childbearing potential who are not using an effective method of contraception as previously described,
5. Participation to a study involving a medical or therapeutic intervention in the last 30 days,
6. Previous enrolment in the present study,
7. Patient unable to follow and comply with the study procedures because of any geographical, familial, social or psychological reasons,
8. Known hypersensitivity to any involved study drug or of its formulation components,
9. Active autoimmune disease that might deteriorate when receiving an immunostimulatory agent:
    a. Subjects with diabetes type I, vitiligo, psoriasis, hypo- or hyperthyroid disease not requiring immunosuppressive treatment are eligible
    b. Subjects requiring hormone replacement with corticosteroids are eligible if the steroids are administered only for the purpose of hormonal replacement and at doses ≤ 10 mg or 10 mg equivalent prednisone per day
    c. Administration of steroids through a route known to result in a minimal systemic exposure (topical, intranasal, intro-ocular, or inhalation) are acceptable

CONFIDENTIAL

10. Has a diagnosis of immunodeficiency or is receiving systemic steroid therapy or any other form of immunosuppressive therapy within 7 days prior to the first dose of trial treatment,
11. History of idiopathic pulmonary fibrosis (including pneumonitis), drug-induced pneumonitis, organizing pneumonia, or evidence of active pneumonitis on screening chest CT scan or interstitial lung disease with ongoing signs and symptoms at inclusion. History of radiation pneumonitis in the radiation field (fibrosis) is permitted,
12. Has known hepatitis B or hepatitis C active and/or treated by antiviral therapy,
13. Has a known history of Human Immunodeficiency Virus (HIV) (HIV1/2 antibodies) or known acquired immunodeficiency syndrome (AIDS)
14. Spot urine must not show 1+ or more protein in urine or the patient will require a repeat urine analysis. If repeat urinalysis shows 1+ protein or more, a 24-hour urine collection will be required and must show total protein excretion <1000 mg/24 hours,
15. Major surgical procedure or significant traumatic injury within 28 days before start of study medication,
16. Non-healing wound, non-healing ulcer, or non-healing bone fracture requiring orthopedic treatment,
17. Patients with evidence or history of any bleeding diathesis, irrespective of severity,
18. Any hemorrhage or bleeding event ≥ CTCAE Grade 3 within 4 weeks prior to the start of study medication,
19.Arterial or venous thrombotic or embolic events such as cerebrovascular accident (including transient ischemic attacks), deep vein thrombosis or pulmonary embolism within 6 months before the start of study medication (except for adequately treated catheter-related venous thrombosis occurring more than one month before the start of study medication),
20. Ongoing infection > Grade 2 as per NCI CTCAE v5.0,
21. Uncontrolled hypertension (Systolic blood pressure > 140 mmHg or diastolic pressure > 90 mmHg) despite optimal medical management,
22. Congestive heart failure ≥ New York Heart Association (NHYA) class 2,
23. Unstable angina (angina symptoms at rest), new-onset angina (begun within the last 3 months),
24. Myocardial infarction less than 6 months before start of study drug
25. Uncontrolled cardiac arrhythmias,
26. Pregnant or breast-feeding patients
27. Individuals deprived of liberty or placed under legal guardianship,
28. Prior organ transplantation, including allogeneic stem-cell transplantation,
29. Known alcohol or drug abuse
30. Vaccination within 4 weeks of the first dose of Avelumab and while on trial is prohibited except for administration of inactivated vaccines.
31. Patients with any condition that impairs their ability to swallow and retain tablets,
32. Other severe acute or chronic medical conditions including including immune inflammatory bowel disease, immune pneumonitis, pulmonary fibrosis or psychiatric conditions including recent (within the past year) or active suicidal ideation or behavior; or laboratory abnormalities that may increase the risk associated with study participation or study treatment administration or may interfere with the interpretation of study results and, in the judgment of the investigator, would make the patient inappropriate for entry into this study.
33. Patient with anti-Vitamine K therapy,
34. Suspected or known intraabdominal fistula.
35. For cohort H:
  - Received > 2 prior lines of therapy for NSCLC, including subjects with BRAF molecular alterations,
  - Subjects with known EGFR/ALK/ROS1 molecular alterations are excluded from participation in this study.

CONFIDENTIAL

5. STUDY PLAN

5.1. DURATION OF STUDY (WHOLE POPULATION)

The total duration of the study will be approximately 84 months, including about 72 months of active enrollment.
Planned start date (first patient on study): February 2018.
The planned study termination (clinical cutoff) will be 12 months after the last patient is included.

5.1.1.Phase I trial - Dose escalation part

Follow-up: 12 months
End of study occurs when all of the following criteria have been satisfied:
- The trial is closed to recruitment

AND

- All patients have disease progression or are no longer on study medication

  AND
- The last included patient has been followed for 12 months or is deceased

5.1.2.Phase II trials - Expansion cohorts

Follow-up: 12 months
End of study occurs when all of the following criteria have been satisfied:

- The trial is closed to recruitment

  AND
- All patients have disease progression or are no longer on study medication

  AND
- The last included patient has been followed for 12 months or is deceased

5.2. DEFINITIONS OF DURATION OF STUDY AND TREATMENT (PER PATIENT)

Patients will receive study treatment as long as it is considered to be in their best interest. Patients will be evaluated at scheduled visits in up to five study periods:

- **<u>Pre-treatment</u> (PRE TT):** from signature of informed consent to the first administration of study drugs.
- **<u>Treatment</u> (TT):** from the first administration of study drugs to treatment discontinuation
- **<u>Safety follow-up</u> (SFUP):** after treatment discontinuation, safety follow-up must be performed 30 days after the last treatment administration (toxicity assessment).
- **<u>Extended safety follow-up (ESFUP):</u>** given the potential risk for delayed immune-related toxicities, extended safety follow-up must be performed up to 90 days after the last dose of avelumab administration (ie. 60 days after SFUP). This visit may be performed either via a site visit or via a telephone call with subsequent site visit requested in case any concerns noted during the telephone call
- **<u>Follow-up (FUP):</u>**

After safety follow-up visit, patients who discontinue treatment without progression will be followed **every 12 weeks until**:

1. Disease progression,
2. Initiation of other antitumor therapy,
3. Death, or
4. The date of study termination, whichever occurs first.

After documented progression or start of a new antitumor therapy, patients will be **followed every 6 months until**:

1. Death, or
2. The date of study termination, whichever occurs first.

Patients will be considered to be **<u>on-study</u>** from the signature of the informed consent to the end of follow-up period.

Patients will be considered to be **<u>on-treatment</u>** for the duration of their treatment until 90 days after the last treatment administration, except if the patient starts a new antitumor therapy before this period.
Patients may withdraw their consent at any time; no further study activities will be conducted on them.

<u>Treatment discontinuation</u> occurs when an enrolled patient ceases to receive the study medication or starts a new antitumor therapy, regardless of the circumstances, and is defined as 30 days after the last dose of Regorafenib and/or Avelumab, unless the patient starts a new antitumor therapy, in which case the date of administration of this new antitumor therapy will be considered the date of treatment discontinuation. The primary reason for any discontinuation will be recorded on the patient's Case Report Form (CRF). If a patient discontinues treatment, every effort should be made to complete the scheduled assessments. Administration of the study treatment should be discontinued if this is considered to be in the best interest of the patient. More specifically, treatment will be discontinued due to any of the following reasons:

- o Disease progression,
- o Unacceptable toxicity,

CONFIDENTIAL

- o Intercurrent illness of sufficient magnitude to preclude safety continuation of the study,
- o Patient refusal and/or non compliance with study requirements,
- o Protocol deviation with an effect on the risk/benefit ratio of the clinical trial

Study discontinuation occurs when an enrolled patient ceases to participate in the study, regardless of the reason (as detailed under “Follow-up” in Section 5.7). Patients have the right to withdraw consent at any time; if this is the case, no further follow-up should be performed.
The date and reason for study discontinuation will be clearly documented on the patient’s CRF.

5.3. PROTOCOL DEVIATION

A protocol deviation is defined as any departure from what is described in the protocol of a clinical trial approved by an Independent Ethics Committee/Institutional Review Board (IEC/IRB) and Competent Authorities. Therefore, this applies to deviations related to patient inclusion and clinical procedures (e.g., assessments to be conducted or parameters to be determined), and also to other procedures described in the protocol that concern the Good Clinical Practice (GCP) guidelines or ethical issues (e.g., issues related to obtaining the patients’ Informed Consent, data reporting, the responsibilities of the investigator, etc.).
Deviations with no effects on the risk/benefit ratio of the clinical trial (such as minimal delays in assessments or visits) will be distinguished from those that might have an effect on this risk/benefit ratio, such as:

- ➢ Deviations that might affect the clinical trial objectives, such as those involving the inclusion/exclusion criteria (which could mean that the patient is not eligible for the trial) and those having an effect on patient evaluability.
- ➢ Deviations that might affect the patient’s well-being and/or safety, such as an incorrect dosing of the investigational medicinal product (plitidepsin) due to not following dose adjustment specifications or an incorrect preparation of the medication.
- ➢ Deviations related to the following of GCP guidelines as described in the protocol and regulations in force, such as deviations when obtaining the Informed Consent or not following the terms established for reporting serious adverse events, etc.

The investigators may suggest to the Sponsor the authorization of certain protocol deviations, especially if they are related to the inclusion/exclusion criteria or if they may have an effect on the evaluability of the patients. As a general rule, NO deviations that may have an effect on the risk/benefit ratio of the clinical trial will be authorized. Protocol deviations considered particularly relevant, which are related to ethical issues, fulfillment of GCP guidelines and trial procedures, will be notified to the pertinent IEC/IRB and, if pertinent, to the relevant authorities as established by local regulations.

CONFIDENTIAL

5.4. SCREENING EVALUATION

During the pre-treatment period, and once the patient has signed the Informed Consent Form, the Investigator will confirm the patient’s eligibility for the study by conducting the assessments detailed in Table below. The screening period is 28 days for cohorts A to H and 42 days maximum for cohorts I and A’.

Table. Screening assessments.

	**ASSESSMENT**	**TIME**
**1. History and clinical examination**	◆ Signed by the patient/legal representative Informed Consent Form	Prior to any specific study procedures<br><br>Within four weeks prior to Day 1 of cycle 1<br>D-28 to D-1
	◆ Medical history and baseline condition<br>◆ Complete physical examination<br>◆ Performance status (ECOG PS; see Appendix 1)<br>◆ Assessment of baseline signs and symptoms<br>◆ Concomitant treatments	Within two weeks prior to Day 1 of cycle 1 (+1 week tolerance) – D-14 to D-1

	ASSESSMENT	TIME
	◆ Vital signs: heart rate, blood pressure, body temperature, weight and height	Within 7 days prior to Day 1 of cycle 1 (+3 days tolerance) – D-7 to D-1
	◆ Demographic data<br>◆ Primary diagnostic and prior treatment/s data:<br>- Date of diagnosis of the primary disease<br>- Prior treatments (surgery, radiotherapy, chemotherapy, immunotherapy), specifying the date of best response and the time to progression	Within four weeks prior to Day 1 of cycle 1<br>D-28 to D-1
**2. Pathology**	◆ For cohorts B, E and I : central review to confirm diagnosis, except in case of diagnosis confirmed by RRePS Network<br>◆ For cohort I: Archived or newly obtained FFPE (Formalin-Fixed Paraffin-Embedded) block, in order to assess the presence of mature tertiary lymphoid structures (TLS), except if already confirmed by Biopathological platform at Bergonié Institute<br>◆ For cohort A': Archived or newly obtained FFPE (Formalin-Fixed Paraffin-Embedded) block, in order to assess the level of tumor-associated macrophages infiltrate, except if already confirmed by Biopathological platform at Bergonié Institute	Material sent within 7 days next to signed informed consent<br>Centrally sent for TLS status determination<br>Centrally sent for the level of tumor-associated macrophages infiltrate determination
**3. Laboratory tests**	◆ **Hematology**: neutrophils, lymphocytes, basophils, eosinophils, monocytes, haemoglobin, platelet count, red blood cell count, total white cell count.<br>◆ **Biochemistry**: Serum electrolytes (Na^+, K^+, Cl^-, P and Ca^{++}), liver function tests (AST, ALT, total bilirubin, GGT and AP), LDH, creatinine, glucose, total proteins, urea, albumin, CPK, lipase, amylase.<br>◆ **Thyroid test**:<br>▪ All cohorts : TSH,<br>▪ For cohort F only : Thyroglobulin and Antithyroglobulin antibody<br>◆ **Coagulation**: TP, INR, aPTT<br>◆ **Urinary**: blood, glucose, protein, gravity (dipstick)	Within 7 days prior to inclusion (+3 days tolerance). – D-7 to D-1
**4. Creatinine clearance**	◆ Calculated using the Cockroft formula (see Appendix 2)	Within 7 days prior to Day 1 of cycle 1 (+3 day tolerance) – D-7 to D-1
**5. Pregnancy test, if applicable**	◆ Measurement of serum human chorionic gonadotropin (HCG)	Within 72 hours prior to Day 1 of cycle 1
**6. ECG**	◆ Electrocardiogram	Within 7 days prior to Day 1 of cycle 1 (+3 days tolerance) – D-7 to D-1
**7. Tumor assessment**	◆ CT scan or MRI of all measurable sites, as per RECIST (Appendix 3)<br>◆ Brain scan for cohort F, as per standard management	Within four weeks prior to Day 1 of cycle 1<br>D-28 to D-1 (±7 days)
**8. Other tests**	◆ Intercurrent events, concomitant diseases and treatments.	Within two weeks prior to Day 1 of cycle 1.<br>– D-14 to D-1
**9. Biopsy**	◆ Only for consented patient (optional)	At baseline, within 7 days prior to Day 1.<br>– D-7 to D-1

CONFIDENTIAL

5.5. EVALUATIONS DURING TREATMENT

The following assessments will be done while the patient is on treatment.

Table. Evaluations during treatment

	ASSESSMENT	TIME
**1. Clinical examination**	• Complete physical examination<br>• Performance status (ECOG PS; see Appendix 1)	Day 1 of cycle 1<br>Repeat on Day 15 of Cycle 1<br>Repeat on Days 1 and 15 of cycle 2<br>Repeat on Day 1 of each subsequent cycle<br>At discontinuation visit
	• Vital signs: heart rate, blood pressure, body temperature and weight	Day 1 of cycle 1<br>Repeat on Day 15 of Cycle 1<br>Repeat on Days 1 and 15 of each subsequent cycle<br>At discontinuation visit
	• Assessment of signs and symptoms	Throughout the treatment period
	• Concomitant diseases and treatments	Throughout the treatment period
**2. Laboratory tests***	• **Hematology**: neutrophils, lymphocytes, basophils, eosinophils, monocytes, haemoglobin, platelet count, red blood cell count, total white cell count.	Up to 24 hours before Day 1 of cycle 1<br>Repeat on Days 8, 15 and 22 of Cycles 1 and 2<br>Thereafter, repeat before each Avelumab injection (ie. Day 1 and Day 15).<br>At discontinuation visit
	• **Biochemistry**: Serum electrolytes (Na^+, K^+, Cl^-, P and Ca^{++}), liver function tests (AST, ALT, total bilirubin, GGT and AP), LDH, creatinine, glucose, total proteins, urea, albumin, CPK, lipase, amylase.	Up to 24 hours before Day 1 of cycle 1<br>Repeat on Day 15 of Cycle 1<br>Repeat before each Avelumab injection (ie. Day 1 and Day 15).<br>At discontinuation visit<br>**Note that hepatic tests (AST, ALT, GGT and alkalin phosphatase) must be also performed at Days 8 and 22 of cycles 1 and 2**
	• **Thyroid test**:<br>▪ All cohorts : TSH,<br>▪ For cohort F only : Thyroglobulin and Antithyroglobulin antibody	Up to 24 hours before Day 1 of cycle 1<br>- All cohorts : TSH : Repeat on Day 15 of Cycle 1, repeat on Day 1 and Day 15 of cycle 2, then on Day 1 of each subsequent cycle, at discontinuation visit and safety follow-up visit<br>- For cohort F only : Thyroglobulin and Antithyroglobulin antibody : repeat on Day 1 every 2 cycles, at discontinuation visit and safety follow-up visit
	• **Coagulation**: TP, INR, aPTT<br>• **Urinary**: blood, glucose, protein, gravity (dipstick)	Up to 24 hours before Day 1 of cycle 1<br>Repeat on Day 15 of Cycle 1<br>Repeat on Day 1 and Day 15 of cycle 2.<br>Repeat on Day 1 of each subsequent cycle<br>At discontinuation visit
**3. Creatinine clearance**	• Calculated using the Cockroft formula (see Appendix 2)	Up to 24 hours before Day 1 of cycle 1<br>Repeat on Day 15 of Cycle 1<br>Repeat on Days 1 and 15 of cycle 2.<br>Repeat on Day 1 of each subsequent cycle<br>At discontinuation visit
**4. Pregnancy test, if applicable**	• **Measurement** of serum human chorionic gonadotropin (HCG)	To be repeated within 72 hours prior to the start of study treatment.<br>Will be repeated on day 1 of each cycle, in women with childbearing potential
**5. ECG**	• Electrocardiogram	If clinically indicated
**6. Tumor assessment**	• CT scan or MRI of all measurable sites, as per RECIST (see Appendix 3)	Tumor assessment must be repeated every eight weeks (±7 days) and at least four weeks after first documentation of objective response even if there are treatment delays.
**7. PK study**	• Blood samples	For phase I only, see section 17.1

CONFIDENTIAL

	ASSESSMENT	TIME
**8. Biomarker study**	◆ Blood samples<br>◆ Stool sample (optional)	See section 17.2<br>◆ Blood samples at C1D1, C2D1, C4D1, C6D1 and at progression (pre-dose).<br>◆ Stool sample for the microbiota only at C1D1 (pre-dose).
**9. Biopsy**	◆ Only for consented patient (optional)	At cycle 2 Day 1, after 4 weeks of treatment.<br>For more details, please refer to section 17.3
**10. AEs**	As per NCI-CTCAE, version 5.	Throughout the treatment period

*For all laboratory tests a window of 72 hours will be allowed.

5.6. EVALUATION AT SAFETY FOLLOW-UP VISIT

The safety follow-up will be scheduled 30 days (4 weeks) after the last treatment administration (a window of ±1 week is allowed). In case of the patients starts a new antitumor therapy, this visit must be done before the date of administration of this new antitumor therapy.

Regardless of the reason for discontinuation, the complete workup has to be done at the end-of-treatment visit. This will include the following assessments:

- Assessment of signs and symptoms.
- Complete physical examination.
- ECOG performance status.
- Vital signs [heart rate, blood pressure, and temperature].
- Hematology.
- Biochemistry.
- Thyroid function.
- Urinalysis.
- Calculated CrCl.
- Clinical and radiological tumor assessment (MRI) (except for patients with confirmed PD at discontinuation or who had started a new treatment).
- Intercurrent events and concomitant disease and treatments.
- Safety assessment (AEs).

Adverse events and SAE must be reported for 30 days after the last treatment administration or until the start of a new antitumor therapy, whichever occurs first. Note that even if the patient has started a new antitumor therapy, all AE and SAE suspected to be treatment-related will be reported until 30 days after the last treatment administration.

Morover, extended safety follow-up (ESFUP) must be performed up to 90 days after the last dose of avelumab administration (ie. 60 days after SFUP). This visit may be performed either via a site visit or via a telephone call with subsequent site visit requested in case any concerns noted during the telephone call. Beyond this period of time, only those SAEs suspected to be treatment-related will be reported (see Section 11).

5.7. FOLLOW-UP

- The date and reason of the study discontinuation will be recorded on the patient's CRF (see Section 5.2).
- Patients who discontinue treatment without PD will be followed every 12 weeks until progression, other antitumor therapy or death or until the date of study termination, whichever occurs first.
- After treatment discontinuation, patients will be followed every 6 months until death or until the date of study termination, whichever occurs first.
- Patients who withdraw consent will not be followed with any study procedures.

All AEs (including SAEs) suspected to be treatment-related or research-related will be followed-up until the events or their sequelae resolve or stabilize at a level acceptable to the Investigator and the Sponsor.

CONFIDENTIAL

6. REGISTRATION PROCEDURES

6.1. PHASE I TRIAL – ESCALATION PART

6.1.1. Screening

Upon signature of consent, screened patients will be entered on study centrally at the Institut Bergonié Coordinating Center by the Study Coordinator as described in a specific SOP provided by the Sponsor.

For GIST, if diagnosis has not been already reviewed by the RRePS Network, each site will send to Institut Bergonié within 7 days after the signature of informed consent:

- Pathology request form completed
- 10 unstained slides and/or preferable FFPE (Formalin-Fixed Paraffin-Embedded) block of specimen tumor sampling, obtained anytime during disease development
- Initial pathology report with patient code and date of birth (including macroscopic description) and pathology report of molecular biology if any.

6.1.2. Inclusion

Upon signature of consent, eligible patients will be entered in the study centrally at the Institut Bergonié Coordinating Center by the Study Coordinator as described in a specific SOP provided by the Sponsor.

Issues that would cause treatment delays should be discussed with the Principal Investigator. If a patient does not receive the protocol therapy following registration, the patient's registration on the study may be cancelled. The Study Coordinator should be notified of cancellations as soon as possible.

6.2. PHASE II TRIAL

6.2.1. Screening

Upon signature of consent, screened patients will be entered on study centrally at the Institut Bergonié Coordinating Center by the Study Coordinator as described in a specific SOP provided by the Sponsor.

For GIST (Cohort B) and cohorts STS (Cohorts E (STS) and I (Solid tumors STS-TLS+)), if diagnosis has not been already reviewed by the RRePS Network, each site will send to Bergonie Institute within 7 days after the signature of informed consent:

- Pathology request form completed
- 10 unstained slides and/or preferable FFPE (Formalin-Fixed Paraffin-Embedded) block of specimen tumor sampling, obtained anytime during disease development
- Initial pathology report with patient code and date of birth (including macroscopic description) and pathology report of molecular biology if any.

For cohorts I (Solid tumors) and A' (colorectal cancer) with immune signature, each site will send to Bergonie Institute within 7 days after the signature of informed consent:

- Pathology request form completed (specific Transfert Form)
- Archived or newly obtained FFPE (Formalin-Fixed Paraffin-Embedded) block, in order to assess the level of tumor-associated macrophages infiltrate for cohort A' and the presence of mature TLS in cohort I

Except if already confirmed by Biopathological platform at Bergonié Institute, the site will send the pathology request form completed (specific Transfert Form) and an anonymous report of analysis.

For cohorts E (STS), F (RR-DTC), and I (Solid tumors -TLS+), each site will send to Bergonie Institute for central review before registration:

- Cohorts E (STS) and I (Solid tumors -TLS+) : anonymized CD of CT-scan or MRI of two radiological assessments identical obtained at less than 6 months interval within the 12 months prior to inclusion
- Cohort F : anonymized CD of CT-scan or MRI of two radiological assessments identical obtained at less than 12 months interval prior to inclusion
- Baseline Clinical Subject Profile with the first shipment
- Radiological Referral Form

Once results of pathological (if applicable) and radiological review will be available, the Study Coordinator at Institut Bergonie will inform results by e-mail.

CONFIDENTIAL

6.2.2.Inclusion

Upon signature of consent, eligible patients will be entered in the study centrally at the Institut Bergonié Coordinating Center by the Study Coordinator as described in a specific SOP provided by the Sponsor.

Issues that would cause treatment delays should be discussed with the Principal Investigator. If a patient does not receive the protocol therapy following registration, the patient's registration on the study may be cancelled. The Study Coordinator should be notified of cancellations as soon as possible.

7. STUDY TREATMENTS

Sponsor will provide Regorafenib and Avelumab, with identifying labels that will include all the information required by local regulations.
Investigational medicinal products (IMPs) will have to be requested following pharmacy manual provided as a separate document and using appropriate forms provided by the Sponsor.
The study sites will have to ensure drug traceability at all times.

7.1. DESCRIPTION OF TREATMENT

For instructions regarding drug inventory, handling, reconstitution, dilution, storage, accountability and disposal, please refer to the IMP Investigator's Brochure and/or the more updated Summary of Product Characteristics (SPC), all provided as separate documents.

7.2. PHARMACEUTICAL INFORMATIONS

Product description	Galenic	Dosage	Route of administration	Storage	Supply
Avelumab	Solution	20 mg/ml	Intravenous	2°C ≤ temp ≤ 8°C	Yes
Regorafenib	Tablet	40 mg	Oral	≤ 25°C	Yes

7.3. ADMINISTRATION OF TREATMENT

Treatment will be administered on an outpatient basis: day-hospitalization for Avelumab infusions.

For the Phase I trial:

**Regimen Description**				
**Agent**	**Dose**	**Route**	**Schedule**	**Cycle length**
Avelumab	10 mg/kg	I.V.	Every 2 weeks<br>(start on Cycle 1 Day 15.<br>D1 and D15 of subsequent cycles)	**28 days<br>(4 weeks)**
Regorafenib	Doses as appropriate for assigned dose level.	Oral	Continuous, once daily<br>Day 1-Day 21	

For phases II trials and all cohorts, except cohort A':

**Regimen Description**				
**Agent**	**Dose**	**Route**	**Schedule**	**Cycle length**
Avelumab	10 mg/kg	I.V.	Every 2 weeks<br>(start on Cycle 1 Day 15.<br>D1 and D15 of subsequent cycles)	**28 days<br>(4 weeks)**
Regorafenib	Dose as defined in the phase I trial: 160 mg.	Oral	Continuous, once daily<br>Day 1-Day 21	

For phase II trial and cohort A':

**Regimen Description**				
**Agent**	**Dose**	**Route**	**Schedule**	**Cycle length**

CONFIDENTIAL

Avelumab	10 mg/kg	I.V.	Every 2 weeks<br>(start on Cycle 1 Day 15.<br>D1 and D15 of subsequent cycles)	**28 days (4 weeks)**
Regorafenib	80 mg	Oral	Continuous, once daily<br>Day 1-Day 21	

The patient treated by the combination of Regorafenib plus Avelumab will be requested to maintain a medication diary (Appendix 4) of each dose of medication. The medication diary will be returned to clinic staff at the end of each course.

Reported adverse events and potential risks are described in Section 11.

7.3.1.Regorafenib

Regorafenib will be taken orally on a three weeks on/one week off schedule, from Day 1 to Day 21. Regorafenib should be taken once daily at a fixed time each day. The tablets should be swallowed whole with water after a light meal that contains less than 600 calories and less than 30% fat. If a dose of Regorafenib is missed, then it should be taken on the same day as soon as the patient remembers. The patient should not take two doses on the same day to make up for a missed dose. In case of vomiting after Regorafenib administration, the patient should not take additional tablets.

7.3.2.Avelumab

Premedication

In order to mitigate infusion related reactions, pre-medicate patients with an antihistamine and with paracetamol (acetaminophen) prior to the first 4 infusions of avelumab. Premedication should be administered for subsequent avelumab doses based upon clinical judgment and presence/severity of prior infusion reactions.This regimen may be modified based on local treatment standards and guidelines as appropriate provided it does not include systemic corticosteroids.

Setting

Avelumab should be administered on cycle 1 Day 15 as a 1-hour intravenous (IV) infusion, repeated every two weeks thereafter (ie. Day 1 and Day 15 of each subsequent cycle, as a 1-hour intravenous infusion). Avelumab is administered diluted with 0.9% saline solution.
Avelumab should be administered in a setting that allows for immediate access to an intensive care unit or equivalent environment and administration of therapy for anaphylaxis, such as the ability to implement immediate resuscitation measures. Steroids (dexamethasone 10 mg), epinephrine (1:1,000 dilution), allergy medications (IV antihistamines), bronchodilators, or equivalents, and oxygen should be available for immediate access.

Observation period

Following Avelumab infusions, patients must be observed for 30 minutes post infusion for potential infusion related reactions.

Calculated dose

Avelumab dose will be calculated based on patient's weight at inclusion visit. The calculated dose will be used during the whole study except if the patient's body weight increases of at least 10% compared to baseline value. In such a case, study treatment dose will be recalculated based on new patient's weight.

7.4. RESTRICTION DURING THE STUDY

7.4.1.Contraception

Women of childbearing potential must be informed that Regorafenib may cause foetal harm.
Women of childbearing potential (WOCBP) and their partners should ensure highly effective contraception during treatment and for 7 months (210 days) in WOCBP or 4 months (120 days) in men sexually active with WOCBP after last dose of study drug(s).

Males, who are sexually active, must agree to the use of an highly effective method of contraception throughout the period of taking study treatment and for at least 4 months (120 days) after last dose of study drug.

CONFIDENTIAL

The following restrictions apply while the patient is receiving study treatment and for the specified times before and after:

– **Female patient of child-bearing potential:**

Females of childbearing potential who are sexually active with a non-sterilized male partner must use at least 1 **highly** effective method of contraception (table below) from the time of screening and must agree to continue using such precautions for 7 months (210 days) after the last dose of study treatment. Non-sterilised male partners of a female patient must use male condom plus spermicide throughout this period. Not engaging in sexual activity for the total duration of the drug treatment and the drug washout period is an acceptable practice; however, periodic abstinence, the rhythm method, and the withdrawal method are not acceptable methods of birth control. Female patients should also refrain from breastfeeding throughout this period.

– **Male patients with a female partner of childbearing potential:**

- Non-sterilized males who are sexually active with a female partner of childbearing potential must use a male condom plus spermicide from screening through 4 months (120 days) after receipt of the final dose of study treatment. Not engaging in sexual activity is an acceptable practice; however, occasional abstinence, the rhythm method, and the withdrawal method are not acceptable methods of contraception. Male patients should refrain from sperm donation throughout this period.
- Female partners (of childbearing potential) of male patients must also use a highly effective method of contraception throughout this period (Table below).

N.B Females of childbearing potential are defined as those who are not surgically sterile (ie, bilateral tubal ligation, bilateral oophorectomy, or complete hysterectomy) or post-menopausal. Non-sterilized male are defined as those who are not surgically sterile (ie, vasectomy).

Women will be considered post-menopausal if they have been amenorrheic for 12 months without an alternative medical cause. The following age-specific requirements apply:

- Women <50 years of age would be considered post-menopausal if they have been amenorrheic for 12 months or more following cessation of exogenous hormonal treatments and if they have luteinizing hormone and follicle-stimulating hormone levels in the post-menopausal range for the institution or underwent surgical sterilization (bilateral oophorectomy or hysterectomy).
- Women ≥50 years of age would be considered post-menopausal if they have been amenorrheic for 12 months or more following cessation of all exogenous hormonal treatments, had radiation-induced menopause with last menses >1 year ago, had chemotherapy-induced menopause with last menses >1 year ago, or underwent surgical sterilization (bilateral oophorectomy, bilateral salpingectomy or hysterectomy).

Highly effective methods of contraception, defined as one that results in a low failure rate (ie, less than 1% per year) when used consistently and correctly are described in table below. Note that some contraception methods are not considered highly effective (e.g. male or female condom with or without spermicide; female cap, diaphragm, or sponge with or without spermicide; non-copper containing intrauterine device; progestogen-only oral hormonal contraceptive pills where inhibition of ovulation is not the primary mode of action [excluding Cerazette/desogestrel which is considered highly effective]; and triphasic combined oral contraceptive pills).

CONFIDENTIAL

Highly Effective Methods of Contraception (<1% Failure Rate)

• Barrier/Intrauterine methods	• Hormonal Methods
• Copper T intrauterine device<br>• Levonorgestrel-releasing intrauterine system (e.g., Mirena®)[a]	• Implants: Etonogestrel-releasing implants: e.g. Implanon® or Norplant®<br>• Intravaginal: Ethinylestradiol/etonogestrel-releasing intravaginal devices: e.g. NuvaRing®<br>• Injection: Medroxyprogesterone injection: e.g. Depo-Provera®<br>• Combined Pill: Normal and low dose combined oral contraceptive pill<br>• Patch: Norelgestromin/ethinylestradiol-releasing transdermal system: e.g. Ortho Evra®<br>• Minipillc: Progesterone based oral contraceptive pill using desogestrel: Cerazette® is currently the only highly effective progesterone-based

[a] This is also considered a hormonal method

7.4.2.Food intakes restriction

Grapefruit and grapefruit juice are not allowed during the protocol treatment. Herbal medicine (e.g. St. John's wort) is not allowed during the protocol treatment.

7.5. GENERAL CONCOMITANT MEDICATION

7.5.1.Acceptable concomitant medication

All treatments that the investigator considers necessary for a subject's welfare may be administered at the discretion of the investigator in keeping with the community standards of medical care. All concomitant medications will be recorded on the case report form (CRF). If changes occur during the trial period, documentation of drug dosage, frequency, route, and date may also be included on the CRF.
All concomitant medications received within 28 days before the first dose of trial treatment and 30 days after the last dose of trial treatment should be recorded. Concomitant medications administered after 30 days after the last dose of trial treatment should be recorded for SAEs only.

7.5.2.Prohibited concomitant medication

Subjects are prohibited from receiving the following therapies during the Screening and Treatment Phase (including retreatment for post-complete response relapse) of this trial:
- Anti-cancer systemic chemotherapy or biological therapy
- Chemotherapy not specified in this protocol
- Immunotherapy not specified in this protocol
- Investigational agents other than Avelumab and Regorafenib
- Live vaccines within 30 days prior to the first dose of trial treatment and while participating in the trial. Examples of live vaccines include, but are not limited to, the following: measles, mumps, rubella, varicella, zoster, yellow fever, intranasal influenza, rabies, BCG and typhoid vaccine.
- Current use of immunosuppressive medication, EXCEPT for the following:
  - Intranasal, inhaled, topical steroids, or local steroid injection (e.g., intra-articular injection);
  - Systemic corticosteroids at physiologic doses ≤ 10 mg/day of prednisone or equivalent; Higher doses can be used for the management of immune related adverse events and/or skin rash (see appendix 8).
  - Steroids as premedication for hypersensitivity reactions (e.g., CT scan premedication).
- The use of oral anticoagulation therapy (i.e. warfarin, acenocoumarol, phenprocoumon) is not permitted since warfarin is metabolized by CYP2C9 and CYP3A4. Low molecular weight heparin and heparin are allowed.

Subjects who, in the assessment by the investigator, require the use of any of the aforementioned treatments for clinical management should be removed from the trial. Subjects may receive other medications that the investigator deems to be medically necessary.

The Exclusion Criteria describes other medications which are prohibited in this trial.

CONFIDENTIAL

There are no prohibited therapies during the Post-Treatment Follow-up Phase.

Short course Radiotherapy for symptoms or *palliative* treatment may be allowed after discussion with the sponsor.

7.5.3.Potential drug interaction

7.5.3.1. AVELUMAB

No formal drug interaction trials have been conducted with Avelumab in humans.

7.5.3.2. REGORAFENIB

Since Regorafenib is metabolized by CYP3A4:

- Concomitant use of strong inhibitors of CYP3A4 activity (e.g., clarithromycin, itraconazole, ketoconazole, posaconazole, telithromycin, and voriconazole) should be avoided
- Concomitant use of strong inducers of CYP3A4 activity (e.g., rifampicin, phenytoin, carbamazepine, phenobarbital) should be avoided.

The following website may be referenced for a more extensive list of P450 inhibitors and inducers: http://medicine.iupui.edu/clinpharm/ddis/main

CYP isoform-selective substrates: i*n vitro* data indicate that regorafenib and/or its metabolites inhibit the cytochromes CYP2C8, CYP2C9, CYP2B6 at concentrations which are achieved *in vivo* at steady state (peak plasma concentration of 8.1 micromolar). The *in vitro* inhibitory potency towards CYP3A4, CYP2D6 and CYP2C19 was less pronounced. Clinical data indicate that regorafenib may be given concomitantly with substrates of CYP2C8, CYP2C9, CYP3A4, and CYP2C19 without a clinically meaningful drug interaction.

No interaction in vitro was observed for regorafenib and its metabolites with CYP1A2, CYP2A6, and CYP2E1. Regorafenib exhibited no inductive potential on major cytochrome P450 (CYP) isoforms (e.g., CYP1A2 and 3A4).

Since Regorafenib is metabolized by UGT1A9:

Co-administration of strong UGT1A9 inhibitors (e.g. mefenamic acid, diflunisal, and niflumic acid) during Regorafenib treatment should be avoided, as their influence on the steady-state exposure of Regorafenib and its metabolites has not been studied.

Based on in vitro data regorafenib as well as its active metabolite M-2 inhibits glucuronidation mediated by UGT1A1 and UGT1A9, whereas M-5 only inhibits UGT1A1 at concentrations which are achieved in vivo at steady state.

Co-administration of Regorafenib with UGT1A1 and UGT1A9 substrates may increase the systemic exposure to these substrates.

Regorafenib was no substrate for human P-gp, BCRP, OATP1B1, OATP1B3, OAT1, OAT3, and OCT2. Regorafenib exhibited no inhibitory potential toward OATP1B1, OATP1B3, OAT1, OAT3, and OCT2, but inhibited P-gp (without clinical relevance) and BCRP.

In vitro data indicate that the active metabolites M-2 and M-5 are substrates for BCRP and P-glycoprotein.

Co-administration of Regorafenib may increase the plasma concentrations of BCRP substrates (e.g., methotrexate, rosuvastatin, fluvastatin, atorvastatin). Therefore, it is recommended to monitor patients closely for signs and symptoms of increased exposure to BCRP substrates.

Co-administration with neomycin may result in a decreased efficacy of regorafenib. The effect of other antibiotics was not studied.

The concentration-time profile indicates that Regorafenib and its metabolites may undergo enterohepatic circulation. Bile salt sequestering agents such as cholestyramine and cholestagel may interact with Regorafenib by forming insoluble complexes which may impact absorption (or reabsorption), thus resulting in potentially decreased exposure. The clinical significance of these potential interactions is unknown, but may result in a decreased efficacy of Regorafenib.

Refer to the appendix 9 and to most updated Investigator Brochure for more information.

CONFIDENTIAL

7.6. DOSING DELAYS/DOSE MODIFICATIONS AND ADVERSE EVENT MANAGEMENT

7.6.1.Immune related adverse events (irAE)

IrAR may be defined as an adverse event of unknown etiology, associated with drug exposure and is consistent with an immune phenomenon.
irAEs may be predicted based on the nature of the Avelumab compound, its mechanism of action, and reported experience with immunotherapies that have a similar mechanism of action. Special attention should be paid to AEs that may be suggestive of potential irAEs. An irAE can occur shortly after the first dose or several months after the last dose of treatment.
If an irAE is suspected, efforts should be made to rule out neoplastic, infectious, metabolic, toxin or other etiologic causes prior to labeling an adverse event as an irAE.

Restart Avelumab if the adverse reaction remains at Grade 1 or less. If another episode of a severe adverse reaction occurs, permanently discontinue Avelumab.

Patient requiring 2 or more consecutive cancellations of Avelumab injection should permanently discontinue avelumab. Note that further treatment by regorafenib monotherapy will be allowed until disease progression, unacceptable toxicity, etc. (see section 3.2.2 for more details).

For management, refer to the appendix 8 and to most updated Investigator Brochure.

7.6.2.Management of Adverse Events

Any toxicity observed during the course of the study could be managed by interruption and/ or dose reduction of the dose if deemed appropriate by the Investigator. Dose adjustments are to be made according to the greatest degree of toxicity (please refer to the IMP Investigator's Brochure), and in accordance with table below:

**Level**	***-1***	**1**	**2**
Avelumab	*10 mg/kg*	10 mg/kg	10 mg/kg
Regorafenib	*80 mg x 1*	120 mg x 1	160 mg x 1

Adverse events will be graded using the NCI Common Terminology Criteria for Adverse Events Version (CTCAE) v5.0.

The following guidelines outline dose adjustments for the most frequent of these toxic effects. If any severe toxicity occurs it should be referred to the principal investigator in order to decide management in the best interest of the patient. If a patient experiences several adverse events with conflicting recommendations, please use the dose modification recommendation that reduces the dose to the lowest level.

Once a dose has been reduced, it will in no case be increased again.

For all cohorts except cohort [A'], patients requiring a delay of > 4 weeks OR > one dose reduction (of Regorafenib) if RP2D is 120mg of Regorafenib and > 2 dose reductions if RP2D is 160mg of Regorafenib should permanently discontinue regorafenib. Note that further treatment by avelumab monotherapy will be allowed until disease progression, unacceptable toxicity, etc. (see section 3.2.2 for more details).

For cohort [A'], regorafenib should be interrupted and no dose reduction are allowed. Patients requiring a delay of > 4 weeks should permanently discontinue regorafenib. Note that further treatment by avelumab monotherapy will be allowed until disease progression, unacceptable toxicity, etc. (see section 3.2.2 for more details).

Patient should go off study if regorafenib has been permanently discontinued before first dose of avelumab.

Please refer to Appendix 7 and 8 and to most updated Investigator Brochure for management of toxicities.

CONFIDENTIAL

Management of Infusion-Related Reactions

NCI-CTCAE Grade	Treatment Modification for Avelumab
**Grade 1 - mild**<br>Mild transient reaction; infusion interruption not indicated; intervention not indicated	Decrease the Avelumab infusion rate by 50% and monitor closely for any worsening
**Grade 2 - moderate**<br>Therapy or infusion interruption indicated but responds promptly to symptomatic treatment (for example , antihistamines, NSAIDs, narcotics, Iv fluids) ; prophylactic medications indicated for ≤ 24 h	Temporarily discontinue Avelumab infusion<br>Resume infusion at 50% of previous rate once infusion-related reaction has resolved or decreased to at least Grade 1 in severity , and monitor closely for any worsening
**Grade 3 or Grade 4 - severe or life-threatening**<br>Grade 3: Prolonged (for example, not rapidly responsive to symptomatic medication and/or brief interruption of infusion); recurrence of symptoms following initial improvement; hospitalization indicated for clinical sequelae<br>Grade 4: Life-threatening consequences; urgent intervention indicated	Stop the Avelumab infusion immediately and disconnect infusion tubing from the subject<br>Subjects have to be withdrawn immediately from Avelumab treatment and must not receive any further Avelumab treatment

7.7. PACKAGING AND LABELING

The following information will appear on the labels:

- Name of the Sponsor.
- Study number/Patient number.
- Dosage and route of administration.
- Quantity or contents of container.
- Batch number/packaging number.
- Expiration date and storage conditions.
- Local legal information, as appropriate.

7.8. SUPPLIES AND DRUG ACCOUNTABILITY

Regorafenib and Avelumab will be supplied to investigators.
For ordering study drugs, please confer process of this study and pharmaceutical procedure.
Proper drug accountability will be done by the clinical trial monitor. Each study site will keep records to allow a comparison of quantities of drug received and used at each site. The Investigator at each study site will be the person ultimately responsible for drug accountability at the site.
All unused drug supplied by the Sponsor will be properly destroyed at the study site, at the end of the study. The documentation of this procedure must be provided to the clinical trial monitor.

7.9. TREATMENT COMPLIANCE

The Investigator is responsible for supervising compliance with the instructions described in this study protocol.

8. STUDY EVALUATIONS

Study evaluations aim to assess:

- Diagnosis
- Efficacy
- Safety

CONFIDENTIAL

8.1. CENTRAL REVIEW FOR DIAGNOSIS, IMMUNE SIGNATURE AND PROGRESSIVE DISEASE

8.1.1.Diagnosis

8.1.1.1. DIAGNOSIS OF GIST (COHORT B) AND STS (COHORTS E (STS) AND I (SOLID TUMORS STS-TLS+))

If diagnosis was not confirmed by the RRePS Network, pathological central review will be performed to confirm histological diagnosis of sarcoma by Dr Le Loarer and collaborators, Department of Pathology, Institut Bergonié, Bordeaux, France. The reviewer will assess pathological diagnosis; document the results on the 'Pathological request form' response completed and sign this form.
Every discrepancy will be discussed between referral investigator, Pr Coindre or collaborators and the Sponsor, until a final decision is reached. Patients with diagnosis different from GIST or STS will be considered ineligible and will not be included in the study.

8.1.1.2. PATHOLOGICAL SPECIMEN SAMPLING NECESSARY FOR CENTRAL REVIEW

For a gross description and diagnostic information concerning pathological specimens, reference to "Recommendations for reporting soft tissue sarcomas" is strongly advised (Recommendations, 1999). Available tumor samples obtained at diagnosis or at relapse, as unstained slides (10), and/or preferable paraffin-embedded tumor blocks (one or two) are mandatory for central review.

8.1.1.3. PATHOLOGICAL PROCESS SCHEDULE AND IMPLEMENTATION (NOT APPLICABLE IF DIAGNOSIS REVIEWED IN RREPS NETWORK)

Each site will send to Institut Bergonié within 7 days after the signature of informed consent:
- Pathology request form completed
- 10 unstained slides and/or preferable FFPE (Formalin-Fixed Paraffin-Embedded) block of specimen tumor sampling, obtained anytime during disease development
- Initial pathology report with patient code and date of birth (including macroscopic description) and pathology report of molecular biology if any.

All material must be sent to:

**Institut Bergonie - Service Pathologie**<br>**Protocole REGOMUNE**<br>229 cours de l'Argonne - 33076 Bordeaux Cedex, France

8.1.2. Immune signature - Central review before inclusion

8.1.2.1. IMMUNE SIGNATURE FOR SOLID TUMOR (TLS) (COHORT I) AND COLORECTAL CANCER (COHORT A')

Centralized analysis for immune signature will be performed to determine the status of mature tertiary lymphoid structures (TLS) (Cohort I) and the level of tumor-associated macrophages infiltrate (Cohort A') at inclusion time.
This will be performed by centrally reviewing, except if already confirmed by Biopathological platform at Bergonié Institute (cf §6.2.1).

8.1.2.2. PATHOLOGICAL SPECIMEN SAMPLING NECESSARY FOR CENTRAL REVIEW

Archived or newly obtained FFPE (Formalin-Fixed Paraffin-Embedded) block are mandatory for central review.

8.1.2.3. PATHOLOGICAL PROCESS SCHEDULE AND IMPLEMENTATION

Each site will send to Institut Bergonié within 7 days after the signature of informed consent:
- Pathology request form completed (specific Transfert Form)
- Archived or newly obtained FFPE (Formalin-Fixed Paraffin-Embedded) block, in order to assess the presence of mature TLS in cohort I and the level of tumor-associated macrophages infiltrate in cohort A'

All material must be sent to:

**Institut Bergonie - Département de Biopathologie – Recherche Clinique**<br>**Protocole REGOMUNE**<br>229 cours de l'Argonne - 33076 Bordeaux Cedex, France

CONFIDENTIAL

8.1.3. Diagnosis of progressive disease – Central review before inclusion – cohorts E (STS), F (RR-DTC) and I (Solid tumors-TLS+)

8.1.3.1. Centralized radiological review

Centralized radiological review will be performed to confirm progressive disease status at inclusion time. This will be performed by centrally reviewing:

- For Cohorts E (STS): two CT scans or MRI obtained prior to inclusion (obtained at an interval less than 6 months in the period of 12 months prior to inclusion).
- For Cohort F (RR-DTC): two CT scans or MRI obtained prior to inclusion (obtained at an interval less than 12 months prior to inclusion).
- For Cohorts I (Solid tumors-TLS+): two CT scans or MRI obtained prior to inclusion (obtained at an interval less than 6 months in the period of 12 months prior to inclusion).

8.1.3.2. General procedure

Review process will be centralized at Institut Bergonié and will be performed by one radiologist expert. The results of the centralized review will be used for the diagnosis for progressive disease.

8.1.3.3. Review process schedule and practical implementation

With regards to inclusion scan, the progressive disease status at baseline must be confirmed by central review. Within 7 days after the signature of informed consent, each site will send two-imaging CD as described in a specific SOP given by the Sponsor.

8.2. EFFICACY – FOR PATIENTS INCLUDED IN PHASE II TRIALS ONLY

The antitumor activity of Regorafenib given in association with Avelumab will be evaluated in terms of Objective response rate (ORR) under treatment, 6-month Non-progression rate (NPR), best overall response, 1-year Progression-free survival (PFS), 1-year Overall survival (OS). Objective response, Non-progression and best objective response are defined as per the Response Evaluation Criteria in Solid Tumors (RECIST v1.1).

8.2.1. Assessing Objective Tumor Response (RECIST v1.1)

- A comprehensive workup will be performed at baseline, and every 8 weeks.
- Whenever response criteria are met, the appropriate imaging tests should be repeated at least four weeks later in order to confirm the response.
- The same method will be used to evaluate each identified lesion both at baseline and throughout the study.
- Treatment will be administered as long as no disease progression or unacceptable toxicity is found, or as long as no other reasons for treatment discontinuation are met.
- Assessment of efficacy will be essentially based on a set of measurable lesions identified at baseline as target lesions and followed until disease progression and following the RECIST v1.1 criteria (Einsenhauer, 2009).

8.2.2. Centralized Radiological Review (Institut Bergonié)

8.2.2.1. General procedure

Centralized radiological review will be performed to confirm disease status under treatment in comparison with baseline, Week#8, Week #16, Week#24 and every eight weeks thereafter. For patients included in the reference center, CT scan will be initially read by a radiologist who differs from the expert. Review process will be centralized at Institut Bergonié and will be performed by a radiologist expert in solid and digestive tumors.
In case of discordance between the local radiologist and the expert reviewer, the judgment provided by the expert reviewer will be retained to continue or stop the treatment: treatment must be continued until central review response is available.

8.2.2.2. Review process Schedule

All tumor evaluations will be sent as soon as there were available.
Patient’s information must be recorded on a provided imaging CD.

8.2.2.3. Practical implementation

For each shipment, each media should be accompanied by the Radiological Forms provided by the sponsor.
All CDs must be sent to the coordinating clinical research assistant.

CONFIDENTIAL

8.3. SAFETY

Patients will be evaluable for safety if they have received at least one treatment administration. Safety will be evaluated using clinical examinations, which will comprise vital signs analysis, clinical assessment of AEs, changes in laboratory parameters (hematological and biochemical, including liver function tests) and any other analyses that may be considered necessary. Safety profile will be continuously followed during treatment up to 90 days after the last treatment administration or until the start of a new antitumor therapy, whichever occurs first. All AEs will be classified according to the NCI-CTCAE, version 5.0.

9. STUDY ENDPOINTS

9.1. PHASE I TRIAL

9.1.1.Primary endpoint

- Primary objective of the phase I trial is to establish the recommended phase II dose (RP2D), the maximum tolerated dose (MTD) evaluated on the first cycle (D1 to D28), the safety profile, and the dose limiting toxicities (DLT) of Regorafenib when prescribed in association with Avelumab (no dose escalation for Avelumab) in patients treated for advanced digestive solid tumors.
- Toxicities will be graded using the common toxicity criteria from the NC-CTCAE v5.0.
- Incidence rate of DLT at each dose level on cycle 1 will be reported.
- Dose limiting toxicities are defned as follows:

Dose-limiting toxicity (DLT) is defined as an adverse event or laboratory abnormality that fulfills all the criteria below:

- Begins on the first 28 days of treatment
- Is considered to be at least possibly related to the study treatment
- Meets one of the criteria below:
  - Hematotoxicity:
    - Persistent grade 4 neutropenia lasting ≥7 days;
    - When a neutrophil count of <1000/mm3 and fever of ≥38.0°C is observed for >2 days;
    - Grade 4 thrombopenia or thrombopenia associated with a hemorrhage requiring platelet transfusion.
    - Grade 3 thrombocytopenia with bleeding.
  - Non-hematotoxicity:
    - ≥Grade 3 non-hematotoxicities are considered as DLTs with the following specifications:
    - ≥Grade 3 diarrhea, nausea, vomiting, and loss of appetite for ≥5 consecutive days (despite supportive therapy);
    - ≥Grade 3 electrolyte imbalance for ≥7 consecutive days (despite supportive therapy);
    - Grade 3 dermatologic toxicity(HFSR and non-life threatening events) for ≥7 consecutive days;
    - Grade 4 dermatologic toxicity of any duration;
    - ALT/AST increases 5-8 X ULN with concomitant bilirubin increase < 2 X ULN not resolving to < 5 X ULN within 7 days
    - ALT/AST increases 5-8 X ULN with concomitant bilirubin increase > 2 X ULN
    - ALT/AST increases > 8 X ULN regardless of concomitant bilirubin increase
    - Bilirubin increase > 3 X ULN not resolving to baseline within 7 days
    - ≥Grade 3 immune-related adverse events lasting for ≥8 consecutive days despite steroid therapy.

In addition, the following will be considered as DLT:

- Any other study drug related AE considered significant enough to be qualified as DLT in the opinion of the investigators after discussion with the sponsor
- Any drug-related AE leading to Regorafenib or Avelumab relative dose intensity lower or equal to 75% over the first treatment cycle.

CONFIDENTIAL

9.1.2. Secondary endpoints

- Preliminary signs of antitumor activity in terms of:
  - Best overall response is defined as the best response recorded from the start of the study treatment until the end of treatment taking into account any requirement for confirmation as per RECIST v1.1 criteria (appendix 3). Following RECIST v1.1 recommendations:
    - The best overall response is determined once all the data for the patient is known.
    - Claimed responses will have to be confirmed at least 4 weeks later to ensure responses identified are not the result of measurement errors.
  - Objective response rate (ORR) is defined as the proportion of patients with complete response or partial response according to RECIST v1.1 criteria (appendix 3). ORR under treatment and 6-month ORR will be reported. ORR under treatment is recorded from study treatment initiation until the end of treatment. Following RECIST v1.1 recommendations:
    - ORR under treatment is determined once all the data for the patient is known.
    - Claimed responses will have to be confirmed at least 4 weeks later to ensure responses identified are not the result of measurement errors.
  - Progression-free rate (PFR) is defined as the proportion of patients with complete response, partial response or stable disease more than 24 weeks as defined as per RECIST v1.1 criteria (appendix 3). 6-month PFR will be reported. Following RECIST v1.1 recommendations, claimed responses will have to be confirmed at least 4 weeks later to ensure responses identified are not the result of measurement errors.
  - Growth modulation index (GMI): GMI will be defined for each patient as the ratio of its PFS on Regorafenib + Avelumab treatment to its PFS on the previous line of therapy. This method accounts for inter-patient variability, the patient serving as his/her own control and implies by the natural history of the disease that the PFS tends to become shorter in successive lines of therapy. It is thought that an anti-cancer agent should be considered effective if the GMI is greater than 1.3
  - Progression-free survival (PFS) is defined as the time from study treatment initiation to the first occurrence of disease progression or death (of any cause), whichever occurs first. 1-year PFS rate and median PFS will be reported.
  - Overall Survival (OS) is defined as the time from study treatment initiation to death (of any cause). 1-year OS rate and median OS will be reported.
- PK measurements expressed as AUC, half-life and concentration peak for Regorafenib
- Pharmacodynamic activity: Predictive biomarkers analysis and pharmacodynamic (PD)/mechanism of action (MOA) in blood (levels of angiogenic and immunologic biomarkers in blood at baseline and different study time points), potentially including but not limited to:
  - Serum/plasma cytokines levels (ELISA)
  - Treg, CD4+, CD8+ and DR lymphocytes subpopulations (flow cytometry)
  - Archived tumor tissue will be collected for assessment of tumor VEGFR, PDGFR, HIF1alpha expression and lymphocytes, TAM and MDSC tumor infiltrates (IHC)
  - In addition, for all patients, optional biopsy at baseline and after 4 weeks of treatment will be proposed for mechanisms of action documentation: tumor VEGFR, PDGFR, HIF1alpha expression as well as PD-L1/PD1, lymphocytes, TAM, MDSC tumor infiltrates (IHC) and mutational status.

9.2. PHASE II TRIALS

9.2.1. Primary endpoint

- Primary objective is to investigate the antitumor activity of Regorafenib when prescribed in association with Avelumab, independently for 17 cohorts of patients : Colorectal cancer not MSI-H or MMR-deficient (cohorts A and A' with immune signature (based on low tumor-associated macrophages infiltrate level), GIST (Cohort B), Oesophageal or gastric carcinoma (cohort C), Biliary tract cancer, hepatocellular carcinoma (Cohort D), Soft Tissue Sarcoma (STS – Cohort E), Radioiodine-Refractory Differentiated Thyroid Cancer (RR-DTC – Cohort F), Neuroendocrine gastroenteropancreatic tumors (GEP-NETs – Cohort G), Non-small cell lung cancer (Cohort H), Solid tumors (including Soft Tissue Sarcoma) with immune signature (TLS+) (Cohort I), urothelial cancer (Cohort J), HPV-associated cancer (Cohort K), triple negative breast cancer (L), TMB-high solid tumors (Cohort M), MSI-high solid tumors (Cohort N), non clear-cell renal carcinoma (Cohort O) and malignant pleural mesothelioma (Cohort P).

- For cohorts A, as well as C to G, antitumor activity will be assessed in terms of objective response under treatment based on adapted RECIST 1.1 criteria, and after in-stream centralized radiological review:

CONFIDENTIAL

- Objective response under treatment is defined as patients with confirmed complete response (CR) or partial response, as per RECIST v1.1 criteria, observed during treatment with the investigational product(s).
    - As per RECIST v1.1 criteria, to be considered as “confirmed”, complete and partial responses will have to be confirmed at least 4 weeks later to ensure responses identified are not the result of measurement errors. If the new imaging to confirm response is not performed after 4 weeks, complete or partial responses will be considered as unconfirmed responses.
    - Objective response rate (ORR) under treatment is defined as the proportion of patients with objective response (confirmed or unconfirmed) under treatment based on adapted RECIST 1.1 (taking into account “unconfirmed “responses).

- For cohorts B (GIST), H (NSCLC), I (Solid tumors -TLS+), M (TMB-high solid tumors), N (MSI-high solid tumors), O (non clear-cell renal carcinoma) and P (malignant pleural mesothelioma), antitumor activity will be assessed in terms of 6-month progression-free rate (6-month PFR) based on RECIST 1.1 criteria after in-stream centralized radiological review. 6-month PFR is defined as the proportion of patients with progression-free status at 6 months. Progression-free status is defined as complete response (confirmed or unconfirmed), partial response (confirmed or unconfirmed) or stable disease more than 24 weeks as defined as per RECIST v1.1 criteria.

- For cohort A’ with immune signature (based on low tumor-associated macrophages infiltrate level) antitumor activity will be assessed in terms of 4-month progression-free rate (4-month PFR) based on RECIST 1.1 criteria after in-stream centralized radiological review. 4-month PFR is defined as the proportion of patients with progression-free status at 4 months. Progression-free status is defined as complete response (confirmed or unconfirmed), partial response (confirmed or unconfirmed) or stable disease more than 24 weeks as defined as per RECIST v1.1 criteria.

- For cohorts J (Urothelial cancer), K (HPV-associated cancer) and L (Triple negative breast cancer): antitumor activity will be assessed in terms of disease control rate at 6-month (6-month DCR rate) based on RECIST 1.1 criteria after in-stream centralized radiological review. 6-month DCR rate is defined as as the proportion of participants with confirmed complete response (CR), unconfirmed complete response (CRu), confirmed partial response (PR) or unconfirmed partial response (PRu) or stable disease (SD), as per adapted RECIST v1.1, observed within 24 weeks of treatment onset (while treated with the investigational product).

9.2.2.Secondary endpoints

- Best overall response is defined as the best response across all time points (RECIST v1.1). Following RECIST v1.1 recommendations:
    - The best overall response is determined once all the data for the patient is known.
    - The best overall response will be classified as confirmed complete response (CR), unconfirmed complete response (CRu), confirmed partial response (PR), unconfirmed partial response (PRu), stable disease or progressive disease, as per RECIST v1.1 criteria.
    - As per RECIST v1.1 criteria, to be considered as “confirmed”, complete and partial responses will have to be confirmed at least 4 weeks later to ensure responses identified are not the result of measurement errors.

- Objective response rate at 6 months (6-month ORR) is defined as the proportion of patients with objective response (confirmed or unconfirmed) at 6 months.

- Progression-free status is defined as complete response (confirmed or unconfirmed), partial response (confirmed or unconfirmed) or stable disease more than 24 weeks as defined as per RECIST v1.1 criteria (appendix 3). 6-month progression-free rate (6-month PFR) is defined as the proportion of patients with progression-free status at 6 months. 4-month progression-free rate (4-month PFR) is defined as the proportion of patients with progression-free status at 4 months.

- Growth modulation index (GMI): GMI is defined for each patient as the ratio of its PFS on Regorafenib + Avelumab treatment to its PFS on the previous line of therapy. This method accounts for inter-patient variability, the patient serving as his/her own control and implies by the natural history of the disease that the PFS tends to become shorter in successive lines of therapy. It is thought that an anti-cancer agent should be considered effective if the GMI is greater than 1.3

- Progression-free survival (PFS) is defined as the time from study treatment initiation to the first occurrence of disease progression or death (of any cause), whichever occurs first. 1-year PFS rate and median PFS will be reported.

CONFIDENTIAL

- Overall Survival (OS) is defined as the time from study treatment initiation to death (of any cause). 1-year OS rate and median OS will be reported.
- Safety profile of the association Regorafenib + Avelumab: Toxicity will be graded using the common toxicity criteria from the NCI v5.0.
- Pharmacodynamic activity:
    - archived tumor tissue will be collected for assessment of the tumor microenvironment.
    - to perform integrative assessment of biomarkers of efficacy (genetic, metabolomics profiling in blood/tissue at baseline and different study time points) and its prognostic value on efficacy.
- For the cohort B: To evaluate the antitumor activity based on Choi criteria in terms of 6-month PFR.

10. STATISTICAL CONSIDERATIONS

10.1. HYPOTHESES AND NUMBER OF SUBJECTS NEEDED

10.1.1. Phase I trial

- The dose escalation part is designed to detect the MTD of Regorafenib given in association with Avelumab.
- The dose escalation design to identify the maximum tolerated dose will be the traditional 3+3 design. Adaptive designs, such a continual reassessment method (CRM) using likelihood inference do not appear relevant in this context given there are only three dose levels investigated and the toxicity profile of Regorafenib.
- A minimum of 3 patients and a maximum of 6 patients per dose level
- Since there are three dose levels investigated, a maximum of 12 eligible patients assessable for DLT is expected.

10.1.2. Phase II trials: Cohorts A, B, C, D, E, F, G

Independently for each of these phase II trials, a Bayesian approach will be used following an adaptive trial design, allowing for smaller and more informative trials, specifically tied to decision making within a drug development program. This process allows updating knowledge gradually rather than restricting revisions in a trial design with fixed sample sizes.

In each phase II trial:
- A maximum sample size of 50 patients will be included.
- The analysis of the primary endpoint (ORR under treatment for cohorts A as well as C to G; 6-month PFR for cohort B) will be carried out sequentially, with interim analyses planned after 16-week follow-up of the first 10 patients and then every 5 patients. Inclusions will not be suspended between interim analyses, except in case of an important accrual rate in a given given trial (2 patients/center/months during at least 3 months).
- ORR (for cohorts A as well as C to G) or 6-month PFR (for cohort B) will be estimated in the eligible population assessable for efficacy (section 10.2.2).The probability of success (ORR under treatment or 6-month PFR) will be estimated from a beta-binomial model (Zohar et al., 2008). Initial parameters of the model will be pre-specified (the prior distribution represents the knowledge of the non-progression probability prior to observing the data). Successive results observed will then be used to update and refine the distribution, generating the so-called posterior distribution.
- In the absence of a strong idea about the response rates to be observed, a non-informative prior distribution (beta (1,1)) will be considered.
- Maximal response probability thresholds (based on the investigator' opinions) and minimal response probability threshold have been defined as follows:
    - Cohort A/E: p0=5% versus p1=20%
    - Cohorts B/C/D/F: p0=20% versus p1=40%
    - Cohorts G: p0=10% versus p1=25%
- At each update of the distribution (interim analysis), a stopping rule for inefficacy will recommend stopping the trial if there is a high predictive probability (≥80%) that the ORR (for cohorts A as well as C to G) or 6-month PFR (for cohort B) is lower or equal to the minimal response probability threshold p0 defined for the cohort (Strata A and E: p0=5%; Strata B/C/D/F: p0=20%; Stratum G: p0=10%).
- At each update of the distribution (interim analysis), a stopping rule for efficacy will recommend stopping the trial if there is a high predictive probability (≥80%) that the ORR (for cohorts A as well

CONFIDENTIAL

as C to G) or 6-month PFR (for cohort B) is higher or equal to the the maximal response probability threshold p1 (Strata A and E: p1=20%; Strata B/C/D/F: p1=40%; Stratum G: p1=25%).

- The trial will continue until the stopping rule applied at each interim analysis is met, or until the maximum sample size of 50 patients is reached.
- When a trial is not completed to allow efficacy analysis, descriptive data only are presented.

10.1.3. Phase II trial: Cohort H (NSCLC)

- We rely on a single-arm phase 2 trial based on an optimal two-stage Simon's design [91].
- Treatment will be considered promising if 6-month PFR (>= 20%).
- Hypotheses are the following [62] :
    - 20% 6-month PFR (null hypothesis),
    - 40% acceptable 6-month PFR (alternative hypothesis),
    - 5% 1-sided type I error rate,
    - 80% power,
- A total of 43 eligible and assessable subjects will be necessary, with 13 assessable subjects recruited to the first stage.
- Stage 1: Following the inclusion of the first 13 assessable patients, if 3 or less patients are progression-free at 6 months (complete response, partial response or stable disease), the study will be terminated early. Otherwise, if 4 or more patients are progression-free at 6 months, the second group of subjects will be recruited.
- Stage 2: If at the end of recruitment, 13 patients or more are progression-free at 6 months (out of the 43 eligible and evaluable patients), treatment will be considered worthy of further testing in this disease (efficacy rate >= 20%).
- Inclusions will not be suspended after the recruitment of the first 13 patients while data are being analysed for the first stage of inclusion.
- In order to account for not evaluable patients (+/- 10%), 47 patients will be recruited.

10.1.4. Phase II trial: Cohort I (Solid tumors-TLS+)

- We rely on a single-arm phase 2 trial based on an optimal two-stage Simon's design [91].
- Treatment will be considered promising if 6-month PFR (>=15%).
- Hypotheses are the following:
    - 15% 6-month PFR (null hypothesis),
    - 40% acceptable 6-month PFR (alternative hypothesis),
    - 5% 1-sided type I error rate,
    - 90% power,
- A total of 29 eligible and assessable subjects will be necessary, with 13 assessable subjects recruited to the first stage.
- Stage 1: Following the inclusion of the first 13 assessable patients, if 2 or less patients are progression-free at 6 months (complete response, partial response or stable disease), the study will be terminated early. Otherwise, if 3 or more patients are progression-free at 6 months, the second group of subjects will be recruited.
- Stage 2: If at the end of recruitment, 8 patients or more are progression-free at 6 months (out of the 29 eligible and evaluable patients), treatment will be considered worthy of further testing in this disease (efficacy rate >= 15%).
- Inclusions will not be suspended after the recruitment of the first 13 patients while data are being analysed for the first stage of inclusion.
- In order to account for not evaluable patients (+/- 10%), 32 patients will be recruited.

10.1.5. Phase II trial: Cohort A' (Colorectal @ 80 mg), with immune signature (based on low tumor-associated macrophages infiltrate level)

- We rely on a single-arm phase 2 trial based on an optimal two-stage Simon's design [91].
- Treatment will be considered promising if 4-month PFR >= 20%.
- Hypotheses are the following:
    - 20% 4-month PFR (null hypothesis),
    - 40% acceptable 4-month PFR (alternative hypothesis),
    - 5% 1-sided type I error rate,
    - 80% power,
- A total of 43 eligible and assessable subjects will be necessary, with 13 assessable subjects recruited to the first stage.

CONFIDENTIAL

- Stage 1: Following the inclusion of the first 13 assessable patients, if 3 or less patients are progression-free at 4 months (complete response, partial response or stable disease), the study will be terminated early. Otherwise, if 4 or more patients are progression-free at 4 months, the second group of subjects will be recruited.
- Stage 2: If at the end of recruitment, 13 patients or more are progression-free at 4 months (out of the 43 eligible and evaluable patients), treatment will be considered worthy of further testing in this disease (efficacy rate >= 20%).
- Inclusions will not be suspended after the recruitment of the first 13 patients while data are being analysed for the first stage of inclusion.
- In order to account for not evaluable patients (+/- 10%), 47 patients will be recruited.

10.1.6. Phase II trial : Cohorts J (Urothelial cancer) and L (Triple negative breast cancer)

- We rely on a single-arm phase 2 trial based on an exact single stage design (A'Hern).
- Treatment will be considered promising if 6-month DCR >= 15%.
- Hypotheses are the following:
  - 15% 6-month DCR (null hypothesis),
  - 35% 6-month DCR (alternative hypothesis),
  - 5% 1-sided type I error rate,
  - 80% power,
- A total of 28 eligible and assessable subjects will be necessary. If at the end of recruitment, 8 patients or more have disease control at 6 months (out of the 28 eligible and evaluable patients), treatment will be considered worthy of further testing in this disease (efficacy rate >= 15%).
- In order to account for not evaluable patients (+/- 10%), 31 patients will be recruited.

10.1.7. Phase II trials: Cohort K (HPV-associated cancer)

- We rely on a single-arm phase 2 trial based on an exact single stage design (A'Hern).
- Treatment will be considered promising if 6-month DCR >= 10%.
- Hypotheses are the following:
  - 10% 6-month DCR (null hypothesis),
  - 25% 6-month DCR (alternative hypothesis),
  - 5% 1-sided type I error rate,
  - 80% power,
- A total of 40 eligible and assessable subjects will be necessary. If at the end of recruitment, 8 patients or more have disease control at 6 months (out of the 40 eligible and evaluable patients), treatment will be considered worthy of further testing in this disease (efficacy rate >= 10%).
- In order to account for not evaluable patients (+/- 10%), 44 patients will be recruited.

10.1.8. Phase II trials: Cohorts M (TMB-high solid tumors) and O (Non clear-cell renal carcinoma)

- We rely on a single-arm phase 2 trial based on an exact single stage design (A'Hern).
- Treatment will be considered promising if 6-month PFR >= 20% (Cohort M: Marabelle A et al. Lancet Oncol. 2020 ; cohort O: David F McDermott et al. J Clin Oncol 2021).
- Hypotheses are the following:
  - 20% 6-month PFR (null hypothesis),
  - 40% 6-month PFR (alternative hypothesis),
  - 5% 1-sided type I error rate,
  - 80% power,
- A total of 35 eligible and assessable subjects will be necessary. If at the end of recruitment, 12 patients or more are progression-free at 6 months (out of the 35 eligible and evaluable patients), treatment will be considered worthy of further testing in this disease (efficacy rate >= 20%).
- In order to account for not evaluable patients (+/- 10%), 39 patients will be recruited.

10.1.9. Phase II trials: Cohort N (MSI-high solid tumors)

- We rely on a single-arm phase 2 trial based on an exact single stage design (A'Hern).
- Treatment will be considered promising if 6-month PFR >= 15%.
- Hypotheses are the following:
  - 15% 6-month PFR (null hypothesis),
  - 35% 6-month PFR (alternative hypothesis),

CONFIDENTIAL

- 5% 1-sided type I error rate,
- 80% power,

- A total of 28 eligible and assessable subjects will be necessary. If at the end of recruitment, 8 patients or more are progression-free at 6 months (out of the 28 eligible and evaluable patients), treatment will be considered worthy of further testing in this disease (efficacy rate >= 15%).
- In order to account for not evaluable patients (+/- 10%), 31 patients will be recruited.

10.1.10. Phase II trials: Cohort P (Malignant pleural mesothelioma)

- We rely on a single-arm phase 2 trial based on an exact single stage design (A'Hern).
- Treatment will be considered promising if 6-month PFR >= 10%.
- Hypotheses are the following:
  - 10% 6-month PFR (null hypothesis),
  - 25% 6-month PFR (alternative hypothesis),
  - 5% 1-sided type I error rate,
  - 80% power,
- A total of 40 eligible and assessable subjects will be necessary. If at the end of recruitment, 8 patients or more are progression-free at 6 months (out of the 40 eligible and evaluable patients), treatment will be considered worthy of further testing in this disease (efficacy rate >= 10%).
- In order to account for not evaluable patients (+/- 10%), 44 patients will be recruited.

10.2. DEFINITION OF STUDY POPULATIONS

10.2.1. Phase I (Dose escalation part)

The following patients will be included in the population assessable for safety (primary analysis):

- Patients who received > 75% RDI for Regorafenib AND Avelumab over cycle 1,
- Patients who received ≤ 75% RDI for Regorafenib OR ≤ 75% RDI for Avelumab over cycle 1 due to any drug-related AE.

Rules of decision for patient's replacement:

- Patients who goes off treatment for toxicity (DLT or any other AE):
  - If RDI ≤ 75%: Patients will not be replaced. Patients will be considered as having experienced a DLT over the first cycle.
  - If RDI > 75%: Patients will not be replaced. DLT status will follow protocol's definition (section 9.1)
- Patients who goes off treatment for reasons unrelated to toxicity (DLT or any other AE), e.g. progression, lost to follow-up, will be replaced.

10.2.2. Phase II trials

Independently for each phase II trial:

- Eligible population: All patients included without major violation of eligibility criteria.
- Eligible population assessable for efficacy: All patients eligible and for whom the following conditions are statisfied:
  - Received at least one complete cycle over the first two cycles (i.e. both Regorafenib and Avelumab received fully over at least one cycle) or two incomplete treatment cycles (i.e. at least Regorafenib or at least Avelumab partially administered over each of the first two cycles),
  - At least one disease measurement recorded not less than eight weeks after treatment onset.
- The following patients will also be included in the population assessable for efficacy; they will be considered as "inevaluable for response" for the primary endpoint as per RECIST v1.1 criteria (i.e. objective response under treatment) and not be replaced in the primary efficacy analysis:
  - Any eligible patients who received at least one treatment cycle or two incomplete treatment cycles and experience disease progression or die due to disease progression prior to response evaluation (will be considered as "inevaluable for response" due to early progression or death related to progression).
- Safety population: all patients who initiated treatment (Regorafenib or Avelumab).

CONFIDENTIAL

Rules of decision for patient's replacement:

- Any patient not eligible or not assessable for efficacy will be replaced.
- However, any patient who received at least one administration of the study drug will be included in the safety analysis.

10.3. STATISTICAL ANALYSIS

Two statistical analysis plans (SAP) will be produced by the statistician and validated by the steering committee before (i) the first statistical analysis of the phase I trial and (ii) the first statistical analysis of the phase II trial. At the end of the dose escalation part of the phase I trial and before opening the phase II trials, an IDMC (see section 12) will be set up to assess safety data and validate MTD/RP2D based on the statistical report produced by the statistician of the study.

10.3.1. Patient characteristics at baseline

For the phase I trial, as well as independently for each phase II trial:

The patients entered into the study will be described according to the following characteristics:

- Compliance with eligibility criteria,
- Epidemiological characteristics,
- Clinical and laboratory characteristics,
- Treatment characteristics.

10.3.2. Endpoint analyses

10.3.2.1. PHASE I TRIAL (DOSE ESCALATION PART)

- All analyses will be descriptive; no p-values will be calculated.
- Primary endpoint will be analyzed on the population assessable for safety of the phase I trial (escalation part).
- Toxicity observed at each dose level, graded according to the Common Terminology Criteria for Adverse Events v5.0 from the NCI, will be recorded in terms of event type, severity, dates of beginning and end, reversibility and evolution. Data will be gathered in tables summarizing toxicities and side effects for each dose level and cycle.
- DLT will be described in terms of number and incidence rates at each dose level. The number and percentage of patients who will have developed a DLT in each dose level will also be reported.
- Data analyses will be provided by dose groups and for all study patients, combined wherever appropriate.
- Categorical endpoints: best overall response, ORR under treatment and at 6 months, 6-month ORR and 6-month PFR, will be reported in terms of counts by dose level.
- Continuous endpoints, e.g. GMI, will be reported in terms of summary statistics that will include number of patients, median, minimum, and maximum, and additional percentiles if appropriate.
- Survival endpoints (PFS and OS) will be analyzed using the Kaplan-Meier method. The median survival rates will be reported with a 95% confidence interval. Median follow-up will be calculated using the reverse Kaplan-Meier method.
- Missing data will not be imputed.

10.3.2.2. PHASE II TRIALS

- All analyses will be descriptive; no p-values will be calculated.
- Analyses will be conducted independently for each phase II trial.
- Analysis of the primary efficacy endpoint will be analysed based on the eligible population assessable for efficacy (see chapter 10.2 for definition):
  - Each patient will be assigned one of the following categories:
    - Confirmed complete response (CR)
    - Unconfirmed complete response (CRu)
    - Confirmed Partial response (PR)
    - Unconfirmed Partial response (PRu)
    - Stable disease
    - Progression (Clinical PD and radiological PD will be reported separately)

CONFIDENTIAL

        - Inevaluable for response
    - Depending on the cohort, the rate of objective response (ORR: CR, CRu, PR, PRu) under treatment or the progression-free rate (PFR: CR, CRu, PR, PRu, SD) will be reported:
        - All eligible and assessable patients (section 10.2) will be included in the denominator for the calculation of the ORR/PFR.
        - The 95% two-sided confidence limits will be provided for the ORR/PFR (binomial law).
        - First endpoint conclusions will be based on the ORR/PFR for all eligible and assessable patients (section 10.2) after centralized radiological review of the data.
- As regards to the other efficacy endpoints, the analyses will be carried out in the eligible and assessable population:
    - Each patient will be assigned one of the following categories:
        - Confirmed complete response (CR)
        - Unconfirmed complete response (CRu)
        - Confirmed Partial response (PR)
        - Unconfirmed Partial response (PRu)
        - Stable disease
        - Progression
        - Inevaluable for response
    - The rate of best overall response will be calculated as the number of patients alive with the best response (recorded from the start of the treatment) divided by the number of patients eligible and assessable (section 10.2).
    - The 6-month progression-free rate will be calculated as the number of patients remaining alive and progression-free at 6 months from the start of the treatment divided by the number of patients eligible and assessable (section 10.2).
    - The 6-month objective response rate will be calculated as the number of patients alive with complete or partial response at 6 months divided by the number of patients eligible and assessable (section 10.2).
    - The 95% two-sided confidence limits will be provided for the calculated rates (binomial law)
- The safety analysis will be performed on the safety population.
- Quantitative variables will be described using mean and standard deviations if the normality assumption is satisfied, else other descriptive statistics (median, range, quartiles) will be reported.
- Qualitative variables will be described using frequency, percentage and 95% confidence interval (binomial law).
- Survival endpoints will be analysed using the Kaplan-Meier method. The median survival rates will be reported with a 95% confidence interval. Median follow-up will be calculated using the reverse Kaplan-Meier method.

10.3.2.3. Phase II trials – Interim safety analysis

- An interim safety analysis will be performed based on the first ten patients included in (any of) the phase II trials.
- This analysis will be performed once data over the first cycle of treatment are available for the first 10 patients.
- Pooled data will be presented.
- Baseline patients’ characteristics, treatment, and safety data (SAE and AE) will be presented over the first cycle of treatment.

10.3.2.4. Phase II trials – Interim efficacy analysis (Cohorts A, B, C, D, E, F and G)

For each cohort: interim efficacy analyses will be performed following the recruitment of the first ten patients, and every five patients thereafter (section 10.1.2).

10.3.2.5. Phase II trials – Interim efficacy analysis (Cohorts H, I and A′)

For each cohort: following the two-stage optimal Simon’s design, one interim efficacy analysis will be performed at the end of the 1st stage of recruitment (sections 10.1.3 to 10.1.5).

10.3.2.6. Phase II trials – Interim efficacy analysis (Cohorts J to P)

No interim analysis is foreseen.

CONFIDENTIAL

11. ADVERSE EVENTS

11.1. DESCRIPTION OF SAFETY EVALUATION CRITERIA

The safety evaluation will comprise an evaluation of the patient's general condition (ECOG Appendix 1), a physical exam, regular blood tests and the recording of adverse events occurring throughout the study. Toxicity will be evaluated using the NCI-CTCAE scale, version 5 available on website: http://ctep.info.nih.gov. All appropriate treatment areas should have access to a copy of the CTCAE version 5.

In an emergency situation, the patient, his/her friends/family or treating physician will contact the investigator to report an event and/or to discuss the treatments to be implemented.

11.2. DEFINITION

11.2.1. Adverse event

An adverse event is the development of an undesirable medical condition or the deterioration of a pre-existing medical condition following or during exposure to a pharmaceutical product, whether or not considered causally related to the product. An undesirable medical condition can be symptoms (eg, nausea, chest pain), signs (eg, tachycardia, enlarged liver) or the abnormal results of an investigation (eg, laboratory findings, electrocardiogram). In clinical studies, an AE can include an undesirable medical condition occurring at any time, including run-in or washout periods, even if no study treatment has been administered.
The term AE is used to include both serious and non-serious AEs.

11.2.2. Serious adverse event

A serious adverse event is an AE occurring during any study phase (i.e., screening, run-in, treatment, wash-out, follow-up), at any dose of the study drugs that fulfils one or more of the following criteria:

- Results in death
- Is immediately life-threatening
- Requires in-patient hospitalization or prolongation of existing hospitalization
- Results in persistent or significant disability or incapacity
- Is a congenital abnormality or birth defect
- Is an important medical event that may jeopardize the patient or may require medical intervention to prevent one of the outcomes listed above.

The causality of SAEs (their relationship to all study treatment/procedures) will be assessed by the investigator(s) and communicated to Sponsor.

Any suspected transmission via a medicinal product of an infectious agent, pathogenic or non-pathogenic, is assessed as a serious adverse event with the seriousness criterion important medical event. The event may be suspected from clinical symptoms or laboratory findings indicating an infection in a patient exposed to a medicinal product. The terms "suspected transmission" and "transmission" are considered synonymous.
Any abnormal laboratory's result resulting as a grade 4 in the CTCAE version 5 will be considered as serious adverse event even if this event is not clinically relevant.
Whether or not corresponding to the above-mentioned criteria, any other adverse event considered as serious by any IMP, any healthcare professional or any investigator should be handled as a serious adverse event.

11.2.2.1. DEATH

Death as such is the outcome of a SAE or the seriousness criteria and should not be used as the SAE term itself. Instead the cause of death should be recorded as the SAE term. When available, the autopsy report will be provided to the Sponsor.

11.2.2.2. LIFE-THREATENING EVENT

Any event in which the patient was at risk of death at the time of the event; it does not refer to an event which hypothetically might have caused death if it were more severe.

11.2.2.3. HOSPITALIZATION OR PROLONGATION OF HOSPITALIZATION

Any AE requiring hospitalization (or prolongation of hospitalization) that occurs or worsens during the course of a patient's participation in a clinical trial must be reported as a SAE. Prolongation of hospitalization is defined as any extension of an inpatient hospitalization beyond the stay anticipated/required for the initial admission, as determined by the Investigator or treating physician.
Hospitalizations that do not meet criteria for SAE reporting are:

- Reasons described in protocol [e.g., investigational medicinal product (IMP) administration, protocol-required intervention/investigations, etc]. However, events requiring hospitalizations or prolongation

CONFIDENTIAL

of hospitalization as a result of a complication of therapy administration or clinical trial procedures will be reported as SAEs.

- Hospitalization or prolonged hospitalization for technical, practical or social reasons, in absence of an AE. However, these circumstances will be collected in the CRF.
- Pre-planned hospitalizations: Any pre-planned surgery or procedure must be documented in the source documentation and collected in the CRF. Only if the pre-planned surgery needs to be performed earlier due to a worsening of the condition, should this event (worsened condition) be reported as a SAE.

11.2.3. Non serious adverse event

A non-serious adverse event is an adverse event whose characteristics do not meet the criteria of a serious adverse event.

11.2.4. Adverse effect

An adverse effect is any untoward and unintended responses to an experimental drug regardless of the dose.

11.2.5. Expected/Unexpected character

An unexpected adverse event is an event whose nature, frequency, severity/intensity or outcome does not correspond to the information shown within the reference document for the study. The Sponsor will use as the reference safety information for the evaluation of listedness/expectedness the most updated Investigator's Brochure (IB) and/or Summary Product Characteristic (SmPC) for the studied IMP.
In practice, the term "new effect" is sometimes used as a synonymous of "unexpected adverse effect".

11.2.6. Intensity criterion

It is important to distinguish between serious and severe AEs. Severity is a measure of intensity whereas seriousness is defined by the criteria in Section 11.2.2. An AE of severe intensity need not necessarily be considered serious. For example, nausea that persists for several hours may be considered severe nausea, but not a SAE. On the other hand, a stroke that results in only a limited degree of disability may be considered a mild stroke but would be a SAE.
The CTEP Active Version of the NCI Common Terminology Criteria for Adverse Event (CTCAE) will be utilized for AE reporting.
The intensity of adverse events not listed in this classification will be assessed using the following descriptors:
Mild (grade 1): does not affect the patient's usual daily activities,
Moderate (grade 2): disturbs the patient's usual daily activities,
Severe (grade 3): prevents the patient's usual daily activities,
Very severe (grade 4): requires critical care/life-threatening,
Death (grade 5).

11.2.7. New information

A new information is any new safety data that could lead to reevaluate the ratio between the benefits and risks of the research or the investigational product, to modify the use of the investigational product, the conduct of the research or the research documents or to suspend or interrupt or modify the protocol of research or similar research.
For trials of first administration or use of a health product in persons without any conditions: any serious adverse reactions. The study REGOMUNE is not concerned by this situation.

11.2.8. Special considerations

Certain product safety monitoring reports should be forwarded even if there is no associated adverse event. These reports involve circumstances that may increase the patient/consumer's risk of developing adverse events.
These circumstances include:

- medication errors,
- exposure during pregnancy,
- exposure during breastfeeding,
- overdose,
- misuse,
- occupational exposure.

Some of these special circumstances are considered in more details below.

CONFIDENTIAL

Overdose
There is currently no specific treatment in the event of overdose with study drug and possible symptoms of overdose are not established.
Study drug must only be used in accordance with the dosing recommendations in this protocol. Any dose or frequency of dosing that exceeds the dosing regimen specified in this protocol should be reported as an overdose.
Adverse reactions associated with overdose should be treated symptomatically and should be managed appropriately.
If an overdose drug occurs in the course of the study, the sponsor inform appropriate representatives within one day, i.e., immediately but no later than the end of the next business day of when he or she becomes aware of it.
The sponsor works with the investigator to ensure that all relevant information is provided.
For overdoses associated with SAE, standard reporting timelines apply, see Section 11.3. For other overdoses, reporting should be done within 30 days.

Medications errors: a medication error is any preventable event that may cause or lead to inappropriate medication use or patient harm while the medication is in the control of the healthcare professional, patient, or consumer. Such events may be related to professional practice, healthcare products, procedures, and systems, including: prescribing; order communication; product labeling, packaging, and nomenclature; compounding; dispensing; distribution; administration; education; monitoring; and use.
A medication error does not necessarily involve the administration of the product (e.g. the error may have been corrected prior to administration of the product).
Potential medication errors or "near-misses," which are individual reports of information or complaints about product name, labeling, or packaging similarities that do not involve a patient, are also reportable.

Exposure during pregnancy:
Exposure during pregnancy refers to pregnancies where the fetus (from pre-embryo to birth) may have been exposed at a given time during pregnancy to a medicinal product (or a blinded treatment). Even if there is no associated adverse event, exposure during pregnancy must always be reported. It can indeed provide the opportunity to obtain pregnancy outcome important information where appropriate.
Exposure during pregnancy may occur either:

- Through maternal exposure
  - A female becomes, or is found to be, pregnant either:
    - While receiving a medicinal product
    - After discontinuing a medicinal product
    - During or following environmental exposure to a medicinal product (eg, a nurse reports she is pregnant and that she was exposed to chemotherapy drugs via inhalation or after accidentally overturning a bottle)

or

-Through paternal exposure
  - A male has been exposed to a medicinal product (either due to treatment or environmental circumstances) prior to or around the time of conception and/or is exposed during the partner pregnancy.

Exposure during breastfeeding: exposure during breastfeeding occurs where an infant or child may have been exposed through breast milk to a medicinal product during breastfeeding by a female taking the product.

All drug exposure during breastfeeding cases are reported, whether or not there is an associated adverse event.

Occupational exposure: this refers to the exposure to a medicinal product, as a result of one's professional or non-professional occupation.
All occupational exposure to Avelumab (even if not associated with an adverse event) should be reported as an SAE.

11.2.9. Causal relationship

Medical judgment should be used to determine the relationship, considering all relevant factors, including pattern of reaction, temporal relationship, de-challenge or re-challenge, confounding factors such as concomitant medication, concomitant diseases and relevant history. Assessment of causal relationship should be recorded in the case report forms.

- Yes: There is a reasonable causal relationship between the investigational drug administered and the AE.

CONFIDENTIAL

- No: There is no reasonable causal relationship between the investigational drug administered and the AE.

In case of multiples drugs, the causal relationship must be provided by the investigator for all potential trial drugs, i.e. for all other trial drugs.

11.3. SERIOUS ADVERSE EVENT AND NEW INFORMATION NOTIFICATION (RESPONSIBILITY OF THE INVESTIGATOR)

Serious adverse events will be reported by the investigator in the patient's CRF and will be followed up until complete resolution.

The investigator will notify the Vigilance Unit without delay about any serious adverse events or new events occurring:

- From the date of the informed consent is signed,
- During the whole patient treatment period as defined by the research,
- Until 90 days after the last dose of study drugs or the initiation of new anti-cancer therapy, whichever is earlier,
- Beyond this period of time, only those SAEs suspected to be related to the study treatment or the research (diagnostic procedures, examinations carried out during the research …) will be collected without any limitation in terms of deadline. Nonetheless, the Sponsor will evaluate any safety information related to the clinical trial that is spontaneously reported by an Investigator beyond the time frame specified in the protocol.

Type of Event	Reporting procedure	Deadline for reporting to the sponsor
SAE	SAE Notification form + written report form if necessary	To be reported immediately to the sponsor
New information	Written report form	To be reported immediately to the sponsor
Pregnancy	Pregnancy Notification form + Written report form if necessary	As soon as pregnancy is confirmed
Special considerations and events of special interest	SAE Notification form	To be reported immediately to the sponsor

The investigator must complete the "Serious Adverse Event Notification Form" (Appendix 5) immediately, in English, and assess the relationship with the study treatment. The form must then be dated, signed and sent by fax to the following address without delay to:

CELLULE DE VIGILANCE (VIGILANCE UNIT) – R&D UNICANCER
Fax: +33 1 44 23 55 70
Or contact : R&D Unicancer – Mail : pv-R&D@unicancer.fr

For each event, the investigator will record:

- A description of the event that is as clearly as possible, using medical terminology,
- The date AE met criteria for serious AE,
- The seriousness criteria,
- The date of hospitalization and the date of discharge,
- The probable cause of death and the date of death if appropriate,
- The date the event started and ended,
- The patient's relevant medical history,
- The steps taken and whether or not corrective treatment was required, whether or not the investigational treatment was discontinued, etc.
- Concomitant medications / therapies
- The causal link between this event and the study treatment, disease treated or an intercurrent disease or treatment, or any obligation imposed by the research (a treatment-free period, additional examinations requested as part of the research etc.),

CONFIDENTIAL

- Clinical course. If the event was not fatal, it should be monitored until recovery, until the patient has returned to his/her previous condition, or until any sequelae have stabilized,
- Whenever possible, the investigator must also attach the following with the serious adverse event report:
  - A copy of the hospitalization or extended hospitalization report,
  - A copy of the autopsy report, if required,
  - A copy of all the results of any additional tests performed, including relevant negative results, along with the normal laboratory values,
  - Any other document he or she considers useful and relevant.

All these documents must be anonymized. Additional information may be requested (by fax, by telephone or during a visit) by the CRA and/or by the Vigilance Unit using a follow-up request form.

The investigator is responsible for providing appropriate medical follow-up for patients until resolution or stabilization of the adverse event or until the patient’s death. Sometimes this may mean that follow-up will extend beyond the patient’s withdrawal from the trial.

The investigator keeps the documents about the presumed adverse effect so that the information previously sent can be added to if necessary.

The investigator responds to requests for additional information from the Vigilance Unit in order to document the original observation.

11.4. EVENTS OF SPECIAL INTEREST

Potential drug-induced liver injury (DILI) are considered important medical events and should be reported as SAEs.

Patients with transaminase increase and/or alkaline phosphatase (AP)/gamma-glutamyl transpeptidase (GGT) increased with or without total bilirubin (TBIL) increase may be indicative of potential drug-induced liver injury (DILI).

The threshold for potential DILI may depend on the patient’s baseline AST/ALT and TBIL values; patients meeting any of the following criteria will require further follow-up as outlined below:

- For patients with normal ALT and AST and TBIL value at baseline: AST or ALT > 3.0 x ULN and with TBIL > 2.0 x ULN
- For patients with elevated AST or ALT or TBIL value at baseline: AST or ALT > 2 x baseline and > 3.0 x ULN and TBIL > 2 x baseline and > 2.0 x ULN

Medical review needs to ensure that liver test elevations are not caused by cytolysis and/or cholestasis due to other causes, especially liver progression.
The evaluation should include laboratory tests, detailed history, physical assessment and the possibility of liver metastasis or new liver lesions, obstructions/compressions.

All cases confirmed with no other alternative cause for liver function test abnormalities identified should be reported as SAE. All events should be followed up with the outcome clearly documented.

For management of hepatic toxicity, refer to appendix 8.

For more information, refer to FDA’s guidance document:
https://www.fda.gov/downloads/Drugs/Guidance/UCM174090.pdf.

Skin rash grade ≥ 3, associated or not with fever, are considered important medical events and should be reported as SAEs (see section 11.3).

11.5. REPORTING PREGNANCY CASES OCCURRED WITHIN THE CLINICAL TRIAL

If a patient becomes pregnant during the course of the study, study drugs should be discontinued immediately.
The outcome of any conception occurring from the date of the first dose until 3 months after the last dose should be followed up and documented.
Pregnancy itself is not regarded as an adverse event unless there is a suspicion that the investigational product under study may have interfered with the effectiveness of a contraceptive medication. Congenital abnormalities/birth defects and spontaneous miscarriages should be reported and handled as SAEs. Elective abortions without complications should not be handled as AEs. The outcome of all

pregnancies (spontaneous miscarriage, elective termination, ectopic pregnancy, normal birth or congenital abnormality) should be followed up and documented even if the patient was withdrawn from the study.
If any pregnancy occurs in the course of the study, then Investigators or other site personnel must inform Sponsor within one day i.e., immediately but no later than the end of the next business day of when he or she becomes aware of it. Any pregnancy, suspected pregnancy, or positive pregnancy test must be reported to the Vigilance Unit immediately by facsimile using the Pregnancy Report form (appendix 6).

Paternal exposure: Male patients should refrain from fathering a child for 4 months (120 days) following the last dose or donating sperm during the study and for 4 months following the last dose.
Pregnancy of the patient's partners is not considered to be an adverse event. However, the outcome of all pregnancies (spontaneous miscarriage, elective termination, ectopic pregnancy, normal birth or congenital abnormality) should if possible be followed up and documented.
The outcome of any conception occurring from the date of the first dose until 7 months after the last dose should be followed up and documented.
In the case of pregnancy of the female partner of a trial patient, the Investigator will obtain her consent to provide the information in these situations.

The Investigator will follow the pregnancy until its outcome, and must notify the Vigilance Unit the outcome of the pregnancy within 24 hours of first knowledge as a follow-up to the initial report.

For any event during the pregnancy which meets a seriousness criterion (including fetal or neonatal death or congenital anomaly) the Investigator will also follow the procedures for reporting SAEs (complete and send the SAE form to the Vigilance Unit by facsimile within 24 hours of the Investigator's knowledge of the event).
All neonatal deaths that occur within 30 days of birth should be reported, without regard to causality, as SAEs. In addition, any infant death at any time thereafter that the Investigator suspects is related to the exposure to the study drug/IMP should also be reported to the Vigilance Unit by facsimile within 24 hours of the Investigators' knowledge of the event.
Whenever possible, the investigator must also attach the following with the serious adverse event report:
- A copy of the hospitalization or extended hospitalization report,
- A copy of the autopsy report, if required,
- A copy of all the results of any additional tests performed, including relevant negative results, along with the normal laboratory values,
- Any other document he or she considers useful and relevant.

All these documents must be anonymized.
Additional information may be requested (by fax, by telephone or during a visit) by the Vigilance Unit.

11.6. NON SERIOUS ADVERSE EVENT

TYPE OF EVENT	REPORTING PROCEDURES	DEADLINE FOR REPORTING TO THE SPONSOR
Non-serious AE	Case report/record form	Does not need to be reported immediately

Non-serious adverse events will be reported by the investigator in the patient's CRF and will be followed up until complete resolution.

The following variables will be collected for each AE:

- AE (verbatim)
- The date when the AE started and stopped
- Minimum and maximum intensity
- Whether the AE is serious or not
- Outcome
- Investigator causality rating against the Investigational Product (yes or no) radiotherapy (yes/no)
- Action taken with regard to investigational product/comparator/combination agent
- Treatments given to treat AE

If an adverse event becomes serious, it should be reported and followed-up as mentioned in the previous reporting procedures.
If the investigator would like to decrease trial treatment dose or temporarily stop study management without respecting protocol procedures, he/she should have previously discussed with the coordinator.
However, symptomatic treatment can be prescribed to manage the adverse event.

CONFIDENTIAL

Any definitive interruption of the procedure has to be immediately notified to the sponsor. The patient remains in the study and is followed-up according to the procedures described in the protocol.

11.7. RESPONSIBILITY OF VIGILANCE UNIT

The Vigilance Unit will analyze each SAE to define:
- The relationship with the study treatment,
- The listedness/expectedness according to the most updated reference safety information of the studied IMP Investigator's Brochure (IB) and/or Summary Product Characteristic (SmPC).

11.8. NOTIFICATION AND REGISTRATION OF UNEXPECTED SERIOUS ADVERSE EVENTS AND NEW INFORMATION (RESPONSIBILITY OF THE SPONSOR)

The sponsor notifies unexpected serious adverse events and new information to the Regulatory Authorities (in person, or through an organization which has received allowances for this task) according to the usual notification procedures.

12. QUALITY ASSURANCE AND TRIAL MONITORING

12.1. MONITORING OF THE TRIAL

12.1.1. Steering Committee

The study will be supervised and monitored by a Steering Committee comprising members participating in the study:

- Dr S. Cousin, Co-ordinating Investigator and Chairman of the Commitee,
- Pr A. Italiano, Investigator and medical oncologist,
- A representative of the sponsor (Pr S. Mathoulin-Pélissier or a substitute).
- The biostatistician of the trial (Ms. C. Bellera, or a substitute).
- The pharmacist of the trial (Ms. L. Poignie, or a substitute).
- The co-ordinating Clinical research manager (D. Juzanx, or a substitute)

This committee must ensure the following:

- Implementation and regular follow-up of the study
- Patient protection,
- That the trial is conducted ethically, in accordance with the protocol,
- That the trial benefit/risk ratio is evaluated and the scientific results are checked during or at the end of the trial.

For the dose escalation part, the steering committee will be consulted before proceeding to the next dose level. In addition, the steering committee will resolve any specific issue regarding the DLT status. Note however, that medical oncologists of the steering committee cannot comment on the DLT status of patients they have been in charge of. In such case, the opinion of another medical oncologist who has not treated the patient will be sought.

It decides on any relevant amendment to the protocol that is required in order to continue the trial (protocol amendments prior to submission to the EC and the relevant Health Authorities, decisions on whether to open or close research sites, discussion of results and the strategy for the publication of these results). It must inform the sponsor of any decisions taken. Decisions concerning a major amendment or a change to the budget must be approved by the sponsor.

12.1.2. Independent Data Monitoring Committee

- An independent Data Monitoring Committee (IDMC) will be created at the request of the relevant Authority, the sponsor or the Steering Committee. The IDMC plays an advisory role for the Sponsor, who has the final decision regarding the implementation of recommendations put forward by the IDMC.
- The sponsor may decide to submit the conclusions of the steering committee for the MTD and RP2D definitions based on the dose escalation trial to the IDMC before opening the phase II trials.
- Implementation of the IDMC committee will be performed according to the internal procedures at Institut Bergonié.

CONFIDENTIAL

Composition of the IDMC

- This Committee must comprise at least one qualified oncologist, one pharmacologist and one statistican, all of whom will have experience in the monitoring and analysis of clinical trials. One of these members will be appointed as the Trial Rapporteur.Each of these members must be unconnected with the trial and cannot, therefore, be one of the trial investigators.
- These members are appointed by the Sponsor in consultation with the trial co-ordinator and the Steering Committee.

Responsibilities of the IDMC

The IDMC is responsible for the following:

- Analyzing preliminary efficacy and safety data, specifically the statistical report presenting results for the dose escalation part will be presented to the IDMC experts;
- Making recommendations on the continuation, early discontinuation (in the case of toxicity or lack of efficacy) or publication of the trial results,
- Drafting the minutes after each meeting and monitoring their confidentiality.

Any recommendation from the IDMC that can be made public will be announced by the Sponsor and not by the Steering Committee. The Sponsor is responsible for sending IDMC recommendations to the regulatory authorities [ANSM (French Agency for the Safety of Health Care Products) and EMEA (European Medicines Evaluation Agency)].

12.2. QUALITY ASSURANCE

12.2.1. Data collection

The data will be collected on an electronic case report form and directly input via the Internet. Only the investigators and the Investigator's Clinical Research Assistants (CRAs) appointed by the later and duly authorized by the sponsor will be authorized to enter the data.

Data will be handled by an online trial management software on the Internet (Macro v4, Infermed Company); it will be transferred and monitored remotely in real time.

The study CRA and/or any other person appointed by the sponsor will be available to assist the investigators in carrying out the study and to ensure that the trial is carried out in accordance with the protocol.

The study CRA will contact the investigators regarding the study implementation visit.

All of the necessary data will be collected on an electronic case report form provided by the sponsor. The generic names of the concomitant medication will be given in French.

Corrections made to the original data must be justified. These corrections will be automatically dated and signed by the authorized member of staff via the personalized password allocated at the start of the study.

The case report form will be validated by the investigator or the CRA at the authorized center whenever data is entered.

Laboratory data exceeding normal limit values will be commented upon if they are considered clinically significant. Data other than that requested within the scope of the protocol can be collected as additional data; their interest will be specified.

12.2.2. Monitoring

In order to guarantee the authenticity and credibility of the data in accordance with the principles of GCP (Good Clinical Practice) dated 24 November 2006, the sponsor shall implement a quality assurance system comprising:

- the management and monitoring of the trial in accordance with the procedures stipulated by the Institut Bergonié,
- the quality control of the research site data by the CRA whose role is to:
  - check compliance with the protocol, GCP and current legislation and regulations,
  - check the consent and eligibility of each patient taking part in the trial,
  - check the consistency and coherence of case report form date against the source documents.

CONFIDENTIAL

- check that each serious adverse event is reported,
- monitor the traceability of the study medication (dispensation, storage and drug accountability),
- check, where applicable, that the persons likely to take part in the trial are not already participating in another trial that could prevent them from being included in the clinical trial proposed. The CRA shall also ensure that the patients have not participated in a trial for which an exclusion period currently applies.

- The possible audit of study centers
- The centralized review of certain protocol criteria.

The check procedures will include:

- Study progression,
- Protocol compliance,
- The updating of information on the Internet site.

The checking of data by comparing the information on the electronic case report form and the original clinical or laboratory data is one of the monitoring procedures.

The following will be checked, in particular, for each patient (100% level): patient identification, informed consent (procedure and signature), selection criteria, therapeutic procedure, adverse events, principal response variables. The personal data relating to each patient shall remain confidential. On the electronic case report form or any other form dispatched, the patients will be identified solely by their initials (1/name – 1/surname) and an inclusion number. However, the investigators must keep a list identifying the patients in their folders.

The CRAs responsible for the quality control of this clinical trial are duly appointed by the sponsor for this particular purpose and must have access, with the consent of those involved, to individual trial participant data required strictly in accordance with this control procedure. The CRAs are subject to professional secrecy under the conditions defined by Articles 226-13 and 226-14 of the French penal code. The traceability of monitoring visits is guaranteed by a written monitoring report.

The investigators shall undertake to give CRAs direct access to the medical records of each patient in order to allow the CRAs to ensure optimal quality control of the trial. The same applies to health authority representatives.

12.2.3. Handling of missing data

The monitoring of data for adverse events will be carried out regularly in order to effectively limit the amount of missing data likely to prevent or hamper trial implementation and analysis.

12.2.4. Audits

The sponsor, the local authorities or the authorities to which information about this study has been submitted can decide to have an audit. All the documents relating to this study must be available for such an inspection after prior notification.

12.2.5. Data management

The data are entered using an electronic case report form (e-CRF) created with Macro 4.2 (Infermed limited 2010). Data entry is performed by the CRA-I using login and password provided by the database administrator. It is carried out at the research unit of Bergonie Institute.
Each step of the data management is described in the data management plan (DMP) drafted by the data manager. This document is validated by the coordinating investigator, the statistician, the CRA-C and the database administrator and is performed according to the internal procedures of the research unit.
The process of data lock/unlock is performed according to our procedure and after validating a check list.
All data will be backed-up daily and kept for 30 days.

13. REGULATORY ASPECTS AND ETHICAL CONSIDERATIONS

CONFIDENTIAL

The study will be carried out in accordance with:

- Law no. 2012-300 dated 5 March 2012 relating to researchs involving the human person.
- The ethical principles of the current version of the “Declaration of Helsinki” (available on its full version on the site http://www.wma.net).
- Good Clinical Practice (GCP): I.C.H. version 4 of 9 November 2016 and decision dated 24 November 2006 (Official Bulletin of 30 November 2006, text 64).
- European Directive (2001/20/EC) on clinical trial procedures.
- Huriet’s law (No. 88-1138) dated 20 December 1988, concerning the protection of persons taking part in Biomedical Research with the provisions of the Public Health law (No. 2004-806) of 9 August 2004 and implementating decree No. 2006-477 of 26 April 2006 relating to biomedical research.
- The French law on Data Protection and Civil Liberties, No. 78-17 of 6 January 1978 amended by law No. 2004-801, dated 6 August 2004, concerning the protection of persons with regards to the processing of personal data.
- The application of Circular DHOS/INCA/MOPRC/2006/475 of 7 November 2006: the Sponsor shall undertake to register the Trial and thus make it accessible to the general public, in the INCa (French Cancer Institute) register via the Internet site: www.e-cancer.fr. Each trial published in the INCa register will be sent to the NCI for registering on the following site: www.clinicaltrials.gov. The trial will be registered before the first patient is entered into the study. The Sponsor is responsible for updating the study data in order to guarantee the reliability of the information available on-line.
- Law no. 2004-800 dated 6 August 2004, concerning bioethics, amended by law No. 2012-387, dated 22 March 2012.

13.1. CLINICAL TRIAL AUTHORIZATION

This trial is registered under Eudract N° 2016-005175-27.

The protocol has been approved by the Ethics Committee,Sud Est II. Approval was given on 06/03/2018.

The Relevant Authority, the Agence Nationale de Sécurité du Médicament et des Produits de Santé (ANSM - French Agency for the Safety of Health Care Products) authorized the clinical trial on 14/03/2018.

Any amendments to the protocol concerning study objectives, patient population and principal methods will require an amendment, which must be approved by the EC and l'ANSM. The sponsor will inform the EC and ANSM of expected and/or unexpected serious adverse events in accordance with current regulations and within 30 days after of completion of the trial.

The sponsor will send the summary of the final report to the relevant Authority within one year of completion of the trial.

The sponsor has made a commitment to compliance the Reference methodology for the processing of personal data carried out in biomedical research: Référence methodology MR-001. This commitment of compliance is registred under No 118019 of the 07/11/2006.

13.2. INSURANCE POLICY

The Institut Bergonié has taken out an insurance policy (policy No 0100871914011 170004) with société HDI-Gerling, Tour opus 12, 77, Esplanade de la Défense, 92914 PARIS LA DEFENSE through an insurance broker, namely Biomédic Insure (Parc d'Innovation Bretagne Sud, CP 142, 56038 Vannes Cedex, Tel. 02 97 69 19 19) in case compensation is payable to investigators or patients taking part in the study.

13.3. INFORMING AND OBTAINING CONSENT FROM PATIENTS

Prior to carrying out medical research on human patients,a free and written informed consent form must be signed by each individual participating in the trial after she/he has been informed by the investigator and after sufficient time for reflection has been allowed.

CONFIDENTIAL

The investigator in charge of the patient will provide the latter with relevant information relating to the study objectives, potential benefits and possible adverse events. The study methods will be outlined. The patient can refuse treatment before or at any time during the study, without experiencing any adverse repercussions in terms of his/her subsequent care.

The patient's written consent will be obtained prior to entry into the study by using the Patient Information Leaflet and Informed Consent Form (appendix 7 to 9). These forms must be combined in the same document in order to ensure that all of the information is given to the trial participant.

The consent form must be personally dated and signed by the trial participant and the investigator. The original will be given to the patient and the second, archived in the investigator's folder. Upon request, a copy will be sent to the sponsor in a sealed envelope.

13.4. SPONSOR'S RESPONSIBILITIES

The sponsor of the clinical trial, the Institut Bergonié, will take the initiative for this clinical trial. The Institute will manage the trial and ensure that finance is provided.

The sponsor's main responsibilities are to:

- Take out civil liability insurance,
- Obtain the Eudract No. and register the trial in the European database (European Drug Regulatory Authorities Clinical Trials),
- Obtain clinical trial authorization for the initial project and any amendments from the EC and ANSM; approval by the EC and decision taken by ANSM.
- Notify the relevant authority any suspected unexpected serious adverse reaction (SUSAR),
- Give trial-related information to the site directors, pharmacists and investigators,
- Notify the relevant authority of the trial start and end dates,
- Draft the final trial report and sent the summary to ANSM,
- Send the trial results to the relevant authority, EC and investigators,
- Archive essential trial documents in the sponsor's folder for a minimum period of 15 years after the trial has ended.

13.5. INVESTIGATORS' RESPONSIBILITIES

The principal investigator of each establishment concerned undertakes to conduct the clinical trial in accordance with the protocol that was approved by the ethics committee and the relevant authority (ANSM).

The investigator must not make any changes to the protocol without the written consent of the sponsor or without the ethics committee and the relevant authority having authorized the proposed changes.

It is the responsibility of the principal investigator is:

- to provide the sponsor with his/her curriculum vitae as well as those of his/her co-investigators,
- to identify the members of his/her team who are participating in the trial and to define their responsibilities,
- to start patient recruitment after authorization has been obtained from the sponsor,
- to ensure that he/she is available for investigators's meeting anf for "monitoring".

It is the responsibility of each investigator:

- to comply with the confidential nature of the trial,
- to obtain informed consent, signed and dated personally by each trial participant, before any screening procedures specific to the trial are carried out,
- to regularly complete the case report forms (CRFs or e-CRFs) for each of the patients enrolled in the trial and to allow the Clinical Research Assistant (CRA) duly authorised by the Sponsor a direct access to source documents so that the latter can validate the data on the CRF or e-CRF,

CONFIDENTIAL

- to promptly notify the sponsor of any serious adverse event and/or new information occurring during the trial,
- to date, correct and validate corrections on the case report forms (CRFs or e-CRFs) and the Data Query Forms (DQFs),
- to accept regular visits CRA and eventually visits of auditors duly authorised by the Sponsor or inspectors of regulatory authorities,
- to inform trial participants of the overall results of the research on first demand.

13.6. AUTHORITY TO EXECUTE THE TRIAL

The investigator shall certify that he/she is authorized to enter into this agreement and that the terms and conditions of the protocol and agreement do not conflict with other agreements that the investigator may have entered into with any other party, or any other arrangement agreed by the Institution where the investigator is employed.

13.7. REGULATIONS GOVERNING THE COLLECTION OF HUMAN BIOLOGICAL SAMPLES

During the medical procedures to be carried out, samples will be collected for medical purposes. A fraction of these samples will be kept and used for scientific research purposes.

The patient will be informed of this research and provided that he/she approves by signing an informed consent, these samples intended for research will be:

- Initially prepared and stored using a specific technique to preserve them under excellent conditions.
- and secondly, used within the scope of this research.

The preparation, storage and use of these samples will not in any way affect current or future medical care administered to the patient for the purpose of diagnosis or treatment.

The results of this research may, in future, appear in scientific publications. All of the data shall remain anonymous.

Obtaining and using additional samples
This biomarker study is made up of exploratory research that is described in the section “Ancillary Study”.

On completion of the trial, provided that the patient agrees and provided that not all of the samples have been used, the said samples can be used for subsequent scientific research purposes without the approval of the Ethics Committee (EC) and the signing of a new consent form by the patients included.

13.8. FEDERATION DES COMITES DE PATIENTS POUR LA RECHERCHE CLINIQUE EN CANCEROLOGIE (FCPRCC) (FEDERATION OF PATIENT COMMITTEES FOR CLINICAL RESEARCH IN ONCOLOGY)

The Fédération des Comités de Patients pour la Recherche Clinique en Cancérologie (FCPRCC) (Federation of Patient Committees for Clinical Research in Oncology) was created on the initiative of the Fédération Nationale des Centres de Lutte Contre le Cancer (FNCLCC) (Federation of Anti-Cancer Centers) and the Ligue Nationale Contre le Cancer (National Anti-Cancer League) in order to review clinical trial protocols in oncology. This Federation of Patient Committees is co-ordinated by the Office for Clinical and Therapeutic Trials and groups together the League patient committees as well as other health care establishments. The Sponsor undertakes to transmit the protocol to the Federation for review. The Federation undertakes to propose improvements focusing primarily on the quality of the information leaflet, the availability of a treatment and monitoring plan and the suggestion of measures aimed at improving patient comfort.

13.9. DATA PROCESSING

In accordance with the French Law on Data Protection and Civil Liberties of 06 August 2004 and its implementing decrees, the Sponsor shall follow the methodology of reference MR001 of the Commission

CONFIDENTIAL

Nationale de l’Informatique et des Libertés (French National Commission for Data Protection and Liberties).

Furthermore, if the biomedical research data is computer processed or managed by computerized systems, each Center:

- shall check and document the fact that the computerized systems used in the research comply with requirements drawn up in relation to data integrity, accuracy and reliability, as well as compliance with expected performances (i.e. validation);
- shall implement and ensure the monitoring of standard operating procedures relating to the use of these systems;
- shall ensure that the design of these systems allows for data to be amended such that the amendments are documented and that any item of data input cannot be deleted (i.e. maintaining data and amendment audit trail) ;
- shall implement and ensure the monitoring of a secure system that prevents any unauthorized data access;
- shall update the list of persons authorized to amend the data;
- shall keep appropriate back-up copies of the data;
- shall maintain blind status, where applicable (e.g. during data entry and processing);
- shall ensure that personal data used within the scope of the trial is processed in accordance with the conditions defined by law No. 78-17 dated 6 January 1978 relating to data processing, files and liberties modified by law No. 2004-801 of 6 August 2004 and its implementing regulations.

If the data is converted during processing, it must always be possible to compare the original data and observations with the data after conversion.

The system used to identify subjects taking part in the trial must not present with any ambiguity and must allow all of the data collected for each of these subjects to be identified whilst maintaining the confidentiality of the personal data, in accordance with law No. 78-17, duly amended.

The archiving data is performed according to the applicable regulations and under the responsibility of investigator. All data and the patient indentification codes will be kept for at least 15 years after the completion or discontinuation of the trial.

14. CONFIDENTIALITY AND OWNERSHIP OF DATA

All of the information communicated or obtained and the data and results generated by the trial legally belong to as their obtaining the Institut Bergonié, which can use this data at its own discretion.
According to article R 5121-13 of the French Public Health Code, investigators and people who will have to collaborate in the trial shall be bound by professional secrecy with regard to the particular nature of the products studied, trial, trial participants, and results. In particularly, all documentation relating to the trial sent to the investigator should be considered confidential information.
Without the consent of the sponsor, the investigator cannot,give information about trials at anyone, except the Minister in charge of Public Health, public health medical inspectors, public health pharmacists inspectors, the General Director and inspectors of ANSM.
The trial cannot be the subject of any written or verbal comments without the ponsor’s consent.

CONFIDENTIAL

15. PUBLICATION AND VALORISATION

15.1. SCIENTIFIC COMMUNICATION

All of the information arising from this study shall be considered confidential (cf. section 12).
All forms of publication must be submitted to the Steering Committee for review and approval prior to publication (allowing at least 15 working days for abstracts and oral presentations, and 45 working days for written publications). The Steering Committee shall check the accuracy of the information submitted (in order to avoid any inconsistency with that submitted to the Health Authorities), and ensure that confidential information is not inadvertently disclosed. It will also provide additional information as required. In any case, the sponsor will control the first publication.
Furthermore, all memos, manuscripts or presentations must comprise a heading referring without fail to the Institut Bergonié, all of the institutions, investigations, co-operating groups and learned societies that

have contributed to the implementation of the trial, and listing any organizations that have provided financial support.
For the principal publication, either in French or English, the authors are:

- the study coordinator
- the investigators will be listed on a pro rata basis according to the number of patients recruited
- a representative of the trial statistics unit (in the first 3 positions ot two last positions according to degree of involvement in the preparation of publications)

15.2. INFORMATION TO PATIENTS

According to Article L.1122-1 of the Fench Code of Public Health Investigator undertakes to inform trial participants of the overall results of the research on first demand.

CONFIDENTIAL

16. REFERENCES

1. Van Cutsem E, Cervantes A, Nordlinger B, Arnold D, ESMO Guidelines Working Group. Metastatic colorectal cancer: ESMO Clinical Practice Guidelines for diagnosis, treatment and follow-up. Ann Oncol. sept 2014;25 Suppl 3:iii1-9.
2. Douillard J-Y, Siena S, Cassidy J, Tabernero J, Burkes R, Barugel M, et al. Randomized, phase III trial of panitumumab with infusional fluorouracil, leucovorin, and oxaliplatin (FOLFOX4) versus FOLFOX4 alone as first-line treatment in patients with previously untreated metastatic colorectal cancer: the PRIME study. J Clin Oncol. 1 nov 2010;28(31):4697‑705.
3. Van Cutsem E, Köhne C-H, Láng I, Folprecht G, Nowacki MP, Cascinu S, et al. Cetuximab plus irinotecan, fluorouracil, and leucovorin as first-line treatment for metastatic colorectal cancer: updated analysis of overall survival according to tumor KRAS and BRAF mutation status. J Clin Oncol. 20 mai 2011;29(15):2011‑9.
4. Tournigand C, Cervantes A, Figer A, Lledo G, Flesch M, Buyse M, et al. OPTIMOX1: a randomized study of FOLFOX4 or FOLFOX7 with oxaliplatin in a stop-and-Go fashion in advanced colorectal cancer--a GERCOR study. J Clin Oncol. 20 janv 2006;24(3):394‑400.
5. Stintzig S, Jung A, Rossius L et al. Analysis of KRAS/NRAS and BRAF mutations in FIRE-3: a randomized phase III study of FOLFIRI plus cetuximab or bevacizumab as first-line treatment for wild-type (WT) KRAS (exon 2) metastatic colorectal cancer (mCRC) patients. Eur J Cancer 2013; 49 (Suppl 3; LBA 17).
6. Grothey A, Van Cutsem E, Sobrero A, Siena S, Falcone A, Ychou M, et al. Regorafenib monotherapy for previously treated metastatic colorectal cancer (CORRECT): an international, multicentre, randomised, placebo-controlled, phase 3 trial. Lancet. 26 janv 2013;381(9863):303‑12.
7. Demetri GD, von Mehren M, Blanke CD, Van den Abbeele AD, Eisenberg B, Roberts PJ, et al. Efficacy and safety of imatinib mesylate in advanced gastrointestinal stromal tumors. N Engl J Med. 15 août 2002;347(7):472‑80.
8. Demetri GD, van Oosterom AT, Garrett CR, Blackstein ME, Shah MH, Verweij J, et al. Efficacy and safety of sunitinib in patients with advanced gastrointestinal stromal tumour after failure of imatinib: a randomised controlled trial. Lancet. 14 oct 2006;368(9544):1329‑38.
9. Demetri GD, Reichardt P, Kang Y-K, Blay J-Y, Rutkowski P, Gelderblom H, et al. Efficacy and safety of Regorafenib for advanced gastrointestinal stromal tumours after failure of imatinib and sunitinib (GRID): an international, multicentre, randomised, placebo-controlled, phase 3 trial. Lancet. 26 janv 2013;381(9863):295‑302.
10. Stahl M, Mariette C, Haustermans K, Cervantes A, Arnold D, ESMO Guidelines Working Group. Oesophageal cancer: ESMO Clinical Practice Guidelines for diagnosis, treatment and follow-up. Ann Oncol. oct 2013;24 Suppl 6:vi51-56.
11. Waddell T, Verheij M, Allum W, Cunningham D, Cervantes A, Arnold D, et al. Gastric cancer: ESMO-ESSO-ESTRO clinical practice guidelines for diagnosis, treatment and follow-up. Eur J Surg Oncol. mai 2014;40(5):584‑91.
12. Wagner AD, Grothe W, Haerting J, Kleber G, Grothey A, Fleig WE. Chemotherapy in advanced gastric cancer: a systematic review and meta-analysis based on aggregate data. J Clin Oncol. 20 juin 2006;24(18):2903‑9.
13. Cunningham D, Okines AFC, Ashley S. Capecitabine and oxaliplatin for advanced esophagogastric cancer. N Engl J Med. 4 mars 2010;362(9):858‑9.
14. Roy AC, Park SR, Cunningham D, Kang YK, Chao Y, Chen LT, et al. A randomized phase II study of PEP02 (MM-398), irinotecan or docetaxel as a second-line therapy in patients with locally advanced or metastatic gastric or gastro-oesophageal junction adenocarcinoma. Ann Oncol. juin 2013;24(6):1567‑73.
15. Bang Y-J, Van Cutsem E, Feyereislova A, Chung HC, Shen L, Sawaki A, et al. Trastuzumab in combination with chemotherapy versus chemotherapy alone for treatment of HER2-positive advanced gastric or gastro-oesophageal junction cancer (ToGA): a phase 3, open-label, randomised controlled trial. Lancet. 28 août 2010;376(9742):687‑97.
16. Wilke H, MUWilke H, Muro K, Van Cutsem E, Oh S-C, Bodoky G, Shimada Y, et al.

CONFIDENTIAL

Ramucirumab plus paclitaxel versus placebo plus paclitaxel in patients with previously treated advanced gastric or gastro-oesophageal junction adenocarcinoma (RAINBOW): a double-blind, randomised phase 3 trial. Lancet Oncol. oct 2014;15(11):1224‑35.

17. Eckel F, Brunner T, Jelic S. Biliary cancer: ESMO Clinical Practice Guidelines for diagnosis, treatment and follow-up. Annals of Oncology 22 (Supplement 6): vi40–vi44, 2011
18. Verslype C, Rosmorduc O, Rougier P, ESMO Guidelines Working Group. Hepatocellular carcinoma: ESMO-ESDO Clinical Practice Guidelines for diagnosis, treatment and follow-up. Ann Oncol. oct 2012;23 Suppl 7:vii41-48.
19. Llovet JM, Bustamante J, Castells A, Vilana R, Ayuso M del C, Sala M, et al. Natural history of untreated nonsurgical hepatocellular carcinoma: rationale for the design and evaluation of therapeutic trials. Hepatology. janv 1999;29(1):62‑7.
20. Valle JW, Wasan HS, Palmer DD et al. Gemcitabine with or without cisplatin in patients (pts) with advanced or metastatic biliary tract cancer (ABC): results of a multicenter, randomized phase III trial (the UK ABC-02 trial). Proc Am Soc Clin Oncol 2009: 27: abstract 4503.
21. Llovet JM, Ricci S, Mazzaferro V, Hilgard P, Gane E, Blanc J-F, et al. Sorafenib in advanced hepatocellular carcinoma. N Engl J Med. 24 juill 2008;359(4):378‑90.
22. Schmieder R, Hoffmann J, Becker M, Bhargava A, Müller T, Kahmann N, et al. Regorafenib (BAY 73-4506): antitumor and antimetastatic activities in preclinical models of colorectal cancer. Int J Cancer. 15 sept 2014;135(6):1487‑96.
23. Mir O, Brodowicz T, Italiano A, Wallet J, Blay JY, Bertucci F, et al. Safety and efficacy of Regorafenib in patients with advanced soft tissue sarcoma (REGOSARC): a randomized, double-blind, placebo-controlled, phase 2 trial. Lancet Oncol. 2016 Dec;17 (12):1732-1742
24. Grenga I, Donahue RN, Lepone LM, Richards J, Schlom J. A fully human IgG1 anti-PD-L1 MAb in an in vitro assay enhances antigen-specific T-cell responses. Clin Transl Immunology. mai 2016;5(5):e83.
25. Boyerinas B, Jochems C, Fantini M, Heery CR, Gulley JL, Tsang KY, et al. Antibody-Dependent Cellular Cytotoxicity Activity of a Novel Anti-PD-L1 Antibody Avelumab (MSB0010718C) on Human Tumor Cells. Cancer Immunol Res. oct 2015;3(10):1148‑57.
26. Vandeveer AJ, Fallon JK, Tighe R, Sabzevari H, Schlom J, Greiner JW. Systemic Immunotherapy of Non-Muscle Invasive Mouse Bladder Cancer with Avelumab, an Anti-PD-L1 Immune Checkpoint Inhibitor. Cancer Immunol Res. mai 2016;4(5):452‑62.
27. Kaufman HL, Russell J, Hamid O, Bhatia S, Terheyden P, D'Angelo SP, et al. Avelumab in patients with chemotherapy-refractory metastatic Merkel cell carcinoma: a multicentre, single-group, open-label, phase 2 trial. Lancet Oncol. 1 sept 2016;
28. Polydorides AD, Mukherjee B, Gruber SB, McKenna BJ, Appelman HD, Greenson JK. Adenoma-infiltrating lymphocytes (AILs) are a potential marker of hereditary nonpolyposis colorectal cancer. Am J Surg Pathol. 2008 Nov;32(11):1661-6
29. Dong H, Strome SE, Salomao DR, Tamura H, Hirano F, Flies DB, et al. Tumor-associated B7-H1 promotes T-cell apoptosis: a potential mechanism of immune evasion. Nat Med. août 2002;8(8):793‑800.
30. Lipson EJ, Sharfman WH, Drake CG, Wollner I, Taube JM, Anders RA, et al. Durable cancer regression off-treatment and effective reinduction therapy with an anti-PD-1 antibody. Clin Cancer Res. 15 janv 2013;19(2):462‑8.
31. Le DT, Uram JN, Wang H, Bartlett BR, Kemberling H, Eyring AD, et al. PD-1 Blockade in Tumors with Mismatch-Repair Deficiency. N Engl J Med. 25 juin 2015;372(26):2509‑20.
32. Bertucci F, Finetti P, Mamessier E, Pantaleo MA, Astolfi A, Ostrowski J, et al. PDL1 expression is an independent prognostic factor in localized GIST. Oncoimmunology. mai 2015;4(5):e1002729.
33. Balachandran VP, Cavnar MJ, Zeng S, Bamboat ZM, Ocuin LM, Obaid H, et al. Imatinib potentiates antitumor T cell responses in gastrointestinal stromal tumor through the inhibition of Ido. Nat Med. sept 2011;17(9):1094‑100.
34. Seifert A et al. PD-1/PD-L1 Blockade Enhances the Efficacy of Imatinib in Gastrointestinal Stromal Tumor (GIST). JACS 2014; 219, 3, Supplement: S129

CONFIDENTIAL

35. Kim JW, Nam KH, Ahn S-H, Park DJ, Kim H-H, Kim SH, et al. Prognostic implications of immunosuppressive protein expression in tumors as well as immune cell infiltration within the tumor microenvironment in gastric cancer. Gastric Cancer. janv 2016;19(1):42‑52.

36. Ohigashi Y, Sho M, Yamada Y, Tsurui Y, Hamada K, Ikeda N, et al. Clinical significance of programmed death-1 ligand-1 and programmed death-1 ligand-2 expression in human esophageal cancer. Clin Cancer Res. 15 avr 2005;11(8):2947‑53.

37. Muro K, Chung HC, Shankaran V, Geva R, Catenacci D, Gupta S, et al. Pembrolizumab for patients with PD-L1-positive advanced gastric cancer (KEYNOTE-012): a multicentre, open-label, phase 1b trial. Lancet Oncol. juin 2016;17(6):717‑26.

38. Doi T, Piha-Paul SA, Jalal SI, Mai-Dang H, Yuan S, Koshiji M, Csiki I, Bennouna J. Pembrolizumab (MK-3475) for patients (pts) with advanced esophageal carcinoma: Preliminary results from KEYNOTE-028. Abstract 4010, ASCO2015

39. El-Khoueiry A, Melero I, Crocenzi T, Welling T, Yau T, Yeo W, et al. Presented at the ASCO; 2015.

40. Schadler KL, Crosby EJ, Zhou AY, Bhang DH, Braunstein L, Baek KH, et al. Immunosurveillance by antiangiogenesis: tumor growth arrest by T cell-derived thrombospondin-1. Cancer Res. 15 avr 2014;74(8):2171‑81.

41. Huang Y, Goel S, Duda DG, Fukumura D, Jain RK. Vascular normalization as an emerging strategy to enhance cancer immunotherapy. Cancer Res. 15 mai 2013;73(10):2943‑8.

42. Voron T, Colussi O, Marcheteau E, Pernot S, Nizard M, Pointet A-L, et al. VEGF-A modulates expression of inhibitory checkpoints on CD8+ T cells in tumors. J Exp Med. 9 févr 2015;212(2):139‑48.

43. Bruix J, Qin S, Merle P, Granito A, Huang YH, Bodoky G, Pracht M, et al. Regorafenib for patients with hepatocellular carcinoma who progressed on sorafenib treatment (RESORCE) : a randomised, double-blind, placebo-controlled, phase 3 trial. Lancet. 2017, Jan 7 ;389(10064) :56-66.

44. Apolo AB, Infante JR, Hamid O, Patel MR, Wang D, Kelly K, et al., Avelumab (MSB0010718C ; anti-PD-L1) in patients with metastatic urothelial carcinoma from the JAVELIN Solid Tumor phase 1b trial : Analysis of safety, clinical activity, and PD-L1 expression. J Clin Oncol 34, 2016 (suppl ; abstr 4514, ASCO2016).

45. Disis ML, Patel MR, Pant S, Hamilton EP, Lockhart AC, Kelly K et al., Avelumab (MSB0010718C ; anti-PD-L1) in patients with recurrent/refractory ovarian cancer from the JAVELIN Solid Tumor phase Ib trial : safety and clinical activity. J Clin Oncol 33, 2015 (suppl ; abstr 5509, ASCO 2016).

46. Schlumberger M, .N Engl J Med. 2015 Feb 12;372, (Brose MS, Lancet. 2014 Jul 26;384)

47. Bastman J Clin Endocrinol Metab. 2016 Jul;101(7):2863-73

48. Severson Cancer Immunol Res. 2015 Jun;3(6):620-30 (Shi RL Thyroid. 2017 Apr;27(4):537-545)

49. Kim S.T., Ha S.Y., et al. The impact of PD-L1 expression in patients with metastatic GEP-NETs. J Cancer 2016, 7 (5) : 484-489

50. van der Graaf WT, Blay JY, Chawla SP et al. Pazopanib for metastatic soft-tissue sarcoma (PALETTE): a randomised, double-blind, placebo-controlled phase 3 trial. Lancet 2012; 379: 1879-1886.

51. D'Angelo SP, Shoushtari AN, Agaram NP et al. Prevalence of tumor-infiltrating lymphocytes and PD-L1 expression in the soft tissue sarcoma microenvironment. Hum Pathol 2014.

52. Kim JR, Moon YJ, Kwon KS et al. Tumor Infiltrating PD1-Positive Lymphocytes and the Expression of PD-L1 Predict Poor Prognosis of Soft Tissue Sarcomas. PLoS One 2013; 8: e82870.

53. Toulmonde M, Adam J, Bessede A et al. Integrative assessment of expression and prognostic value of PDL1, IDO, and kynurenine in 371 primary soft tissue sarcomas with genomic complexity. J Clin Oncol 2016; 34: abstr 11008.

54. Toulmonde M, Penel N, Adam J et al. Use of PD-1 Targeting, Macrophage Infiltration, and IDO Pathway Activation in Sarcomas: A Phase 2 Clinical Trial. JAMA Oncol 2017.

CONFIDENTIAL

55. Qian BZ, Li J, Zhang H et al. CCL2 recruits inflammatory monocytes to facilitate breast-tumour metastasis. Nature 2011; 475: 222-225.

56. Mantovani A, Schioppa T, Porta C et al. Role of tumor-associated macrophages in tumor progression and invasion. Cancer Metastasis Rev 2006; 25: 315-322.

57. Nabeshima A, Matsumoto Y, Fukushi J et al. Tumour-associated macrophages correlate with poor prognosis in myxoid liposarcoma and promote cell motility and invasion via the HB-EGF-EGFR-PI3K/Akt pathways. Br J Cancer 2015; 112: 547-555.

58. Lee CH, Espinosa I, Vrijaldenhoven S et al. Prognostic significance of macrophage infiltration in leiomyosarcomas. Clin Cancer Res 2008; 14: 1423-1430.

59. Jetten N, Verbruggen S, Gijbels MJ et al. Anti-inflammatory M2, but not pro-inflammatory M1 macrophages promote angiogenesis in vivo. Angiogenesis 2014; 17: 109-118.

60. Mao Y, Eissler N, Blanc KL et al. Targeting Suppressive Myeloid Cells Potentiates Checkpoint Inhibitors to Control Spontaneous Neuroblastoma. Clin Cancer Res 2016; 22: 3849-3859.

61. Zhu Y, Knolhoff BL, Meyer MA et al. CSF1/CSF1R blockade reprograms tumor-infiltrating macrophages and improves response to T-cell checkpoint immunotherapy in pancreatic cancer models. Cancer Res 2014; 74: 5057-5069.

62. Shiono et al. Thoracic Cancer 2019

63. Florent Petitprez, Tom Wei-Wu Chen, Cheng-Ming Sun, Julien Calderaro, Li-Ping Hsiao, Laetitia Lacroix, Ivo Natario, Maud Toulmonde, Carlo Lucchesi, Yec'han Laizet, Antoine Italiano, Aurélien de Reyniès, Catherine Sautès-Fridman, Wolf H. Fridman. A novel transcriptomic-based immune classification of soft tissue sarcoma (STS) and its association with molecular characteristics, clinical outcome and response to therapy [abstract]. In: Proceedings of the American Association for Cancer Research Annual Meeting 2018; 2018 Apr 14-18; Chicago, IL. Philadelphia (PA): AACR; Cancer Res 2018;78(13 Suppl):Abstract nr 4045.

64. Petitprez F et al. B cell signatures are associated with improved sarcoma survival and immunotherapy response. Nature 2019, In Press.

65. Manegold C, Dingemans AMC, Gray JE, et al. The potential of combined immunotherapy and antiangiogenesis for the synergistic treatment of advanced NSCLC. J Thorac Oncol. 2017;12(2):194–207.

66. Tao LL, Huang GC, Shi SJ, Chen LB. Bevacizumab improves the antitumor efficacy of adoptive cytokine-induced killer cells therapy in non-small cell lung cancer models. Med Oncol. 2014;31:1. doi:10.1007/s12032-014-0374-0.

67. Zhao S, Jiang T, Li X, Zhou C. Combining anti-angiogenesis and immunotherapy enhances antitumor effect in lung cancer. Ann Oncol. 2016;27:S288–S288.

68. CHOI criteria : Choi H, Charnsangavej C, Faria SC, et al. Correlation of computed tomography and positron emission tomography in patients with metastatic gastrointestinal stromal tumor treated at a single institution with imatinib mesylate: proposal of new computed tomography response criteria. J Clin Oncol 2007;25(13):1753–1759).

CONFIDENTIAL

17. PHARMACOKINETIC, AND ANCILLARY STUDIES

17.1. PHARMACOKINETIC STUDY

17.1.1. Collection of available specimens

Pharmacokinetic of Regorafenib and Avelumab in blood will be assessed on Day 15 of cycle 1, Day 1 and Day 15 of cycle 2, in patients treated in the phase I. The main objective of the pharmacokinetic study is to assess the impact of Avelumab on the PK of Regorafenib.PK through values of Regorafenib and Avelumab will be monitored at selected timepoints: at initial administration (C1D15) and after repeated dosing, at the higher theoretical point (C2D15).
For all patients included in the phase I trial, blood samples will be collected at predefined time points as detailed in table below :

Before the perfusion:

Before the perfusion	**H-30 min**
C1D15	X
C2D1	X
C2D15	X

17.1.2. PK analysis

Metabolites concentration of regorafenib (M-2 and M-5) will be monitored.

17.1.3. Site performing PK study

This study will be performed at Centre Hospitalier Pellegrin-Tripode, Bordeaux.

17.1.4. Shipping of specimens

The container containing the samples will be labelled with coded numbers to ensure full compliance with privacy policy. Samples will be grouped in each institution and sent frozen for centralized processing to:

Pr M. MOLIMARD
Centre Hospitalier Pellegrin Tripode
Laboratoire de pharmacologie clinique et toxicologique.
Plateau technique - 2ème étage 33076 Bordeaux
Tel 05 56 79 59 91 - Fax 05 56 79 47 95

Shipping will only be performed by a sponsor authorized transporter with respect to good practice.

17.2. BIOMARKER STUDY

17.2.1. Collection of Specimen(s)

The main objective of this biomarker study is to explore pharmacodynamics and mechanisms of action of Regorafenib + Avelumab as well as potential predictive biomarkers.

- For each patient treated in the phase I and phase II trial, blood samples will be collected at predefined time points (pre-dose).

	**H**
C1D1	X
C2D1	X
C4D1	X
C6D1	X
Progression	X

The samples will be handled and stored until analysis as described in the Pharmacodynamics Methods Guidelines provided by the sponsor as a separate document.

- Only for consented patient (optional) treated in the phase II trial, stool sample will be collected for the microbiota only at C1D1 (pre-dose).

The samples will be handled as described in the Pharmacodynamics Methods Guidelines provided by the sponsor as a separate document.

CONFIDENTIAL

17.2.2. *Rational*

The translational part of the REGOMUNE study aims to identify through a multi-parametric approach and rigorous bioinformatics and statistical analyses, predictive biomarkers of immunotherapy based on genetic, immunological and metabolomic profiling of immune and tumor cells. Beyond a more comprehensive understanding of the molecular mechanisms involved in immunotherapy sensitivity and resistance, we believe that only an integrated analysis assessing genetic, immunologic and metabolic determinants of the tumor microenvironment will help to identify robust biomarkers that may have very practical implications.
We will perform an integrated analysis of blood samples and tumor biopsies on sequential antiangiogenic and PDL-1 blockade to reveal markers of response and resistance.

17.2.2.1. Blood samples

- *Functional analysis & cytokine measurement*

Wether ICI are able to elicit a functional immune response relies on several interconnected mechanisms which are – when possible – complicated to address. However, upon ICI treatment, the engagement of all these pathways would result in a functional response that should translate into a T cell proliferation and cytokine production. We propose here to evaluate the production of key cytokines such as IFNγ, IL2, TNFα, IL17A, and IL22 (ELISA). Cytokines such as IFNγ, IL2 or TNFα are more related to the immune cell proliferation fate while IL17A or IL22 are related to a particular differentiation status. Indeed, these Th17 / Th22 related cytokines are known to play key pathogenic roles in several inflammatory disorders including psoriasis, multiple sclerosis, rheumatoid arthritis or hashimoto’s thyroiditis.As ICI treatment with either CTLA4 or PD1 blockade is known to be associated with a unique set of toxicty events (Villadolid & Amin, Transl Lung Cancer Res, 2015) with exaggerated inflammatory features, we thus propose to investigate these key to evaluate their possible correlation with immune related Adverse events (irAEs) (Cousin & Italiano, Clin Cancer Res, 2016). Additionally, we propose to evaluate the immune-profiling of PBMCs at different time point as part of ex-vivo reactivity of the immune cells.

- *The Kynurenine pathway hypothesis*

Upon immunotherapy, an anti-tumoral immune response is characterized by an inflammatory response against the tumor. In general this immune response leads to the production of interferon-gamma (IFN□), a cytokine well known to induce the Indoleamine 2,3 dioxygenase (Ido1) enzyme, which aims at, as a negative feedback, repressing the immune response. Ido1 catabolizes tryptophan to form L-Kynurenine and a series of metabolites collectively known as Kynurenines. Certain of these metabolites exert immunoregulatory properties in order to prevent hyper-inflammatory response and its related damage. Especially, L-Kynurenine has been recently shown to limit anti-tumoral immune response in brain tumors (Opitz et al, Nature, 2012) but also to limit acute inflammatory response (Bessede et al, Nature, 2014). While Ido1 is overexpressed in several types of tumor including metastatic melanoma (Brody et al, Cell Cycle, 2009) its expression was described to be elicited by CD8(+) T cells infiltrating the tumor bed (Spranger et al, Sci Transl med, 2013). Going along with this finding, Holmgaard et al, recently shown that Ido1 is also involved in ICI resistance (anti-CTLA4, anti-PD1/PDL1) (Holmgaard et al, JEM, 2013). Also, preliminary investigations we performed shown that anti-CTLA4 treatment induced a substantial increase in L-Kynurenine level in the plasma of tumor bearing mice, which correlate with tumor response (unpublished data). Also, a preliminary investigation in the PEMBROSARC study revealed that pembrolizumab was able to induce a massive change in plasmatic Kynurenine level as seen by the change in Kynurenine to Tryptophan ratio (Figure 3, Toulmonde et al. Jama Oncol In Press). Altogether, these data suggest a critical role of Tryptophan metabolism in the ICI response and that a fine mapping of the Kynurenine pathway could therefore offer a novel approach to predict response to ICI.

CONFIDENTIAL

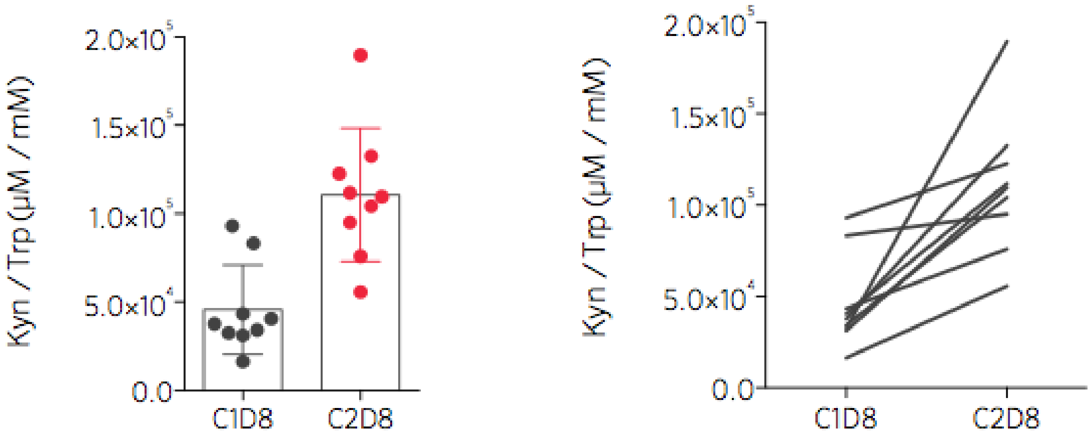

Figure 6: Significant increase of the Kynurenine plasma level in sarcoma patients included in the PEMBROSARC study (day 8 of cycle 1: first infusion of pembrolizumab)

- *Metabolomics*

Given the importance of metabolism in the tumor immune microenvironment, we will perform serum metabolomics by using in performed liquid chromatography-mass spectrometry on pre- and on-treatment serum samples We will precisely measure:

- Arginine metabolism : Arginine, Ornithine, Spermidine
- Adenosine related metabolites : Adenosine, Inosine
- Glutamine related metabolites: Glutamine, Glutamate
- Energy related metabolites: Glucose, Lactate, Succinate, Fumarate

and will correlate these results with best overall response as well as progression-free survival (PFS).

17.2.2.2. Tumor

17.2.2.3. Archived tumor

Archived tumor tissue will be collected for assessment of the tumor microenvironment.

17.2.2.4. Tumor Biopsies

Serial tumor biopsies will be performed to assess changes in the tumor microenvironment from baseline and on Day 1 of cycle 2 (after 4 weeks of treatment). Mutational load, TILs by histopathological assessment, and immune gene profiles in tumors will be evaluated. PD-L1 expression in tumor tissue is not required for enrollment but will be assessed as a predictive marker. The potential role of the gut microbiome in modulating the immune response will also be evaluated by 16S rRNA.
One half of the specimen will be formalin fixed and paraffin embedded [FFPE (Formalin-Fixed Paraffin-Embedded)] and the second half will be fresh frozen at -80°C.
The samples will be labelled with coded numbers to ensure full compliance with privacy policies. Samples will be grouped in each institution and sent for centralized processing with the documents.
All samples will be stored before they are analyzed.
The sample collection information must be captured on the appropriate CRF page(s).

- *Tumor microenvironment*

Fresh pre treatment biopsies will be collected to assess pharmacodynamics biomarkers. Formalin-fixed, paraffin-embedded biopsy samples will be analysed for (but not limited to) CD4,CD8, PDL1, CSF-1R, CD68/CD163, CD68/MHC class II, and other exploratory markers.

- Mutational Load: We will perfume on all pre-treatment tumor samples whole-exome sequencing in order to identify :
- Differences in point mutation and indel status in driver genes between responders and non-responders
- Differences in mutational load between responders and non-responders
- Differences in copy number alterations between responders and non-responders

- *Immune gene profiling*

We will investigate the association between TCR clonality and immune activation in the tumor microenvironment. To do so, we will first calculate the immune score from gene expression profiling data by using the nanostring technology with a custom codeset of 795 genes. We will look for a correlation

CONFIDENTIAL

between TCR clonality and immune scores in pre–immunogenic chemotherapy samples, as well as a correlation between TCR clonality and immune scores in post–combination treatment samples.

17.2.2.5. MICROBIOTE PROFILING

The potential role of the gut microbiome in modulating the immune response will also be evaluated by 16S rRNA.

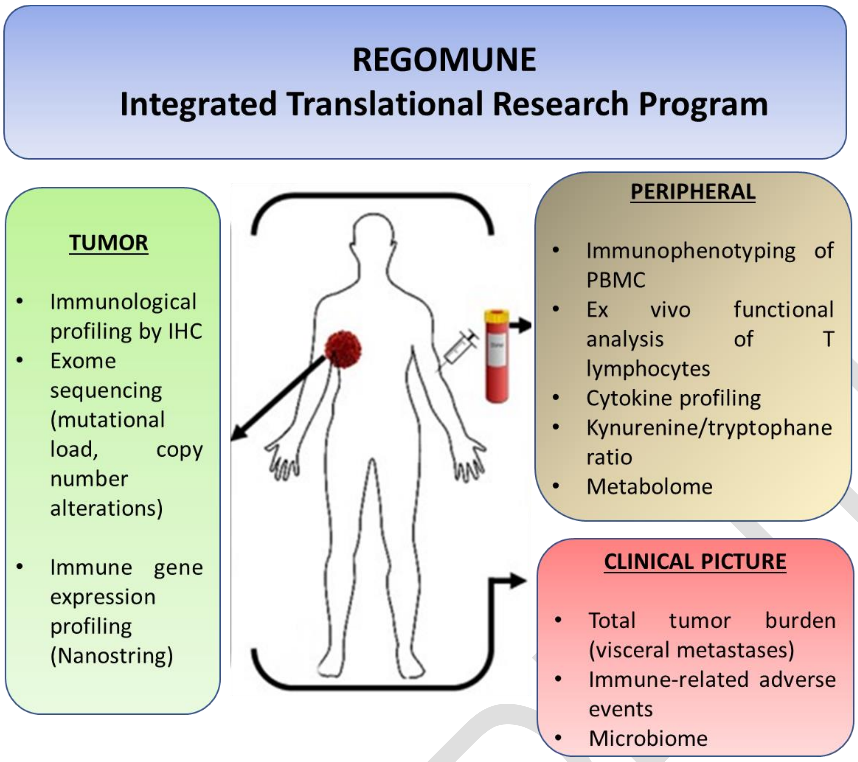

Altogether, both angiogenic and immune markers will be explored in this translational study in order to explore potential surrogate biomarkers for activity or efficacy.
A separate document includes a protocol for sample collection and methods of analysis. (Pharmacodynamics Methods Guidelines will be provided by the sponsor as a separate document).

17.3. ANCILLARY STUDY

17.3.1. Collection of Specimen(s)

Tumor biopsies will be performed on consented adult patients at baseline and on Day 1 of cycle 2 (after 4 weeks of treatment).

17.3.2. Handling and shipping of Specimen(s)

One half of the specimen will be formalin fixed and paraffin embedded [FFPE (Formalin-Fixed Paraffin-Embedded)] and the second half will be fresh frozen at -80°C.
The samples will be labelled with coded numbers to ensure full compliance with privacy policies. Samples will be grouped in each institution and sent for centralized processing with the documents.
All samples will be stored before they are analyzed.
The sample collection information must be captured on the appropriate CRF page(s).

17.3.3. Ancillary analysis

Tumor biopsies will be collected to assess pharmacodynamics changes of TAM infiltration and additional tumor markers and will be analyzed for:

- Hematoxylin and eosin staining (H&E),

Immunohistochemistry (IHC) assessments included, but are not limited to the following markers: VEGFR, PDGFR, HIF1alpha expression, lymphocyte and other exploratory markers.The analysis will be prioritized based on the amount of material available.

- Exome sequencing will be performed in all available patient cases to assess the mutational load.

CONFIDENTIAL

APPENDIX 1: ECOG PERFORMANCE STATUS ASSESSMENT SCALE

Grade	Activity
**0**	Able to carry on all normal activities without restriction.
**1**	Restricted in physically strenuous activity but ambulatory and able to carry out light work.
**2**	Ambulatory and capable of all self-care but unable to carry out any work activities. Up and about more than 50% of waking hours.
**3**	Capable of only limited self-care, confined to bed or chair more than 50% of waking hours.
**4**	Completely disabled. Cannot carry on any self-care. Totally confined to bed or chair.

CONFIDENTIAL

APPENDIX 2: MDRD FORMULA

$$\text{Creatinine clearance (ml/min)} = \frac{[(140\text{-age (years)}) \times \text{weight (Kg)}]}{72 \times \text{serum creatinine (mg/dl)}} \times G^1$$

[1]G (Gender) = 0.85 if Female; 1 if Male

Reference: *Cockcroft, DW, Gault, H. Prediction of creatinine clearance from serum creatinine. Nephron 1976; 16(1):31-41 [84].*

CONFIDENTIAL

APPENDIX 3: EVALUATION OF RESPONSE. THE RECIST

Response and progression will be evaluated in this study using the new international criteria proposed by the revised Response Evaluation Criteria in Solid Tumors (RECIST) guideline (version 1.1) [*Eur J Ca* 45:228-247, 2009]. Changes in only the largest diameter (unidimensional measurement) of the tumor lesions are used in the RECIST criteria.

DEFINITIONS

Evaluable for toxicity: All patients will be evaluable for toxicity from the time of their first treatment with study drugs.

Evaluable for objective response: Only those patients who have measurable disease present at baseline, have received at least one cycle of therapy, and have had their disease re-evaluated will be considered evaluable for response. These patients will have their response classified according to the definitions stated below. (Note: Patients who exhibit objective disease progression prior to the end of cycle 1 will also be considered evaluable.)

DISEASE PARAMETERS

Measurable disease. Measurable lesions are defined as those that can be accurately measured in at least one dimension (longest diameter to be recorded) as ≥20 mm by chest x-ray, as ≥10 mm with CT scan, or ≥10 mm with calipers by clinical exam. All tumor measurements must be recorded in millimeters (or decimal fractions of centimeters).

Note: Tumor lesions that are situated in a previously irradiated area are not considered measurable.

Malignant lymph nodes. To be considered pathologically enlarged and measurable, a lymph node must be ≥15 mm in short axis when assessed by CT scan (CT scan slice thickness recommended to be no greater than 5 mm). At baseline and in follow-up, only the short axis will be measured and followed.

Non-measurable disease. All other lesions (or sites of disease), including small lesions (longest diameter <10 mm or pathological lymph nodes with ≥ 10 to <15 mm short axis), are considered non-measurable disease. Bone lesions, leptomeningeal disease, ascites, pleural/pericardial effusions, lymphangitis cutis/pulmonitis, inflammatory breast disease, and abdominal masses (not followed by CT or MRI), are considered as non-measurable.

Note: Cystic lesions that meet the criteria for radiographically defined simple cysts should not be considered as malignant lesions (neither measurable nor non-measurable) since they are, by definition, simple cysts.

'Cystic lesions' thought to represent cystic metastases can be considered as measurable lesions, if they meet the definition of measurability described above. However, if non-cystic lesions are present in the same patient, these are preferred for selection as target lesions.

Target lesions. All measurable lesions up to a maximum of 2 lesions per organ and 5 lesions in total, representative of all involved organs, should be identified as **target lesions** and recorded and measured at baseline. Target lesions should be selected on the basis of their size (lesions with the longest diameter), be representative of all involved organs, but in addition should be those that lend themselves to reproducible repeated measurements. It may be the case that, on occasion, the largest lesion does not lend itself to reproducible measurement in which circumstance the next largest lesion which can be measured reproducibly should be selected. A sum of the diameters (longest for non-nodal lesions, short axis for nodal lesions) for all target lesions will be calculated and reported as the baseline sum diameters. If lymph nodes are to be included in the sum, then only the short axis is added into the sum. The baseline sum diameters will be used as reference to further characterize any objective tumor regression in the measurable dimension of the disease.

Non-target lesions. All other lesions (or sites of disease) including any measurable lesions over and above the 5 target lesions should be identified as **non-target lesions** and should also be recorded at baseline. Measurements of these lesions are not required, but the presence, absence, or in rare cases unequivocal progression of each should be noted throughout follow-up.

CONFIDENTIAL

METHODS FOR EVALUATION OF MEASURABLE DISEASE

All measurements should be taken and recorded in metric notation using a ruler or calipers. All baseline evaluations should be performed as closely as possible to the beginning of treatment and never more than 4 weeks before the beginning of the treatment.

The same method of assessment and the same technique should be used to characterize each identified and reported lesion at baseline and during follow-up. Imaging-based evaluation is preferred to evaluation by clinical examination unless the lesion(s) being followed cannot be imaged but are assessable by clinical exam.

Conventional CT and MRI: This guideline has defined measurability of lesions on CT scan based on the assumption that CT slice thickness is 5 mm or less. If CT scans have slice thickness greater than 5 mm, the minimum size for a measurable lesion should be twice the slice thickness. MRI is also acceptable in certain situations (e.g. for body scans).

RESPONSE CRITERIA

Evaluation of Target Lesions

Complete Response (CR): Disappearance of all target lesions. Any pathological lymph nodes (whether target or non-target) must have reduction in short axis to <10 mm.

Partial Response (PR): At least a 30% decrease in the sum of the diameters of target lesions, taking as reference the baseline sum diameters.

Progressive Disease (PD): At least a 20% increase in the sum of the diameters of target lesions, taking as reference the smallest sum on study (this includes the baseline sum if that is the smallest on study). In addition to the relative increase of 20%, the sum must also demonstrate an absolute increase of at least 5 mm. (Note: the appearance of one or more new lesions is also considered progressions).

Stable Disease (SD): Neither sufficient shrinkage to qualify for PR nor sufficient increase to qualify for PD, taking as reference the smallest sum diameters while on study

Evaluation of Non-Target Lesions

Complete Response (CR): Disappearance of all non-target lesions and normalization of tumor marker level. All lymph nodes must be non-pathological in size (<10 mm short axis)

Note: If tumor markers are initially above the upper normal limit, they must normalize for a patient to be considered in complete clinical response.

Non-CR/Non-PD: Persistence of one or more non-target lesion(s) and/or maintenance of tumor marker level above the normal limits

Progressive Disease (PD): Appearance of one or more new lesions and/or unequivocal progression of existing non-target lesions.

Although a clear progression of “non-target” lesions only is exceptional, the opinion of the treating physician should prevail in such circumstances, and the progression status should be confirmed at a later time by the review panel (or Principal Investigator).

CONFIDENTIAL

Definition of the Best Response

The best response determination in trial where confirmation of complete or partial response is required:

Complete or partial responses may be claimed only if the criteria for each are met at a subsequent time point as specified in the protocol (**generally 4 weeks later**). In this circumstance, the best overall response can be interpreted as in Table below.

Table 3 – Best overall response when confirmation of CR and PR required.

Overall response<br>First time point	Overall response<br>Subsequent time point	BEST overall response
CR	CR	CR
CR	PR	SD, PD or PR[a]
CR	SD	SD provided minimum criteria for SD duration met, otherwise, PD
CR	PD	SD provided minimum criteria for SD duration met, otherwise, PD
CR	NE	SD provided minimum criteria for SD duration met, otherwise NE
PR	CR	PR
PR	PR	PR
PR	SD	SD
PR	PD	SD provided minimum criteria for SD duration met, otherwise, PD
PR	NE	SD provided minimum criteria for SD duration met, otherwise NE
NE	NE	NE

CR = complete response, PR = partial response, SD = stable disease, PD = progressive disease, and NE = inevaluable.
a If a CR is truly met at first time point, then any disease seen at a subsequent time point, even disease meeting PR criteria relative to baseline, makes the disease PD at that point (since disease must have reappeared after CR). Best response would depend on whether minimum duration for SD was met. However, sometimes 'CR' may be claimed when subsequent scans suggest small lesions were likely still present and in fact the patient had PR, not CR at the first time point. Under these circumstances, the original CR should be changed to PR and the best response is PR.

Special notes on response assessment

When nodal disease is included in the sum of target lesions and the nodes decrease to 'normal' size (<10 mm), they may still have a measurement reported on scans. This measurement should be recorded even though the nodes are normal in order not to overstate progression should it be based on increase in size of the nodes. As noted earlier, this means that patients with CR (complete response) may not have a total sum of 'zero' on the case report form (CRF).

In trials where confirmation of response is required, repeated 'NE' time point assessments may complicate best response determination. The analysis plan for the trial must address how missing data/assessments will be addressed in determination of response and progression. For example, in most trials it is reasonable to consider a patient with time point responses of PR-NE-PR as a confirmed response.

Patients with a global deterioration of health status requiring discontinuation of treatment without objective evidence of disease progression at that time should be reported as 'symptomatic detioration'. Every effort should be made to document objective progression even after discontinuation of treatment. Symptomatic deterioration is *not* a descriptor of an objective response: it is a reason for stopping study therapy. The objective response status of such patients is to be determined by evaluation of target and non-target disease as shown in Tables belows.

APPENDIX 4: PATIENT MEDICATION DIARY

Notice d'utilisation des médicaments à l'étude

Regorafenib

- ✓ 1 prise par jour à heure fixe : pendant 3 semaines
- ✓ à prendre avec un verre d'eau
- ✓ à prendre après un repas léger pauvre en graisse. Exemple de repas léger : une portion de céréales (environ 30 g), un verre de lait écrémé, une tranche de pain avec de la confiture, un verre de jus de pomme et une tasse de café ou de thé (520 calories, 2 g de lipides).
- ✓ les comprimés doivent être avalés entiers
- ✓ 1 semaine sans traitement

Avelumab

- ✓ 1 injection au J15 du cycle 1, à l'hôpital
- ✓ 1 injection aux J1 et J15 de chaque cycle, à l'hôpital

En cas d'oubli

- Si rappel dans la journée, prendre le comprimé de Regorafenib
- Sinon, ne pas doubler la dose suivante

En cas de vomissements

- Ne pas reprendre la dose, et ne pas doubler la dose suivante

Conservation du traitement

- o Conservation à température ambiante inférieure à 25°C.
- o Ne pas utiliser après la date de péremption figurant sur la boite
- o Tenir hors de portée des enfants

PROTOCOLE

REGOMUNE

Carnet patient

De Mme/Mr...

Cycle N° |__|__|

Madame, Monsieur,

Dans le cadre de votre participation à l'étude Regomune, il est nécessaire d'avoir des informations sur le suivi de votre traitement par **Regorafenib** et **Avelumab**.

Vous trouverez dans ce carnet un tableau à compléter chaque jour, et nous vous remercions d'y noter :

- ✓ la date, et le nombre de comprimés de Regorafenib pris par jour
- ✓ d'annoter un commentaire si nécessaire (effets secondaires par exemple)
- ✓ l'indication de non prise des traitements s'il y a lieu en cochant la case correspondante et en complétant d'un commentaire la raison.

N'oubliez pas de remettre ce livret à l'Infirmièr(e) de Recherche Clinique, Attaché(e) de Recherche Clinique ou Pharmacie hospitalière lors de votre prochain rendez-vous.

Nous vous remercions de votre précieuse collaboration.

REGOMUNE – Version 1.0 -24.10.2017- 1

Date		Regorafenib		Avelumab	Commentaires
			Non pris		
**J1**	___/____/___	|___| cp	☐	☐ (NA pour le cycle 1)	
**J2**	___/____/___	|___| cp	☐		
**J3**	___/____/___	|___| cp	☐		
**J4**	___/____/___	|___| cp	☐		
**J5**	___/____/___	|___| cp	☐		
**J6**	___/____/___	|___| cp	☐		
**J7**	___/____/___	|___| cp	☐		
**J8**	___/____/___	|___| cp	☐		
**J9**	___/____/___	|___| cp	☐		
**J10**	___/____/___	|___| cp	☐		
**J11**	___/____/___	|___| cp	☐		
**J12**	___/____/___	|___| cp	☐		
**J13**	___/____/___	|___| cp	☐		
**J14**	___/____/___	|___| cp	☐		

Date		Regorafenib		Avelumab	Commentaires
			Non pris		
**J15**	___/____/___	|___| cp	☐	☐	
**J16**	___/____/___	|___| cp	☐		
**J17**	___/____/___	|___| cp	☐		
**J18**	___/____/___	|___| cp	☐		
**J19**	___/____/___	|___| cp	☐		
**J20**	___/____/___	|___| cp	☐		
**J21**	___/____/___	|___| cp	☐		
**J22**	___/____/___				
**J23**	___/____/___				
**J24**	___/____/___				
**J25**	___/____/___				
**J26**	___/____/___				
**J27**	___/____/___				
**J28**	___/____/___				

APPENDIX 5: SERIOUS ADVERSE EVENT NOTIFICATION FORM

Serious Adverse Event Notification Form

TO BE FAXED TO THE UNICANCER VIGILANCE UNIT– N° + 33 (0)1.44.23.55.70

PROTOCOL : REGOMUNE	EUDRACT/ ID-RCB N°: 2016-005175-27	COUNTRY: France
SPONSOR IDENTIFICATION N°:	INVESTIGATOR SITE :	SITE N°:
DATE OF THIS REPORT: |__|__||__|__||__|__|__|__|	INITIAL REPORT ☐ / FOLLOW-UP REPORT N°|__|__|	FINAL REPORT ☐

1. PATIENT IDENTIFICATION

INCLUSION N°: \|______\| SURNAME (1 LETTER): \|__\| 1ST NAME (1 LETTER): \|__\| DATE OF BIRTH (MM/AAAA): \|__\|__\|\|__\|__\|__\|__\| TREATMENT ARM: \|__\|

DOSE LEVEL (PHASE I STUDY): \|__\|

GENDER: F ☐ M ☐ WEIGHT (KG): \|__\|__\|__\| HEIGHT (CM): \|__\|__\|__\| BODY SURFACE AREA (M²): \|__\| . \|__\|__\|

2. INFORMATION ON EVENT

DATE OF ONSET: \|__\|__\|\|__\|__\|\|__\|__\|__\|__\| TOXICITY (NCI–CTC GRADE): 1 ☐ 2 ☐ 3 ☐ 4 ☐ 5 ☐

DIAGNOSIS OR MAIN *SYMPTOM*: **only one diagnosis or one symptom** (except for linked symptoms) ....

....

....

DESCRIBE EVENT AND TREATMENT GIVEN (INCLUDING RELEVANT TEST/LAB DATA): ....

....

....

3. SERIOUSNESS CRITERIA	4. OUTCOME
☐ DEATH, DATE OF DEATH: ......|__|__||__|__||__|__|__|__| ☐ LIFE-THREATENING<br>☐ REQUIRING/PROLONGING HOSPITALIZATION:<br>DATE OF ADMISSION: ....|__|__||__|__||__|__|__|__| ☐ PERSISTENT/SIGNIFICANT DISABILITY/INCAPACITY<br>☐ CONGENITAL DISORDER/BIRTH DEFECT<br>☐ MEDICALLY RELEVANT	☐ ONGOING EVENT ☐ UNKNOWN OUTCOME<br>☐ RECOVERED WITHOUT SEQUELAE, ☞ DATE |__|__||__|__||__|__|__|__| ☐ RECOVERED WITH SEQUELAE, ☞ DATE |__|__||__|__||__|__|__|__| SPECIFY SEQUELAE: ....<br>..<br>☐ DEATH RELATED TO THIS EVENT, ☞ DATE |__|__||__|__||__|__|__|__| ☐ DEATH UNRELATED TO THIS EVENT, ☞ DATE |__|__||__|__||__|__|__|__| CAUSE OF DEATH:..<br>..<br>*OR* CAUSE UNKNOWN ☐<br>☐ AUTOPSY: YES ☐ NO ☐<br><br>**IF PATIENT WAS HOSPITALIZED:** DATE OF END OF HOSPITALIZATION: |__|__||__|__||__|__|__|__| *OR* PATIENT STILL HOSPITALIZED AT TIME OF THIS REPORT ☐

FOR IMP TRIALS ⇨ COMPLETE SECTION 5 FOR RADIOTHERAPY TRIALS ⇨ COMPLETE SECTION 6 FOR OTHER NON IMP TRIALS ⇨ COMPLETE SECTION 7

5. IMP (INVESTIGATIONAL MEDICINAL PRODUCT(S), including combined RADIOTHERAPY / SURGERY...)..............TICK IF NA ☐

INVESTIGATIONAL PROCEDURE(S) INDICATE THE INTERNATIONAL COMMON DENOMINATION OF THE IMP & OTHER COMBINED	ROUTE	SAE CYCLE NUMBER	DATES: DATE OF FIRST ADMINISTRATION/USE (1ST DAY OF 1ST CYCLE)	DATES: DATE OF LAST ADMINISTRATION/USE BEFORE SAE	DOSE & UNIT: LAST DOSE ADMINISTERED BEFORE SAE – DOSE	LAST DOSE ADMINISTERED BEFORE SAE – UNIT	CUMULATIVE DOSE SINCE THE 1ST ADMINISTRATION – DOSE	CUMULATIVE DOSE SINCE THE 1ST ADMINISTRATION – UNIT
1.			|__|__||__|__||__|__|__|__|	|__|__||__|__||__|__|__|__|				
2.			|__|__||__|__||__|__|__|__|	|__|__||__|__||__|__|__|__|				
3.			|__|__||__|__||__|__|__|__|	|__|__||__|__||__|__|__|__|				
4.			|__|__||__|__||__|__|__|__|	|__|__||__|__||__|__|__|__|				
5.			|__|__||__|__||__|__|__|__|	|__|__||__|__||__|__|__|__|				
6			|__|__||__|__||__|__|__|__|	|__|__||__|__||__|__|__|__|				
7.			|__|__||__|__||__|__|__|__|	|__|__||__|__||__|__|__|__|				

UNBLINDING : YES ☐ NO ☐ NA ☐

HAS ONE (OR SEVERAL) INVESTIGATIONAL PRODUCT(S) BEEN STOPPED?
☐ YES N°\|__\| N°\|__\| N°\|__\| N°\|__\| N°\|__\| N°\|__\| N°\|__\| ☐ NO ☐ NA

HAS ONE (OR SEVERAL) INVESTIGATIONAL PRODUCT(S) BEEN REINTRODUCED?
☐ YES N°\|__\| N°\|__\| N°\|__\| N°\|__\| N°\|__\| N°\|__\| N°\|__\| ☐ NO ☐ NA

DID THE EVENT DISAPPEAR AFTER INVESTIGATIONAL PRODUCT(S) IS STOPPED?
☐ YES ☐ NO ☐ NA

DID THE EVENT REAPPEAR AFTER INVESTIGATIONAL PRODUCT(S) REINTRODUCTION?
☐ YES ☐ NO ☐ NA

Serious Adverse Event Notification Form

TO BE FAXED TO THE UNICANCER VIGILANCE UNIT – N° + 33 (0)1.44.23.55.70

PROTOCOL : REGOMUNE EUDRACT/ ID-RCB N°: 2016-005175-27		COUNTRY: France
SPONSOR IDENTIFICATION N°:	INVESTIGATOR SITE :	SITE N°:
DATE OF THIS REPORT: |__|__|____|	INITIAL REPORT ☐ FOLLOW-UP REPORT N°|__|	FINAL REPORT ☐
INCLUSION N°: |____| SURNAME (1 LETTER): |_| 1ST NAME (1 LETTER): |_| DATE OF BIRTH (MM/AAAA): |__|____|		

6. RADIOTHERAPY .... TICK IF NA ☐

TECHNIQUE	FIELD(S)	DATES		DOSE (GY)	
		DATE OF FIRST ADMINISTRATION	DATE OF LAST ADMINISTRATION	LAST DOSE ADMINISTERED BEFORE SAE (GY)	CUMULATIVE DOSE SINCE THE 1ST ADMINISTRATION (GY)
		|__|__|____|	|__|__|____|		
		|__|__|____|	|__|__|____|		
		|__|__|____|	|__|__|____|		
		|__|__|____|	|__|__|____|		

MACHINE (SPECIFY IF POSSIBLE TRADE NAME / MODEL/SERIAL NUMBER): ....

....

HAS RADIOTHERAPY BEEN STOPPED?<br>☐ YES ☐ NO ☐ NA<br>DID THE EVENT DISAPPEAR AFTER RADIOTHERAPY IS STOPPED?<br>☐ YES ☐ NO ☐ NA	HAS RADIOTHERAPY BEEN REINTRODUCED?<br>☐ YES ☐ NO ☐ NA<br>DID THE EVENT REAPPEAR AFTER RADIOTHERAPY REINTRODUCTION?<br>☐ YES ☐ NO ☐ NA

SCENE OF EVENT: ☐ INVESTIGATOR SITE ☐ HOME ☐ HOSPITAL ☐ DAY HOSPITAL ☐ CONVALESCENT HOME

☐ OTHER: ....

7. MEDICAL DEVICE or NON MEDICINAL PRODUCT, METHOD or ACTION .... TICK IF NA ☐

DEVICE / Non Medicinal Product, Method or Action	DATES OF USE
COMMON DENOMINATION :<br><br>TRADE NAME (IF EC MARKING) :	START DATE: |__|__|____| STOP DATE: |__|__|____|
MODEL :	VERSION (INCLUDED SOFTWARE) :
SERIAL NUMBER :	AND/OR BATCH NUMBER :
INDICATION OF USE FOR THE PATIENT :	

HAS DEVICE OR ONE (OR SEVERAL) PRODUCTS(S), METHOD(S) OR ACTION(S) BEEN STOPPED?<br>☐ YES ☐ NO ☐ NA<br>DID THE EVENT DISAPPEAR AFTER STOP?<br>☐ YES ☐ NO ☐ NA	HAS DEVICE OR ONE (OR SEVERAL) PRODUCT(S), METHOD(S) OR ACTION(S) BEEN REINTRODUCED?<br>☐ YES ☐ NO ☐ NA<br>DID THE EVENT REAPPEAR AFTER REINTRODUCTION?<br>☐ YES ☐ NO ☐ NA

SCENE OF EVENT: ☐ INVESTIGATOR SITE ☐ HOME ☐ HOSPITAL ☐ DAY HOSPITAL ☐ CONVALESCENT HOME

☐ OTHER, SPECIFY: ....

Serious Adverse Event Notification Form

TO BE FAXED TO THE UNICANCER VIGILANCE UNIT–N° + 33 (0)1.44.23.55.70

PROTOCOL :REGOMUNE	EUDRACT/ ID-RCB N°:2016-005175-27	COUNTRY: France
SPONSOR IDENTIFICATION N°:	INVESTIGATOR SITE :	SITE N°:
DATE OF THIS REPORT: |__|__|____|	INITIAL REPORT ☐ FOLLOW-UP REPORT N°|__|	FINAL REPORT ☐
INCLUSION N°: |____| SURNAME (1 LETTER): |_| 1ST NAME (1 LETTER): |_|	DATE OF BIRTH (MM/AAAA) : |__|____|	

8. CONCOMITANT DRUG(S) – (EXCLUDE THOSE USED TO TREAT REACTION)

CONCOMITANT DRUG	ROUTE	START DATE	STOP DATE	ONGOING	INDICATION
1.		FROM |__|__|__|	TO |__|__|__|	☐	
2.		FROM |__|__|__|	TO |__|__|__|	☐	
3.		FROM |__|__|__|	TO |__|__|__|	☐	
4.		FROM |__|__|__|	TO |__|__|__|	☐	

9. OTHER RELEVANT HISTORY (E.G. DIAGNOSTICS, ALLERGIES, PREGNANCY WITH LAST MONTH OF PERIOD, ETC…)

....

....

....

10. ASSESSMENT: IN YOUR OPINION (INVESTIGATOR), THIS EVENT IS RELATED TO (TICK ONLY ONE BOX):

☐ IMP (INVESTIGATIONAL MEDICINAL PRODUCT(S) INCLUDING COMBINED RADIOTHERAPY / SURGERY)
SPECIFY THE IMP NUMBER(S) (SEE SECTION 5 OF THE FORM): N°\|_\| N°\|_\| N°\|_\| N°\|_\| N°\|_\| N°\|_\| N°\|_\|

☐ INVESTIGATIONAL RADIOTHERAPY,

☐ INVESTIGATIONAL MEDICAL DEVICE OR NON MEDICINAL PRODUCT, METHOD OR ACTION

IF NOT RELATED TO EITHER INVESTIGATIONAL MP / RADIOTHERAPY / SURGERY, NMP, OR MD, **PLEASE SPECIFY** (TICK ONLY ONE BOX)

☐ PROTOCOL

☐ CONCOMITANT TREATMENT(S), SPECIFY: ....

☐ CONCOMITANT DISEASE(S), SPECIFY: ....

☐ OTHER, SPECIFY: ....

11. SAE NOTIFICATED BY:

NAME:
FUNCTION:
ADDRESS:
PHONE: .... FAX:..
E-MAIL: ....
DATE \|__\|/\|__\|/\|____\|

SIGNATURE: ....

INVESTIGATOR

NAME:
DEPARTMENT:

DATE \|__\|/\|__\|/\|____\|

SIGNATURE: ....

SPONSOR ONLY (DO NOT FULFIL THIS PART)

SPONSOR IDENTIFICATION NUMBER: ....
DATE OF RECEIPT: \|__\|/\|__\|/\|____\| DATE OF THIS REPORT: \|__\|/\|__\|/\|____\|

ASSESSMENT (Tick only one box):

1 ☐ INVESTIGATIONAL **MP** (INCLUDING COMBINED RADIOTHERAPY / SURGERY)
�SPECIFY THE NUMBER(S)° N° \|_\| N° \|_\| N° \|_\| N° \|_\| N°\|_\| N°\|_\| N°\|_\|
2 ☐ INVESTIGATIONAL RADIOTHERAPY,
3 ☐ INVESTIGATIONAL MEDICAL DEVICE OR NON MEDICINAL PRODUCT, METHOD OR ACTION

�IS IT A SUSPECTED UNEXPECTED SERIOUS ADVERSE REACTION (**SUSAR**)? YES ☐ NO ☐

IF NOT RELATED TO EITHER 1, 2 OR 3, PLEASE SPECIFY (TICK ONLY ONE BOX)
4 ☐ PROTOCOL
5 ☐ CONCOMITANT TREATMENT(S)
6 ☐ CONCOMITANT DISEASE(S), SPECIFY ....
7 ☐ OTHER, SPECIFY ....

DATE \|__\|/\|__\|/\|____\| NAME .... SIGNATURE : ....

APPENDIX 6: PREGNANCY NOTIFICATION FORM

Bergonié
UNICANCER
NOUVELLE-AQUITAINE

Pregnancy Notification Form

TO BE FAXED TO VIGILANCE UNIT– N° + 33 (0)1.44.23.55.70

PROTOCOL :REGOMUNE	EUDRACT/ ID-RCB N°:2016-005175-27	COUNTRY: France
SPONSOR IDENTIFICATION N°:	INVESTIGATOR SITE :	SITE N°:
DATE OF THIS REPORT: |__|__|__|__|__|__|__|__|	INITIAL REPORT ☐ / FOLLOW-UP REPORT N°|__|__|	FINAL REPORT ☐

THIS NOTIFICATION CONCERNS: ☐ PREGNANCY ☐FEEDING

1. PATIENT IDENTIFICATION

INCLUSION N°: \|______\| AGE (AT TIME OF DISCOVERY): \|__\|__\| YEARS GENDER: FEMALE ☐ MALE ☐

TREATMENT ARM \|_____\| DOSE LEVEL (ONLY FOR PHASE I STUDIES) \|_____\| WEIGHT (Kg): \|__\|__\|__\| HEIGHT (CM): \|__\|__\|__\| BODY SURFACE AREA (M²): \|__\|\|__\|__\|

2. INFORMATION ON PREGNANT

THE PREGNANT IS: THE PATIENT ☐

A PATIENT PARTNER ☐ ☞SPECIFY AGE (AT TIME OF DISCOVERY): \|__\|__\| WEIGHT (Kg): \|__\|__\|__\| HEIGHT (CM): \|__\|__\|__\| BODY SURFACE \|__\|\|__\|__\|

DATE OF LAST MENSTRUAL PERIOD (DD/MM/YYYY): \|__\|__\|\|__\|__\|\|__\|__\|__\|__\| ESTIMATED DATE OF DELIVERY (DD/MM/YYYY): \|__\|__\|\|__\|__\|\|__\|__\|__\|__\|

WAS THE PATIENT USING CONTRACEPTION? YES ☐ SPECIFY:.. NO ☐ UNCERTAIN ☐

DO YOU THINK THERE WAS A FAILURE IN CONTRACEPTION? YES ☐ NO ☐ UNKNOWN ☐

CAUSE/REASON FOR FAILURE (NON-COMPLIANCE, MECHANICAL, DRUG INTERACTION...): ....

MATERNAL PREGNANCY ASSOCIATED EVENTS (if yes, specify): ....

<u>IF THE MOTHER EXPERIENCED A SERIOUS ADVERSE EVENT DURING PREGNANCY, PLEASE COMPLETE A SAE FORM.</u>

3. DRUGS INFORMATION

DESCRIBE ALL RELEVANT TREATMENTS ADMINISTERED TO THE PREGNANT AND HER PARTNER IF APPLICABLE (Date and dose of investigational drugs and concomitant treatments):

CONCERNING PATIENT INCLUDED IN THE STUDY:

INVESTIGATIONAL PROCEDURE(S) INDICATE THE INTERNATIONAL COMMON DENOMINATION OF THE IMP * & OTHER COMBINED AND/OR RADIOTHERAPY AND/OR DEVICE AND/OR METHOD OR ACTION	ROUTE	EVENT CYCLE NUMBER	DATES		DOSES AND UNIT			
			DATE OF FIRST ADMINISTRATION/USE (1st DAY OF 1st CYCLE)	DATE OF LAST ADMINISTRATION/USE (1st DAY OF 1st CYCLE)	LAST DAILY DOSE ADMINISTRATED BEFORE PREGNANCY DISCOVERY		CUMULATIVE DOSE SINCE THE 1st ADMINISTRATION IF APPLICABLE	
					DOSE	UNIT	DOSE	UNIT
1.			|__|__||__|__||__|__|__|__|	|__|__||__|__||__|__|__|__|				
2.			|__|__||__|__||__|__|__|__|	|__|__||__|__||__|__|__|__|				
3.			|__|__||__|__||__|__|__|__|	|__|__||__|__||__|__|__|__|				
4.			|__|__||__|__||__|__|__|__|	|__|__||__|__||__|__|__|__|				
5.			|__|__||__|__||__|__|__|__|	|__|__||__|__||__|__|__|__|				

**IMP: INVESTIGATIONAL MEDICAL PRODUCT*

DOES TREATMENT(S) STOPPED WHEN PREGNANCY WAS DISCOVERED: YES ☐ NO ☐

IF NO EXPLAIN AND GIVE THE STOP DATE FOR EACH TREATMENT OR PROCEDURE : ....
....
....

CONCERNING THE PREGNANT:

CONCOMITANT DRUGS (INN)	ROUTE	DAILY DOSE	START DATE	STOP DATE	CHECK BOX IF ONGOING	INDICATION
1.			|__|__||__|__||__|__|__|__|	|__|__||__|__||__|__|__|__|	☐	
2.			|__|__||__|__||__|__|__|__|	|__|__||__|__||__|__|__|__|	☐	
3.			|__|__||__|__||__|__|__|__|	|__|__||__|__||__|__|__|__|	☐	
4.			|__|__||__|__||__|__|__|__|	|__|__||__|__||__|__|__|__|	☐	
5.			|__|__||__|__||__|__|__|__|	|__|__||__|__||__|__|__|__|	☐	
6.			|__|__||__|__||__|__|__|__|	|__|__||__|__||__|__|__|__|	☐	

4. PARENTS RELEVANT MEDICAL HISTORY:

<u>MOTHER:</u>

RISK FACTORS (TICK IF YES): ☐SMOKING, ☐ALCOHOL, ☐DRUG ABUSE, ☐INFECTION, ☐MEDICATION, ☐HYPERTENSION, ☐ECLAMPSIA ☐OTHERS, Specify:..

RELEVANT MEDICAL HISTORY (include information on familial disorders, known risk factors or conditions that may affect the outcome of the pregnancy):
....
....

UREC-FQ-0108_V002 FORM_REGOMUNE_PREG NOTIFICATION_V2.0 du 18/09/2020

PAGE 1 OF 2

afaq ISO 9001 Qualité AFNOR CERTIFICATION

Bergonié UNICANCER NOUVELLE-AQUITAINE

Pregnancy Notification Form

TO BE FAXED TO VIGILANCE UNIT– N° + 33 (0)1.44.23.55.70

PROTOCOL : REGOMUNE	EUDRACT/ ID-RCB N°: 2016-005175-27	COUNTRY: France
SPONSOR IDENTIFICATION N°:	INVESTIGATOR SITE :	SITE N°:
DATE OF THIS REPORT: |__|__||__|__||__|__|__|__|	INITIAL REPORT ☐ / FOLLOW-UP REPORT N° |__|__|	FINAL REPORT ☐
INCLUSION N°: |________|	AGE (YEARS OLD) : |__|__||__|__|__|__|	

MOTHER:

PREVIOUS OBSTETRIC HISTORY (PROVIDE ALL PREVIOUS PREGNANCIES INCLUDING ABORTION AND STILLBIRTH)

DATE	GESTATION WEEK	OUTCOME
|__|__||__|__||__|__|__|__|		
|__|__||__|__||__|__|__|__|		
|__|__||__|__||__|__|__|__|		
|__|__||__|__||__|__|__|__|		

FATHER:

RELEVANT MEDICAL HISTORY :

....

....

5. PREGNANCY OUTCOME

☐ NOT APPLICABLE AT THE TIME OF DECLARATION ☐ APPLICABLE (COMPLETE BELLOW)

☐ DELIVERY<br>|__|__||__|__||__|__|__|__| (DD/MM/YYYY)	☐ NORMAL<br>☐ FORCEPS/VENTOUSE<br>☐ CAESAREAN	MATERNAL COMPLICATIONS OR PROBLEMS RELATED TO BIRTH<br>..
☐ ABORTION<br>|__|__||__|__||__|__|__|__| (DD/MM/YYYY)	☐ PLANNED<br>☐ THERAPEUTIC<br>☐ SPONTANEOUS	SPECIFY REASONS<br>..

OTHER INFORMATIONS (for exemple in case of not yet delivery provide results of specific tests performed e.g. amniocentesis, ultrasound, maternal serum afp, serology tests etc) :

....

ASSESSMENT OF CAUSALITY: PLEASE INDICATE THE RELATIONSHIP BETWEEN THE PREGNANCY OUTCOME AND IMP:

☐ Not Related ☐ Related, specify IMP:.................................

6. CHILD INFORMATION (if applicable)

NEONATE:

☐ NORMAL ☐ STILLBIRTH ☐ ABNORMAL, SPECIFY: ....

OTHERS INFORMATIONS:

SEX: ☐ MALE ☐ FEMALE HEIGHT: ....CM WEIGHT: ....Kg OTHER: ....

7. INFORMATION ON FEEDING

☐ NOT APPLICABLE

DATE OF FIRST FEEDING (DD/MM/AAAA): \|__\|__\|\|__\|__\|\|__\|__\|__\|__\|

☐ APPLICABLE (COMPLETE BELLOW)

DATE OF LAST FEEDING (DD/MM/AAAA): \|__\|__\|\|__\|__\|\|__\|__\|__\|__\|

DESCRIBE ALL RELEVANT INFORMATION: ....

8. FOLLOW-UP INFORMATION

FOLLOW-UP INFORMATION CAN BE OBTAINED FROM:

DOCTOR: .... INSTITUTION: .... ADDRESS: ....

E-MAIL: .... PHONE: .... FAX: ....

9. INVESTIGATOR

NAME: .... INSTITUTION: ....

E-MAIL: ....

PHONE: ....

FAX: ....

DATE (DD/MM/YYYY): \|__\|__\| / \|__\|__\| / \|__\|__\|__\|__\|: SIGNATURE: ....

APPENDIX 7: DOSE MODIFICATION/DELAY FOR TOXICITIES RELATED TO REGORAFENIB (EXCEPT FOR HAND-FOOT SYNDROME REACTION, HYPERTENSION, AND ALT AND/OR AST AND/OR BILIRUBIN INCREASES)

NCI-CTCAE v5.0	Dose interruption	Dose modification[c]	Dose for subsequent cycles
Grade 0-2	Treat on time	No change	No change
Grade 3	Delay until $\leq$ grade 2[a]	Reduce 1 dose level	No possibility of re-escalation
Grade 4	Delay until $\leq$ grade 2[b]	Reduce 1 dose level<br>Permanent discontinuation must be discussed with the sponsor in case of recurrence.	

a- Excludes alopecia, non-refractory nausea/vomiting, non-refractory hypersensitivy and asymptomatic laboratory abnormalities
b- If no recovery after a 4 weeks delay, treatment will be permanently discontinued.

[c] For cohort [A'], regorafenib should be interrupted and no dose reduction are allowed. Patients requiring a delay of > 4 weeks should permanently discontinue regorafenib.

CONFIDENTIAL

APPENDIX 8: DOSE DELAYS FOR TOXICITIES RELATED TO AVELUMAB AND REGORAFENIB INCLUDED IMMUNE-RELATED TOXICITIES

**Hand-foot skin reactions**<br>Except for irAE skin reaction	**Regorafenib**[a]<br>Management/Dose-modification	**Avelumab**<br>Management/Dose-modification
**Grade 1:** Numbness, dysesthesia, paraesthesia, tingling, painless swelling, erythema or discomfort of the hands or feet which does not disrupt the patient's normal activities.	Any: Maintain dose level and immediately institute supportive measures for symptomatic relief.	No dose modification
**Grade 2:** Painful erythema and swelling of the hands or feet and/or discomfort which affects the patient's normal activities.	1st occurrence:<br>Consider decreasing dose by one dose level and immediately institute supportive measures. If there is no improvement, interrupt therapy for a minimum of 7 days, until toxicity resolves to Grade 0-1.<br><br>No improvement within 7 days or 2nd occurrence:<br>Interrupt therapy until toxicity resolves to Grade 0-1. When resume treatment, treat at reduced dose level<br><br>3th occurrence:<br>Discontinue therapy.	No dose modification
**Grade 3**: Moist desquamation, ulceration, blistering or severe pain of the hands or feet, or severe discomfort that causes the patient to be unable to work or perform activities of daily living.	1st occurrence:<br>Institute support measures immediately. Interrupt therapy for a minimum of 7 days until toxicity resolves to Grade 0-1. When resume treatment, decrease dose by one dose level<br><br>2nd occurrence:<br>Discontinue therapy	No dose modification

[a] For cohort [A'], regorafenib should be interrupted and no dose reduction are allowed. Patients requiring a delay of > 4 weeks should permanently discontinue regorafenib

CONFIDENTIAL

**Hypertension**	**Regorafenib**[a]<br>Management/Dose-modification	**Avelumab**<br>Management/Dose-modification
**Grade 1 :** Pre-hypertension (systolic BP 120-139 mmHg or diastolic BP 80-89 mmHg)	Continue treatment<br>Consider increased blood pressure monitoring<br>No Anti-hypertensive therapy	No dose modification
**Grade 2:** Systolic BP 140-159 mmHg or diastolic BP 90-99 mmHg<br>OR<br>Symptomatic increase by ≥ 20 mmHg (diastolic) if previously within normal limits	Continue treatment.<br>If symptomatic, hold treatment until symptoms resolve AND diastolic BP ≤ 90 mmHga. When treatment is restarted, continue at the same dose level.<br><br>Anti-hypertensive therapy : Treat with the aim to achieve diastolic BP ≤ 90 mmHg:<br>- If BP previously within normal limits, start anti-hypertensive monotherapy<br>- If patient already on anti-hypertensive medication, titrate up the dose	No dose modification
**Grade 3**: Systolic BP ≥ 160 mmHg or diastolic BP ≥100 mmHg<br>OR<br>More than one drug or more intensive therapy than previously used indicated	Hold treatment until diastolic BP ≤ 90 mmHg, and if symptomatic, until symptoms resolve. When treatment is restarted, continue at the same dose level.<br><br>If BP is not controlled with the addition of new or more intensive therapy, reduce by 1 dose level.<br><br>If Grade 3 hypertension recurs despite dose reduction and antihypertensive therapy, discontinue therapy<br><br>Anti-hypertensive therapy: Treat with the aim to achieve diastolic BP ≤ 90 mmHg:<br>- Start anti-hypertensive medication<br>AND/OR<br>- Increase current antihypertensive medication<br>AND/OR<br>- Add additional anti-hypertensive medications.	Hold treatment until diastolic BP ≤ 90 mmHg, and if symptomatic, until symptoms resolve. When treatment is restarted, continue at the same dose level.
**Grade 4** : Life-threatening consequences (eg, malignant hypertension, transient or permanent neurologic deficit, hypertensive crisis)	Discontinue therapy	No dose modification

[a] For cohort [A'], regorafenib should be interrupted and no dose reduction are allowed. Patients requiring a delay of > 4 weeks should permanently discontinue regorafenib

CONFIDENTIAL

**ALT and/or AST and/or bilirubin increases related**	**Regorafenib**[a]<br>Management/Dose-modification	**Avelumab**<br>Management/Dose-modification
< 3 times upper limit of normal (ULN) for ALT/AST and/or total bilirubin > ULN to<br>1.5 x ULN	Any occurrence<br>Continue Regorafenib treatment.<br>Monitor liver function weekly until transaminases return to <3 times ULN or baseline	Continue Avelumab therapy.<br>Continue liver function monitoring<br>If worsens:<br>Treat as appropriated ALT/AST increased (see below)
AST or ALT > 3.0 to ≤ 5 x ULN<br>and/or total bilirubin > 1.5 to ≤ 3 x ULN		Withhold Avelumab therapy<br><br>Increase frequency of monitoring to every 3 days<br>If returns to <3 times ULN or baseline: resume routine monitoring, resume Avelumab therapy<br>If elevation persists > 5-7 days or worsens : treat as appropriated ALT/AST increased (see below)
AST or ALT > 5 x ULN to ≤20 x ULN and/or total bilirubin > 3 x ULN to ≤ 10 x ULN	1st occurrence :<br>Interrupt Regorafenib treatment.<br>Monitor transaminases weekly until return to <3 times ULN or baseline.<br>Restart: If the potential benefit outweighs the risk of hepatotoxicity, decrease dose by one dose level, and monitor liver function weekly for at least 4 weeks.<br><br>Re-occurrence : Discontinue permanently	discontinue Avelumab therapy<br><br>Increase frequency of monitoring to every 1 to 2 days<br>1 to 2 mg/kg/day prednisolone or equivalent<br><br>Add prophylactic antibiotics for opportunistic infections<br>Consult gastroenterologist / hepatologist<br>Consider obtaining MRI/CT scan of liver and liver biopsy if clinically warranted<br><br>If returns to <3 times ULN or baseline:<br>Taper steroids over at least 1 month<br>If does not improve in > 3 to 5 days, worsens or rebounds:<br>Add mycophenolate mofetil 1 gram (g) twice daily<br>If no response within an additional 3 to 5 days , consider other immunosuppressants per local guidelines<br>Re-occurrence : Discontinue permanently
>20 times ULN<br>or **>3 times ULN with concurrent bilirubin > 2 times ULN**	Any occurrence<br>Discontinue permanently<br>Monitor liver function weekly until resolution or return to baseline.	

[a] For cohort [A’], regorafenib should be interrupted and no dose reduction are allowed. Patients requiring a delay of > 4 weeks should permanently discontinue regorafenib

CONFIDENTIAL

Diarrhea/colitis (NCI-CTCAE v5.0)	Regorafenib[a]	Avelumab	
		Initial Management	Follow up Management
Grade 1<br>Diarrhea: < 4 stools/day over baseline<br>Colitis: asymptomatic	No change	Continue Avelumab therapy<br>Symptomatic treatment (e.g. loperamide)	Close monitoring<br>Subject education<br>If worsens: treat as grade 2, 3 or 4
Grade 2<br>Diarrhea: 4 to 6 stools per day over baseline; IV fluids < 24h; not interfering with ADL<br>Colitis: abdominal pain; blood in stool	No change	Withhold Avelumab therapy<br>Symptomatic treatment	If improves ≤ grade 1: Resume Avelumab therapy<br>If persists >5-7 days or recurs, treat as grade 3 or 4
Grade 3 to 4<br>Diarrhea (Grade 3): ≥ 7 stools per day over Baseline; incontinence; IV fluids≥ 24h; interfering with ADL<br>Colitis (grade 3): severe abdominal pain, medical intervention indicated, peritoneal signs<br>Grade 4: life-threatening, perforation	Delay until ≤ grade 2 and reduce 1 dose level for grade 3<br><br>Permanently discontinue for grade 4, recurrent grade 3 or in case of no recovery after 4 weeks	Withhold for grade 3<br><br>Permanently discontinue for grade 4 or recurrent grade 3<br>1.0 to 2.0 mg/kg/day prednisone or equivalent add prophylactic antibiotics for opportunistic infections<br>Consider lower endoscopy	If improves:<br>Continue steroids until grade ≤1, then taper over at least 1 month; resume Avelumab therapy following steroids taper (for initial Grade 3).<br><br>If worsens persists > 3 to 5 days, or recur after improvement:<br>Add infliximab 5mg/kg (if no contraindication),<br>Note: infliximab should not be used if perforation or sepsis

[a] For cohort [A'], regorafenib should be interrupted and no dose reduction are allowed. Patients requiring a delay of > 4 weeks should permanently discontinue regorafenib

CONFIDENTIAL

Rash (NCI-CTCAE v5.0)	Regorafenib[a]	Avelumab	
		**Initial Management**	**Follow up Management**
Grade 1 to 2<br>Covering ≤ 30% body surface area	No change<br><br>Consider skin biopsy and dermatology consult<br><br>Consider 1.0 mg/kg/day prednisone or equivalent (max 1 week, taper steroids included) and/or dermocorticoids<br><br>In case of fever, consider infectious etiology<br>in case of pruritus, add antihistamine	Continue avelumab therapy<br>Symptomatic therapy (for example, antihistamines, topical steroids)	If Grade 2 persists > 1 to 2 weeks or recurs:<br>Withhold Avelumab therapy<br>Consider skin biopsy<br>Consider 0.5 - 1.0 mg/kg/day prednisone IV or equivalent<br>One improving, taper steroids over at least 1 month, consider prophylactic antibiotics for opportunistic infections, and resume Avelumab therapy following steroid tapers<br>If worsen: treat as grade 3 to 4
Grade 3 to 4<br>Grade 3: Covering ≥ 30% body surface area<br>Grade 4: life threatening consequences	Delay until ≤ grade 2 and reduce 1 dose level for grade 3<br><br>Consider skin biopsy and dermatology consult<br><br>Consider 1.0 mg/kg/day prednisone or equivalent (max 1 week, taper steroids included) and/or dermocorticoids<br><br>In case of fever, consider infectious etiology<br>in case of pruritus, add antihistamine<br><br>Permanently discontinue for grade 4, recurrent grade 3 or in case of no recovery after 4 weeks	Withhold for grade 3<br>Permanently discontinue for grade 4 or recurrent grade 3<br>Avelumab<br>Consider skin biopsy, Dermatology consult<br>1.0 to 2.0 mg/kg/day prednisone or equivalent<br>Add prophylactic antibiotics for opportunistic infections	If improves to grade ≤1:<br>taper steroids over at least 1 month,<br>Resume Avelumab therapy following steroid tapers (for initial grade 3)

[a] For cohort [A'], regorafenib should be interrupted and no dose reduction are allowed. Patients requiring a delay of > 4 weeks should permanently discontinue regorafenib

CONFIDENTIAL

Pneumonitis (NCI-CTCAE v5.0)	Regorafenib	Avelumab	
		**Initial Management**	**Follow up Management**
Grade 1<br>Radiographic changes only	No change	Consider withholding Avelumab therapy<br>Monitor for symptoms every 2 to 3 days<br>Consider Pulmonary and Infectious disease consults	Re-assess at least every 3 weeks<br>If worsens:<br>Treat as grade 2 or grade 3 or 4
Grade 2<br>Mild to moderate new symptoms	No change	Withhold Avelumab therapy<br>Pulmonary and Infectious disease consults<br>Monitor symptoms daily, consider hospitalization<br>1.0 to 2.0 mg/kg/day prednisone or equivalent<br>Add prophylactic antibiotics for opportunistic infections<br>Consider bronchoscopy, lung biopsy	Re-assess at least every 3 weeks<br>If improves:<br>When symptoms return to grade ≤ 1, taper steroids over at least 1 month and then resume Avelumab therapy and following steroid tapers<br>If not improving after 2 weeks or worsening or for recurrent grade 2: Treat as Grade 3 to 4.
Grade 3 to 4<br>Grade 3: Severe new symptoms;<br>New/worsering hypoxia<br>Grade 4: Life-threatening	Permanently discontinue	Permanently discontinue Avelumab therapy<br>Hospitalize<br>Pulmonary and Infectious disease consults<br>1.0 to 2.0 mg/kg/day prednisolone or equivalent<br>Add prophylactic antibiotics for opportunistic infections<br>Consider bronchoscopy, lung biopsy	If imporves to grade ≤ 1:<br>Taper steroids over at least 1 month<br>If not improving after 48 hours or worsening: Add additional immunosuppression (for example, infliximab, cyclophosphamide, Iv immunoglobulin, or mycophenolate mofetil)

CONFIDENTIAL

Endocrine Disorder	Regorafenib[a]	Avelumab	
		Initial Management	Follow-up Management
Grade 1 or Grade 2 endocrinopathies (hypothyroidism, hyperthyroidism, adrenal insufficiency, type I diabetes mellitus)	No change	Continue Avelumab therapy<br>Endocrinology consult if needed<br><br>Start thyroid hormone replacement therapy (for hypothyroidism), anti-thyroid treatment (for hyperthyroidism), corticosteroids (for adrenal insufficiency) or insulin (for Type I diabetes mellitus) as appropriate.<br><br>Rule-out secondary endocrinopathies (i.e. hypopituitarism / hypophysitis)	Continue hormone replacement/suppression and monitoring of endocrine function as appropriate.
Grade 3 or Grade 4 endocrinopathies (hypothyroidism, hyperthyroidism, adrenal insufficiency, type I diabetes mellitus)	Delay until ≤ grade 2 and reduce 1 dose level<br><br>For grade 4, permanent discontinuation can be considered at treating investigator discretion	Withhold Avelumab therapy<br>Consider hospitalization<br>Endocrinology consult<br><br>Start thyroid hormone replacement therapy (for hypothyroidism), anti-thyroid treatment (for hyperthyroidism), corticosteroids (for adrenal insufficiency) or insulin (for type I diabetes mellitus) as appropriate.<br><br>Rule-out secondary endocrinopathies (i.e. hypopituitarism / hypophysitis)	Resume Avelumab once symptoms and/or laboratory tests improve to Grade ≤ 1 (with or without hormone replacement/suppression).<br><br>Continue hormone replacement/suppression and monitoring of endocrine function as appropriate.
Hypopituitarism/Hypophysitis (secondary endocrinopathies)	No change	If secondary thyroid and/or adrenal insufficiency is confirmed (i.e. subnormal serum FT4 with inappropriately low TSH and/or low serum cortisol with inappropriately low ACTH) :<br>• Refer to endocrinologist for dynamic testing as indicated and measurement of other hormones (FSH, LH, GH/IGF-1, PRL, testosterone in men, estrogens in women)<br>• Hormone replacement/suppressive therapy as appropriate	Resume Avelumab once symptoms and hormone tests improve to Grade ≤ 1 (with or without hormone replacement).<br><br>In addition, for hypophysitis with abnormal MRI, resume Avelumab only once shrinkage of the pituitary gland on MRI/CT scan is documented.<br><br>Continue hormone replacement/suppression therapy as appropriate.

CONFIDENTIAL

Endocrine Disorder	Regorafenib[a]	Avelumab	
		Initial Management	Follow-up Management
		• Perform pituitary MRI and visual field examination as indicated<br><br>**If hypophysitis confirmed:**<br>• Continue Avelumab if mild symptoms with normal MRI. Repeat the MRI in 1 month<br>• Withhold Avelumab if moderate, severe or life-threatening symptoms of hypophysitis and/or abnormal MRI. Consider hospitalization. Initiate corticosteroids (1 to 2 mg/kg/day prednisone or equivalent) followed by corticosteroids taper during at least 1 month.<br>• Add prophylactic antibiotics for opportunistic infections.	

[a] For cohort [A'], regorafenib should be interrupted and no dose reduction are allowed. Patients requiring a delay of > 4 weeks should permanently discontinue regorafenib

CONFIDENTIAL

Grade of Creatinine Increased (NCI-CTCAE v5.)	Regorafenib[a]	Avelumab	
		Initial Management	Follow-up Management
**Grade 1**<br>Creatinine increased > ULN to 1.5 x ULN	No change	Continue Avelumab therapy	Continue renal function monitoring<br>If worsens:<br>Treat as Grade 2 to 3 or 4.
**Grade 2 to 3**<br>Creatinine increased > 1.5 and ≤ 6 x ULN	No change for grade 2<br>Delay until ≤ grade 2 and reduce 1 dose level for grade 3	Withhold Avelumab therapy<br>Increase frequency of monitoring to every 3 days<br>1.0 to 2.0 mg/kg/day prednisone or equivalent.<br>Add prophylactic antibiotics for opportunistic infections<br>Consider renal biopsy	If returns to Grade ≤1:<br>Taper steroids over at least 1 month, and resume Avelumab therapy following steroids taper.<br>If worsens:<br>Treat as Grade 4.
**Grade 4**<br>Creatinine increased > 6 x ULN	Permanently discontinue for grade 4	Permanently discontinue Avelumab therapy<br>Monitor creatinine daily<br>1.0 to 2.0 mg/kg/day prednisone or equivalent.<br>Add prophylactic antibiotics for opportunistic infections<br>Consider renal biopsy<br>Nephrology consult	If returns to Grade ≤1:<br>Taper steroids over at least 1 month.

[a] For cohort [A'], regorafenib should be interrupted and no dose reduction are allowed. Patients requiring a delay of > 4 weeks should permanently discontinue regorafenib

CONFIDENTIAL

Myocarditis	Initial Management	Follow-up Management
New onset of cardiac signs or symptoms and / or new laboratory cardiac biomarker elevations (e.g. troponin, CK-MB, BNP) or cardiac imaging abnormalities suggestive of myocarditis.	Withhold Avelumab therapy and Regorafenib.<br>Hospitalize.<br>In the presence of life threatening cardiac decompensation, consider transfer to a facility experienced in advanced heart failure and arrhythmia management.<br>Cardiology consult to establish etiology and rule-out immune-related myocarditis.<br>Guideline based supportive treatment as per cardiology consult.*<br><br>Consider myocardial biopsy if recommended per cardiology consult.	If symptoms improve and immune-related etiology is ruled out, re-start Avelumab therapy and Regorafenib (if delay < 4 weeks).<br><br>If symptoms do not improve/worsen, viral myocarditis is excluded, and immune-related etiology is suspected or confirmed following cardiology consult, manage as immune-related myocarditis.
Immune-related myocarditis	Permanently discontinue Avelumab and Regorafenib.<br>Guideline based supportive treatment as appropriate as per cardiology consult.*<br>1.0 to 2.0 mg/kg/day prednisone or equivalent<br>Add prophylactic antibiotics for opportunistic infections.	Once improving, taper steroids over at least 1<br><br>If no improvement or worsening, consider additional immunosuppressants (e.g. azathioprine, cyclosporine).
*Local guidelines, or eg. ESC or AHA guidelines<br>ESC guidelines website: https://www.escardio.org/Guidelines/Clinical-Practice-Guidelines<br>AHA guidelines website:<br>http://professional.heart.org/professional/GuidelinesStatements/searchresults.jsp?q=&y=&t=1001		

CONFIDENTIAL

Grade of other irAEs (NCI-CTCAE v5.)	Regorafenib[a]	Avelumab	
		**Initial Management**	**Follow-up Management**
Grade 2 or Grade 3 clinical signs or symptoms suggestive of a potential irAE	No change for grade 2<br><br>Delay until ≤ grade 2 and reduce 1 dose level for grade 3	Withhold Avelumab therapy pending clinical investigation	If irAE is ruled out, manage as appropriate according to the diagnosis and consider re-starting Avelumab therapy<br>If irAE is confirmed, treat as Grade 2 or 3 irAE.
Grade 2 irAE or first occurrence of Grade 3 irAE	No change for grade 2<br><br>Delay until ≤ grade 2 and reduce 1 dose level for grade 3	Withhold Avelumab therapy<br>1.0 to 2.0 mg/kg/day prednisone or equivalent<br>Add prophylactic antibiotics for opportunistic infections<br>Specialty consult as appropriate	If improves to Grade ≤ 1: Taper steroids over at least 1 month and resume Avelumab therapy following steroids taper.
Recurrence of same Grade 3 irAEs	Permanently discontinue	Permanently discontinue Avelumab therapy<br>1.0 to 2.0 mg/kg/day prednisone or equivalent<br>Add prophylactic antibiotics for opportunistic infections<br>Specialty consult as appropriate	If improves to Grade ≤ 1: Taper steroids over at least 1 month.
Grade 4	Permanently discontinue	Permanently discontinue Avelumab therapy<br>1.0 to 2.0 mg/kg/day prednisone or equivalent and/or other immunosuppressant as needed<br>Add prophylactic antibiotics for opportunistic infections<br>Specialty consult.	If improves to Grade ≤ 1: Taper steroids over at least 1 month
Requirement for 10 mg per day or greater prednisone or equivalent for more than 12 weeks for reasons other than hormonal replacement for adrenal insufficiency<br><br>Persistent Grade 2 or 3 irAE lasting 12 weeks or longer	Permanently discontinue	Permanently discontinue Avelumab therapy<br>Specialty consult	

Abbreviations: ACTH=adrenocorticotropic hormone; ADL=activities of daily living; ALT=alanine aminotransferase; AST=aspartate aminotransferase; BNP=B-type natriuretic peptide; CK-MB=creatine kinase muscle/brain; CT= computed tomography; FSH=follicle-stimulating hormone; GH=growth hormone; IGF-1=insulin-like growth factor 1; irAE=immune related adverse event; IV=intravenous; LH=luteinizing hormone; MRI=magnetic resonance imaging; NCI CTCAE=National Cancer Institute Common Terminology Criteria for Adverse Events; PRL=prolactin;FT4=thyroxine; TSH=thyroid stimulating hormone; ULN=upper limit of normal

[a] For cohort [A'], regorafenib should be interrupted and no dose reduction are allowed. Patients requiring a delay of > 4 weeks should permanently discontinue regorafenib

CONFIDENTIAL

APPENDIX 9: CONCOMITANT TREATMENTS

PROTOCOLE

REGOMUNE

Interactions médicamenteuses

Madame, Monsieur,

Dans le cadre de votre participation à l'étude REGOMUNE, il est nécessaire d'avoir des informations sur les éventuelles interactions médicamenteuses ou alimentaires durant votre traitement par **Regorafenib** et **Avelumab**.

Interactions médicamenteuses/alimentaires avec le regorafenib :

Durant le traitement par regorafenib, il est conseillé d'éviter la prise des médicaments suivants :

- ✓ Antibiotiques : rifampicine, clarithromycine, telithromycine,
- ✓ Antifongiques : itraconazole, ketoconazole, posaconazole et voriconazole,
- ✓ Antiépileptiques : phénytoïne, carbamazépine, phenobarbital,
- ✓ Anti-inflammatoire : acide méfénamique, diflunisal, acide niflumique
- ✓ Médicaments utilisés dans le traitement de l'hypercholestérolémie : rosuvastatine, fluvastatine, atorvastatine,
- ✓ Médicaments utilisés dans le traitement de certains cancers : méthotrexate,
- ✓ Médicaments utilisés pour fluidifier le sang : warfarine,
- ✓ Jus de pamplemousse,
- ✓ Millepertuis (traitement à base de plante contre la dépression).

Interactions médicamenteuses/alimentaires avec l'avelumab :

D'après les connaissances actuelles sur l'avelumab, aucune interaction n'est attendue.

Nous vous rappelons néanmoins que durant votre participation à l'étude, il est indispensable de consulter votre médecin avant toute prise médicamenteuse.

Nous vous remercions de votre précieuse collaboration.

REGOMUNE_Interactions médicamenteuses_V1.0_21.02.2018

APPENDIX 10: SUMMARY OF THE CHOI CRITERIA

Response	Definition
CR	Disappearance of all lesions
	No new lesions
PR	A decrease in size ≥10% or a decrease in tumour attenuation (HU) ≥15% on CT
	No new lesions
	No obvious progression of non-measurable disease
SD	Does not meet criteria for CR, PR, or PD
	No symptomatic deterioration attributed to tumour progression
PD	An increase in tumour size ≥10% and does not meet criteria of PR by tumour attenuation on CT
	New lesions

Abbreviations: CR=complete response; PR=partial response; SD=stable disease; PD=progressive disease; HU=Hounsfield unit.

CONFIDENTIAL
